# Supplementary material for: A double-agent microRNA regulates viral cross-kingdom infection in animals and plants
Source: EMBO J. 2025 Mar 5;44(9):2446–72. doi: 10.1038/s44318-025-00405-4 (PMC12048567; doi:10.1038/s44318-025-00405-4)
Supplement: Supplementary file 1 — Appendix [file 44318_2025_405_MOESM1_ESM.pdf]

Appendix for

A double-agent microRNA regulates viral cross-kingdom infection in animals and plants

Wan Zhao *et al*.

*Corresponding authors Email: zhaow@ioz.ac.cn, cuif@ioz.ac.cn

This PDF file includes:

Appendix Table S1...page 2

Appendix Table S1.

Primers and probes used in the study.

Name	Sequence (5' to 3')
Primers for qPCR	
*Ls*U6-q-F	TGGAACGATACAGAGAAGATTAGCA
*Ls*U6-q-R	AACGCTTCACGATTTTGCGT
miR263-q-F	GGCGGGAATGGCACTGGAAGAAT
EF2-q-F	GTCTCCACGGATGGGCT
EF2-q-R	ATCTTGAATTTCTCGGCATACAT
NP-q-F	AGTGCTGATCGTATTGACAGA
NP-q-R	GATGAAGTACACAACTGGTC
Caspase1a-q-F	CCATCAACCGATTCCGATAACT
Caspase1a-q-R	TGCTGCTGCTGCTGTCAT
Caspase1b-q-F	GCCTGGTAGTAGCAGTCTTGA
Caspase1b-q-R	AGAGTGATTCCGCCATCTAGTT
Caspase2-q-F	GAGTCACGGCGGAATAATAGAG
Caspase2-q-R	TGAACGGTGCGACAATATCTC
Caspase8-q-F	TTCAAGCATGTCAAGGCAACT
Caspase8-q-R	ATGGTAGGCAGTCCGTGTT
CathBL-q-F	AGAGCTGATGTCGTGCTGCTC
CathBL-q-R	GCTGAACAGTTTGGCTTCGG
SPS5-q-F	CGACTCTTGCCGCCCACCAAC
SPS5-q-R	CAGCAGACTACCGGCTCAGCC
NTFYb-q-F	GCTCCAGCAAACAACGACTCG
NTFYb-q-R	CTTGGTGTAGGCAGCCTTCTTC
Dsimw501-q-F	CCGTACCGAACAACGCCG
Dsimw501-q-R	TCATGAGCACGTCGTGCG
UGT2B5-q-F	GCCATCCTACAGTCATCAACAG
UGT2B5-q-R	CCAAGGTCGATGTCTCTGTGA
*Os*U6-q-F	TACAGATAAGATTAGCATGGCCCC
*Os*U6-q-R	GGACCATTTCTCGATTTGTACGTG
*Os*Ubq10-q-F	TCACCTACGTCTACAACCAG
*Os*Ubq10-q-R	AGTGCTGATCGTATTGACAGA
GATA19-q-F	TCCCACCCATCCACCACAC
GATA19-q-R	CATCGGCTCGGGATCGG
priGATA19-F	AGCTGATGTATTCACCATGTG
priGATA19-R	CCAAGTACCAATAGAACAGCC
ExGATA19-F	ATCTCCACCGTCCGATCC

ExGATA19-R	CCAAGTACCAATAGAACAGCC
*Os*07g11600.1-q-F	GACCATTAAGTCATCCTCGAG
*Os*07g11600.1-q-R	TCGTCGCCATCGTTGTCTG
*Os*01g45800.1-q-F	TCCAACAACTCCCCGAGG
*Os*01g45800.1-q-R	AAGCTGTTGACGATGTGGTC
*Os*05g43100.1-q-F	GGTACAAAAGACCTAGTTGACTTC
*Os*05g43100.1-q-R	CGACTCCTGCATAAGTACAG
*Os*08g24860.1-q-F	TGCCGAACACACCGTTGC
*Os*08g24860.1-q-R	GACTTGATGGTCTTCTCGAG
*Os*12g06770.1-q-F	TTTAGTTCGTTTAATGGCTGGAGG
*Os*12g06770.1-q-R	TGCACGAATCCATTGTGCAG
*Os*12g06750.1-q-F	GCTGCTACTTTCTTCATGGTTC
*Os*12g06750.1-q-R	GCCTCTGCCTTGCATTTTGC
*Os*04g25360.2-q-F	TCTACCTCGCCGACATCAG
*Os*04g25360.2-q-R	TGCGACTGTGATTATGCTGG
*Os*03g44140.1-q-F	CACCTCCCTCTTGATAGATTACTG
*Os*03g44140.1-q-R	TCTACAACCACTTGGTTGTACC
*Os*01g10790.1-q-F	CTTCTGTGTGTTTCAAAGCAGG
*Os*01g10790.1-q-R	TTTGGAGAAATCCCACACAGC
*Os*01g31100.1-q-F	TTTAGTTCGTTTAATGGCGCAGG
*Os*01g31100.1-q-R	AGCTAGATATTCTTCCACGAGG
*Os*12g34690.1-q-F	ATGAACACGGATGGCACATTC
*Os*12g34690.1-q-R	TCAGGAGTTGTCCTTCTTTCG
*Os*08g21480.1-q-F	TGGCTTGTCATGGTTCGATC
*Os*08g21480.1-q-R	TCATGTAAGCTCCCTTAGACAC
*Os*08g21470.1-q-F	GTGGTTCTACATCAACCGTG
*Os*08g21470.1-q-R	TGAAGTCCAGGGCTTTAACTG
*Os*01g68050.1-q-F	CTACGTGCTGAGCTTCTCC
*Os*01g68050.1-q-R	TGTTTCCGAGTATCAGGGTG
*Os*02g12040.1-q-F	AGTTCTTGGCAAATAGCTCATCC
*Os*02g12040.1-q-R	GAACCAGGGAAGAAAGGTTG
*Os*11g05140.1-q-F	ATGATGCATGCGTTGCATGG
*Os*11g05140.1-q-R	GCAATCTGCTGAAGTTTGTCC
JAMYB-q-F	CCGAGCATGG TGACTAGCTCATCTT
JAMYB-q-R	CCTTGCACCC AACCGTTAAGCTGTT
COI1A-q-F	TACCTACATGGGAACAAATGAG
COI1A-q-R	CAAGTAGCACAAGCCGAAAG
COI1B-q-F	GTAATGTTGGGGAAACAGATG
COI1B-q-R	AAGCTTGCTCACTGAAGCAACAA

COI2-q-F	CCAAGCTTGCAGAAATTGGA
COI2-q-R	TCGCACCATTGCCATTAGAT
MYC2-q-F	AGCTCAACCAGCGCTTCTAC
MYC2-q-R	CCTTCTTGAGCGACTCCATC
CM-LOX1-q-F	ATGGCCGGAACAAGGATAG
CM-LOX1-q-R	TCAGATGGATGTGCTGTTGG
CM-LOX2-q-F	GTACGCTGGGTTCACAGCTC
CM-LOX2-q-R	TCAGATGGATGTGCTGTTGG
AOS2-q-F	GTTGACAACAAGCAGTGCCC
AOS2-q-R	CGGAGGTTGAAGCTTTGGTG
JMT1-q-F	AGCAAGTATGCTGAGAGCCG
JMT1-q-R	CGAGGGTTTTGTGCAAGCTC
JAZ8-q-F	CCAAACACGGCGGAAACAG
JAZ8-q-R	GGTGGACGGGAAGTTCTCAAAG
JAZ10-q-F	ATACCCATGGCGACGTAGAG
JAZ10-q-R	AAGGGGTTTCCCATCAATTC
JAZ12-q-F	ATCTGCCCGGTTTAGAGGAG
JAZ12-q-R	GGGCCAAAGAAATCTCAAAC
Primers for plasmid construction	
SPS5-tar-F	GCACACTGTGCTGTTACATC
SPS5-tar-R	CACTAGACGCTAATGAAGGC
XhoI-SPS5-tar-F	CCGCTCGAGGCACACTGTG
NotI-SPS5-tar-R	GCGGCCGCCACTAGACG
CathBL-tar-F	AATGTCTTAATACGCTTCTAATG
CathBL-tar-R	TCTTGAATTGTGTTCTCCTG
XhoI-CathBL-tar-F	CCGCTCGAGAATGTCTTAATACGCTTCTAATG
NotI-CathBL-tar-R	GCGGCCGCTCTTGAATTGTGTTCTCCTG
NTFYb-tar-F	CTTGTTCAGCAAGAATTGAAG
NTFYb-tar-R	CAACTCTTACTGAACAAAAGCC
XhoI-NTFYb-tar-F	CCGCTCGAGCTTGTTCAGCA
NotI-NTFYb-tar-R	GCGGCCGCCAACTCTTACTG
CathBL-5’UTR-F	AATGTCTTAATACGCTTCTAATG
CathBL-CDS-R	TTTCTTCCAGTTGGGTTTAC
pAC-CathBL5-F	GGGGTACCAATGTCTTAATACGCTTCTAATG
pAC-CathBL-R	CCGCTCGAGTTTCTTCCAGTTGG
Pre-miR1876-F	CCCACAGTCTCATATTAAGG
Pre-miR1876-R	CTAGTACAGGACTAGGAAG
EGFP-GATA19tar-F	TATATTCTTCCAGTGCCATTGAATTTCCCCGATCGTTCAA
EGFP-Insert-R	GGGCCGCTTTACTTGTACAGCTCGTCCATG

Vector-F	CCTCTAGAGTCCCCCGTGTTCTCTCC
Vector-R	GCTGTAGCCGACGATGGTGCGCC
EIE-infusion-F	CCTCTAGAGTCCCCCTCCGCGGCCC
EIE-infusion-R	GCTGTAGCCGACGATTTATTCATCGAATGTG
Ex-In-Ex-F	TCCGCGGCCCATCTCCAC
Ex-In-Ex-R	TTATTCATCGAATGTGGTAGGTACAGCC
Ex-In-Ex-MT-F	GAAAGCGATCCTATCAATATTCTGTGCACATTGAAAATG
Ex-In-Ex-MT-R	TGATTCATCAAAGGTACATTTCTCTAAACTTG
JAZ1-Lic-F	CGACGACAAGACCGTCACCATGGATCTGTTGG
JAZ1-Lic-R	GAGGAGAAGAGCCGTCGGGCAAGGCCCAG
GATA19-Lic-F	CGACGACAAGACCGTCACCATGGCTGCTGAAC
GATA19-Lic-R	GAGGAGAAGAGCCGTCGCGTCGAGTCCAACC
myc2-orf-F	ATGAACCTTTGGACGGACGACAAC
myc2-orf-R	CCGGGCGGCGGTGCCA
jaz1-orf-F	ATGGATCTGTTGGAGAAGAAGAAC
jaz1-orf-R	CTGGGCCTTGCCCTCAG
jaz8-orf-F	ATGGCCGGCCGTGCGAC
jaz8-orf-R	TATCTCCTGCTTTATTGTCATCTCTTGG
jaz10-orf-F	ATGGCGATGGAGGGGAAG
jaz10-orf-R	CAGCGCGATGGTGAGGC
GATA19-orf-F	ATGGCGGCGGAGCCCC
GATA19-orf-R	CGAGGTAGCCATGCTATCTTC
EcoRv-Myc2-Spe-F	GATATCATGAACCTTTGGACGGACGACAAC
EcoRv-Myc2-Spe-R	GACTAGTTTACTTGTCGTCATCGTCTTTGTAGTCCCGGGCGGCG
Xho-jaz1-BamH-F	CCGCTCGAGATGGATCTGTTGGAGAAGAAGAAC
Xho-jaz1-BamH-R	CGGGATCCTTAGTGATGATGATGATGATGCTGGGCCTTGCCC
Spe-jaz8-BamH-F	GACTAGTATGGCCGGCCGTGCGAC
Spe-jaz8-BamH-R	CGGGATCCTTAGTGATGATGATGATGATGTATCTCCTGCTTTAT
Xho-jaz10-BamH-F	CCGCTCGAGATGGCGATGGAGGGGAAG
Xho-jaz10-BamH-R	CGGGATCCTTAGTGATGATGATGATGATGCAGCGCGATGGTG
Xho-GA19-BamH-F	CCGCTCGAGATGGCGGCGGAGCCCC
Xho-GA19-BamH-R	CGGGATCCTTAGTGATGATGATGATGATGCGAGGTAGCCATGC
Pri-Osa-miR1876-F	CCCACAGTCTCATATTAAGG
Pri-Osa-miR1876-R	CTAGTACAGGACTAGGAAGTG
Primers for RNAi	
GFP-RNAi-F	CACAAGTTCAGCGTGTCCG
GFP-RNAi-R	GTTCACCTTGATGCCGTTC
T7-GFP-RNAi-F	TAATACGACTCACTATAGGCACAAGTTCAGCGTGTCCG
T7-GFP-RNAi-R	TAATACGACTCACTATAGGGTTCACCTTGATGCCGTTC

*Ls*CathBL-RNAi-F	TTGTCGGTGCGATCAGTGC
*Ls*CathBL-RNAi-R	GTTTGATGTAGACCCAGGCG
T7-LsCathBL-RNAi-F	TAATACGACTCACTATAGGTTGTCGGTGCGATCAGTGC
T7-LsCathBL-RNAi-R	TAATACGACTCACTATAGGGTTTGATGTAGACCCAGGCG
Primers for 5’RLM-RACE	
GATA-5’inner	GGCTCCAACTTTCCAATGAAG
GATA-5’outer	CCACCTTGTAAGCATTGGATC
Retro-5’inner	TTCTTGGTCCTAAGCATGGCAAC
Retro-5’outer	GGATTGGTCTAGGATCATGTTCAAC
Primers for generating mRNA Northern blot probes	
*Dm*Actin-probe-F	ACCGTGCTCAATGGGGTAC
*Dm*Actin-probe-R	GTGTGACGAAGAGGTTGCAG
CathBL-probe-F	GCTGAACAGTTTGGCTTCGG
CathBL-probe-F	AGAGCTGATGTCGTGCTGCTC
MiRNA Northern blot probes	
*Ls*U6	biotin-AACGCTTCACGATTTTGCGT-biotin
*Os*U6	biotin-GGACCATTTCTCGATTTGTACGTG-biotin
miR-263a	biotin- CCCGTGAATTCTTCCAGTGCCATT -biotin

F, forward primers; R, reverse primers.
