# Supplementary figures and images for: A double-agent microRNA regulates viral cross-kingdom infection in animals and plants
Source: EMBO J. 2025 Mar 5;44(9):2446–72. doi: 10.1038/s44318-025-00405-4 (PMC12048567; doi:10.1038/s44318-025-00405-4)

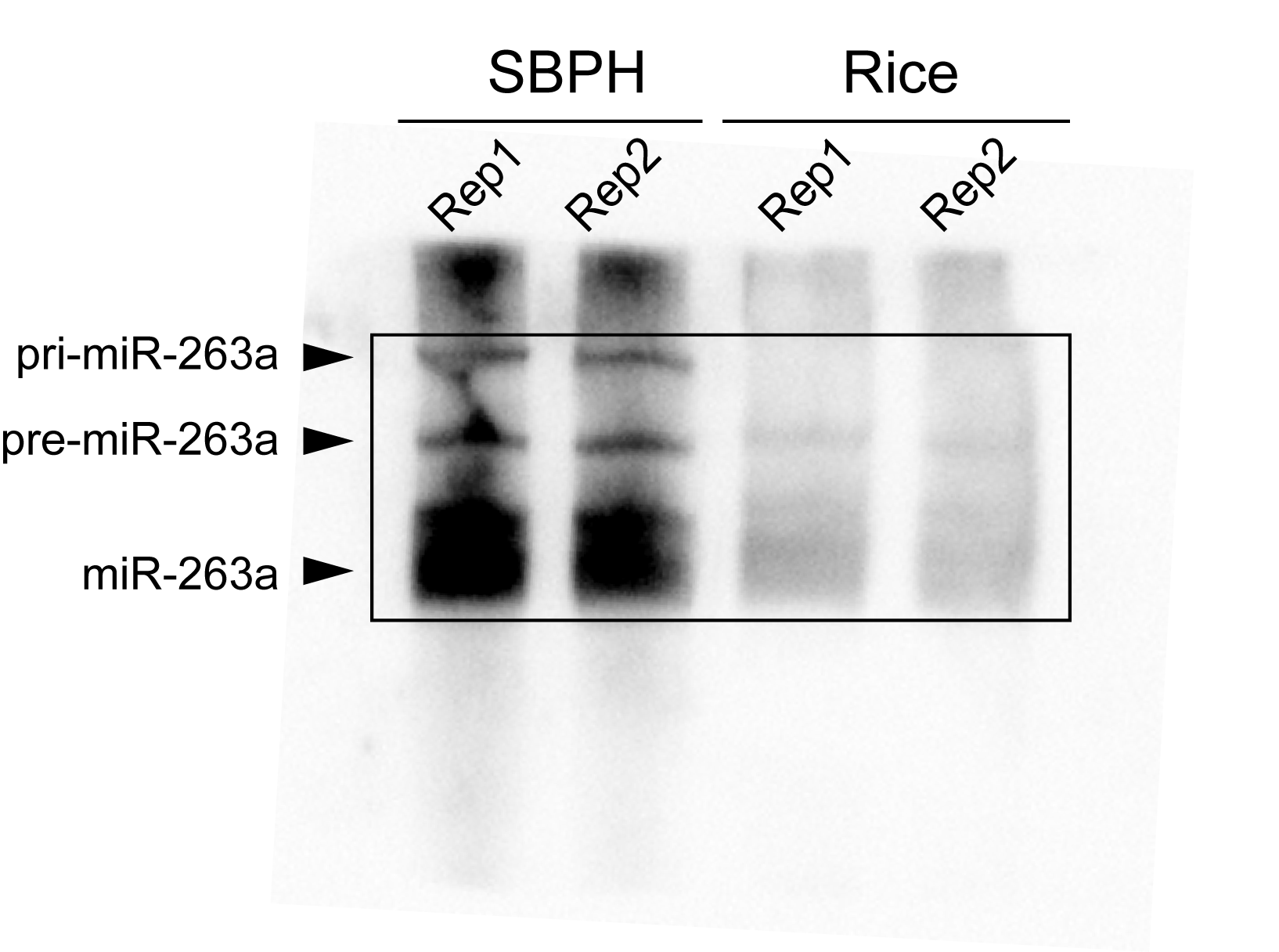

Supplement: Supplementary file 5 — Source data Fig. 1 [file 44318_2025_405_MOESM5_ESM.zip › Figure 1/1A/Northern blot miR-263a (top).tif]

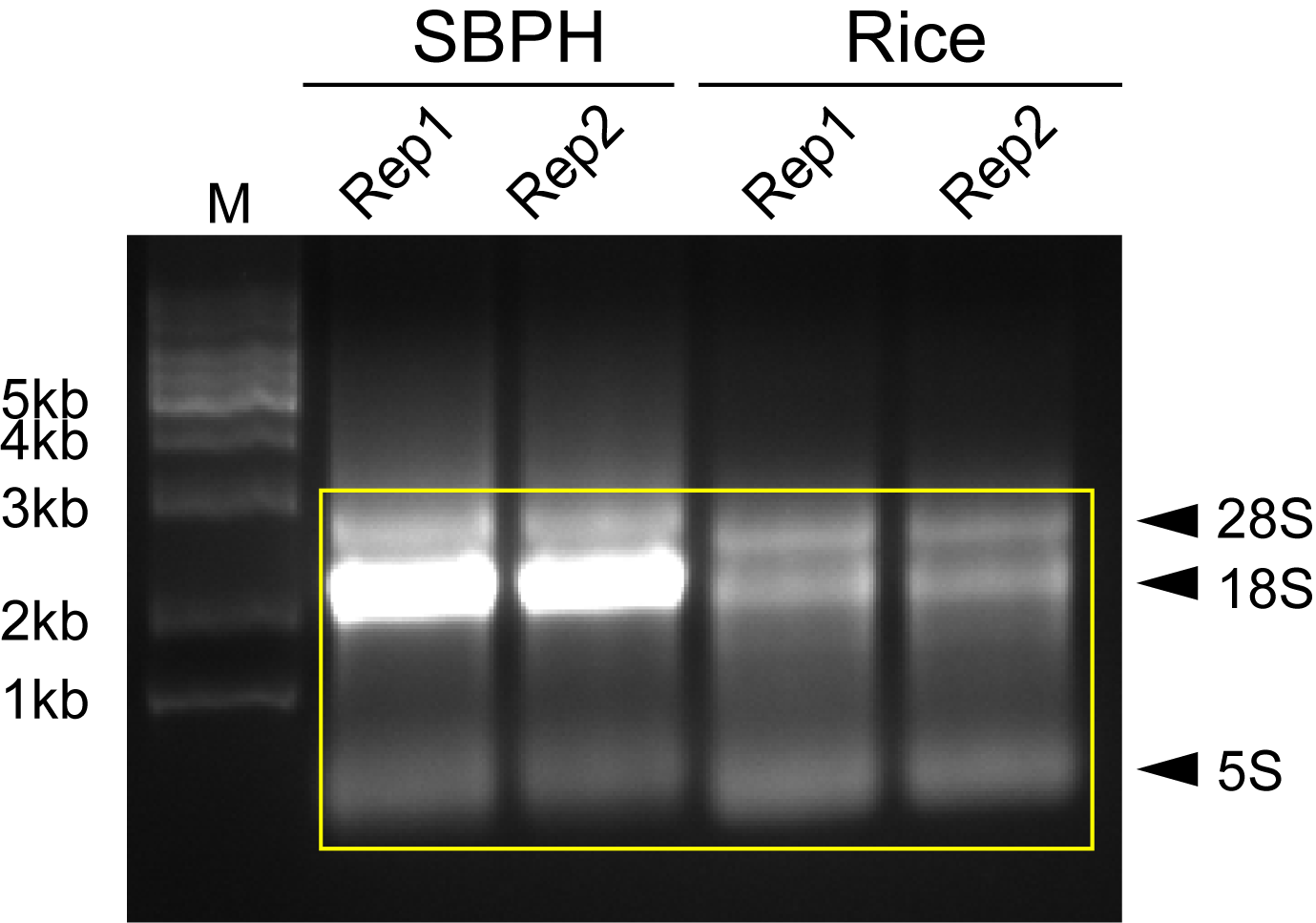

Supplement: Supplementary file 5 — Source data Fig. 1 [file 44318_2025_405_MOESM5_ESM.zip › Figure 1/1A/Gel-rRNA (bottom).tif]

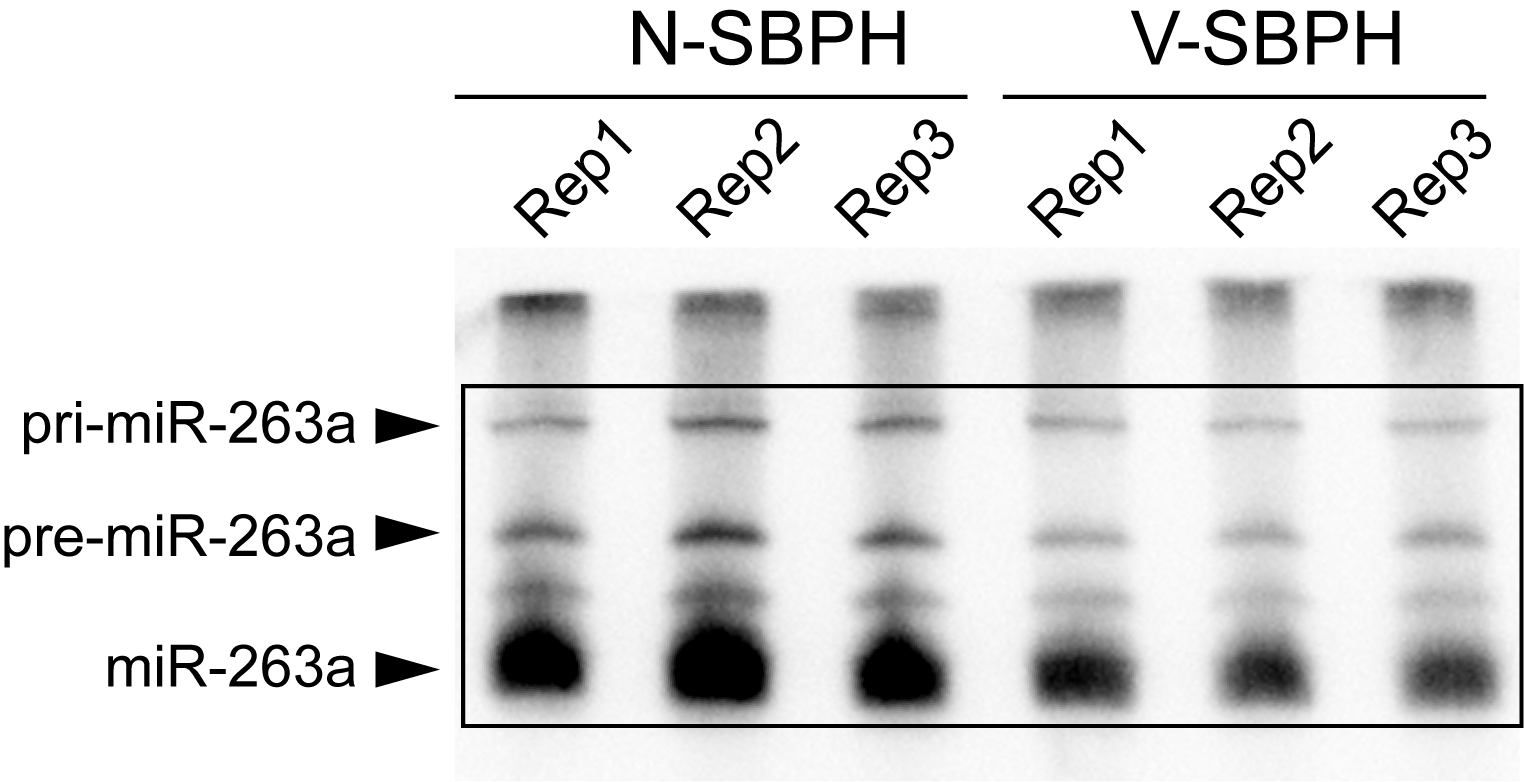

Supplement: Supplementary file 5 — Source data Fig. 1 [file 44318_2025_405_MOESM5_ESM.zip › Figure 1/1C/Northern blot miR-263a (top).tif]

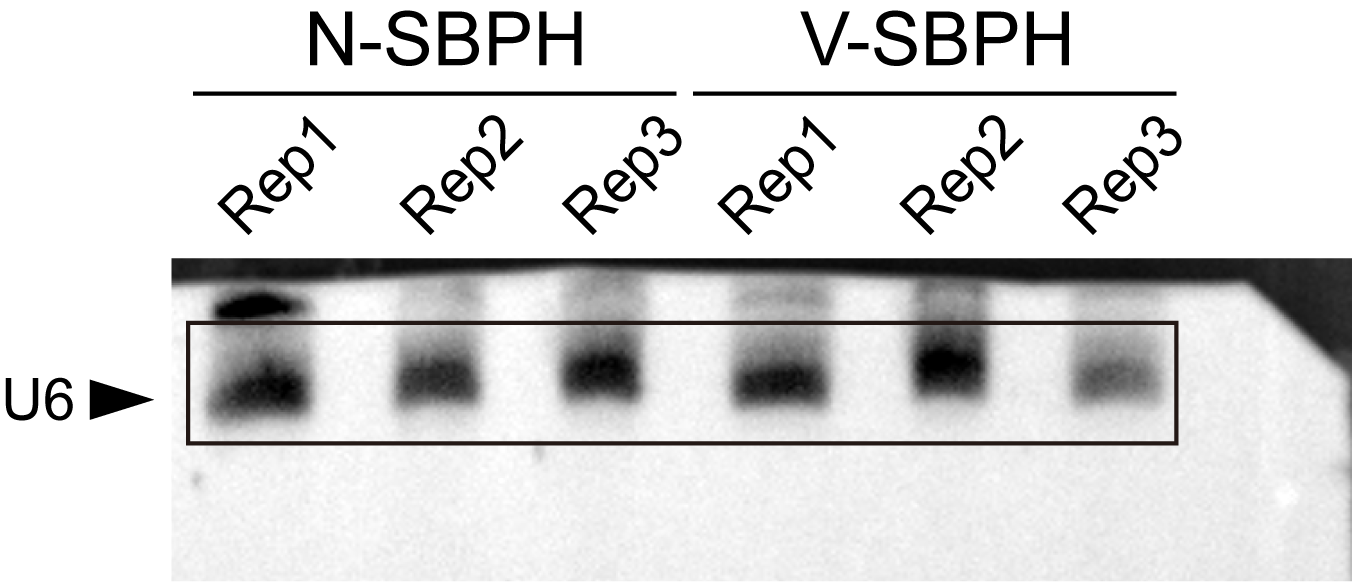

Supplement: Supplementary file 5 — Source data Fig. 1 [file 44318_2025_405_MOESM5_ESM.zip › Figure 1/1C/Northern blot U6 (bottom).tif]

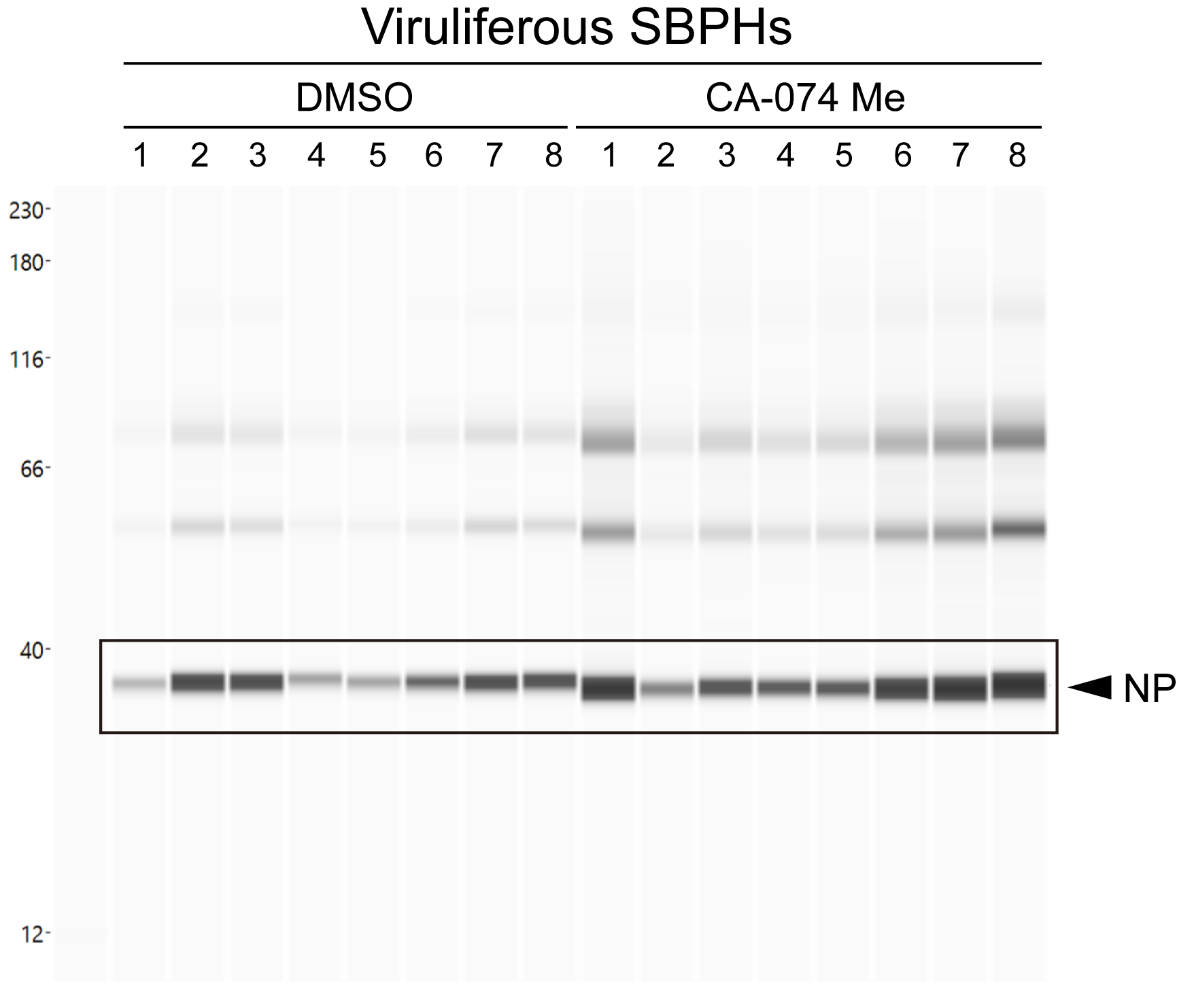

Supplement: Supplementary file 6 — Source data Fig. 2 [file 44318_2025_405_MOESM6_ESM.zip › Figure 2/2G/Western NP-DMSO, CA-074Me (bottom).tif]

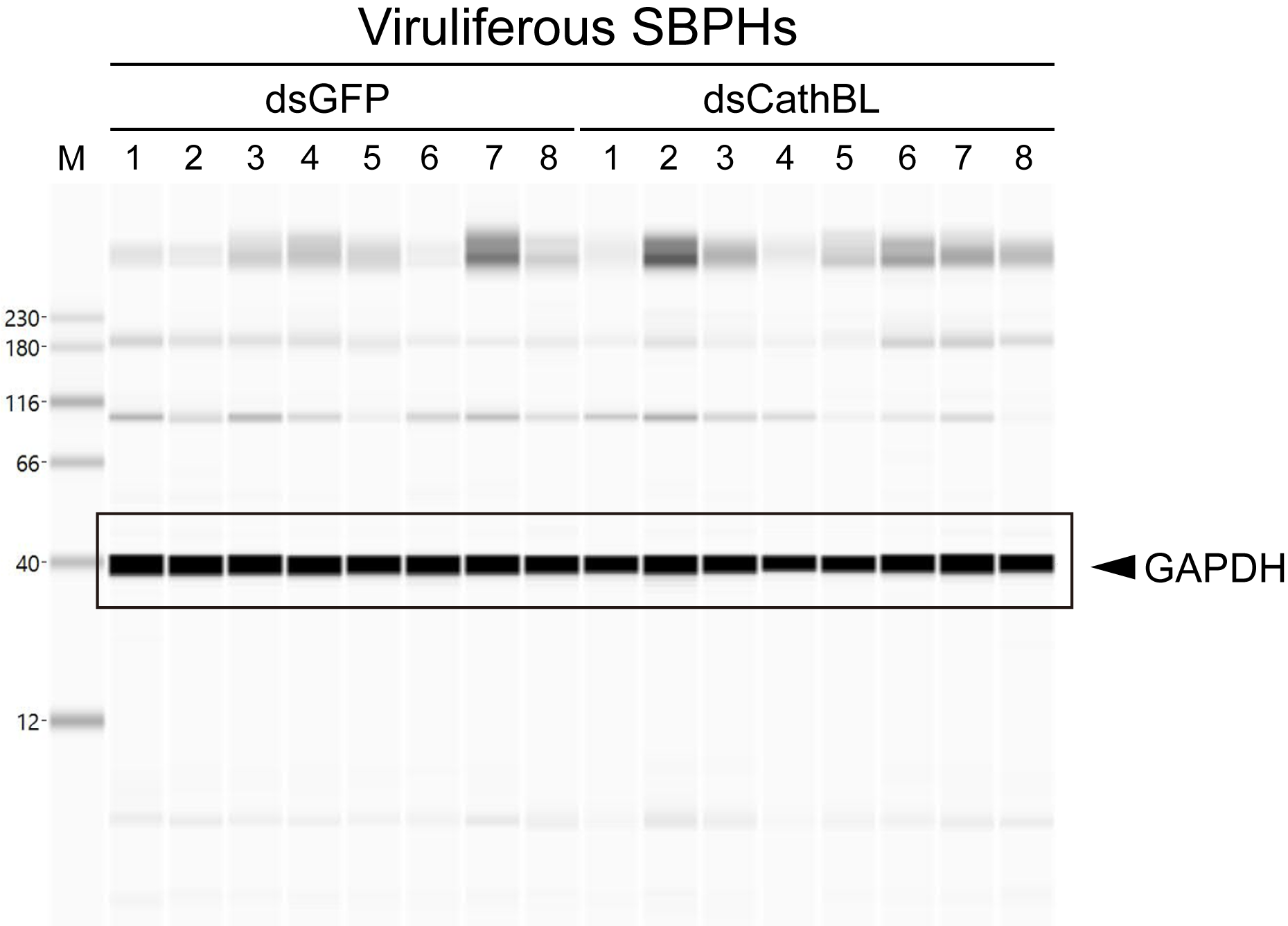

Supplement: Supplementary file 6 — Source data Fig. 2 [file 44318_2025_405_MOESM6_ESM.zip › Figure 2/2G/Western GAPDH-dsGFP, dsCathBL (top).tif]

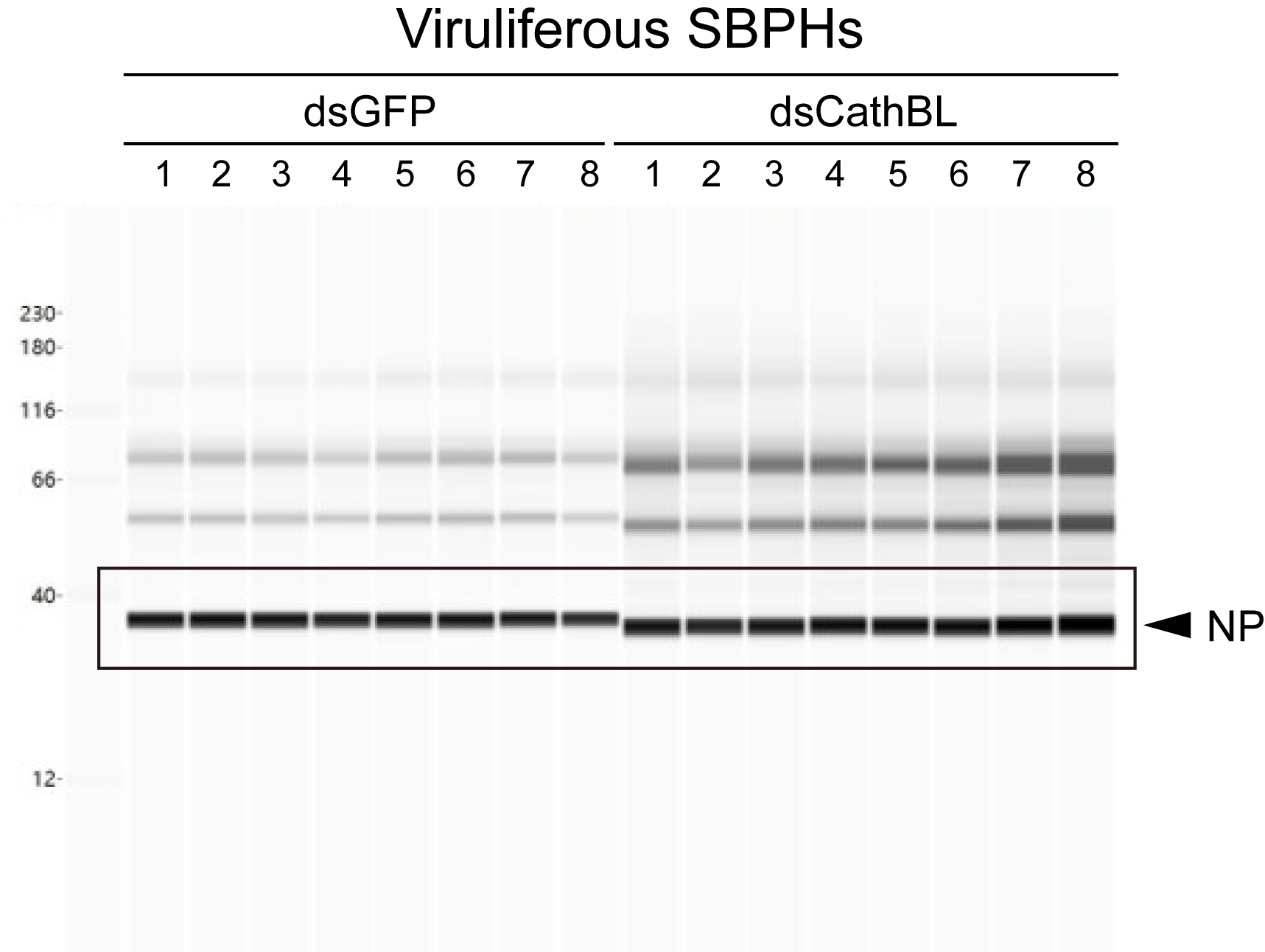

Supplement: Supplementary file 6 — Source data Fig. 2 [file 44318_2025_405_MOESM6_ESM.zip › Figure 2/2G/Western NP-dsGFP, dsCathBL (top).tif]

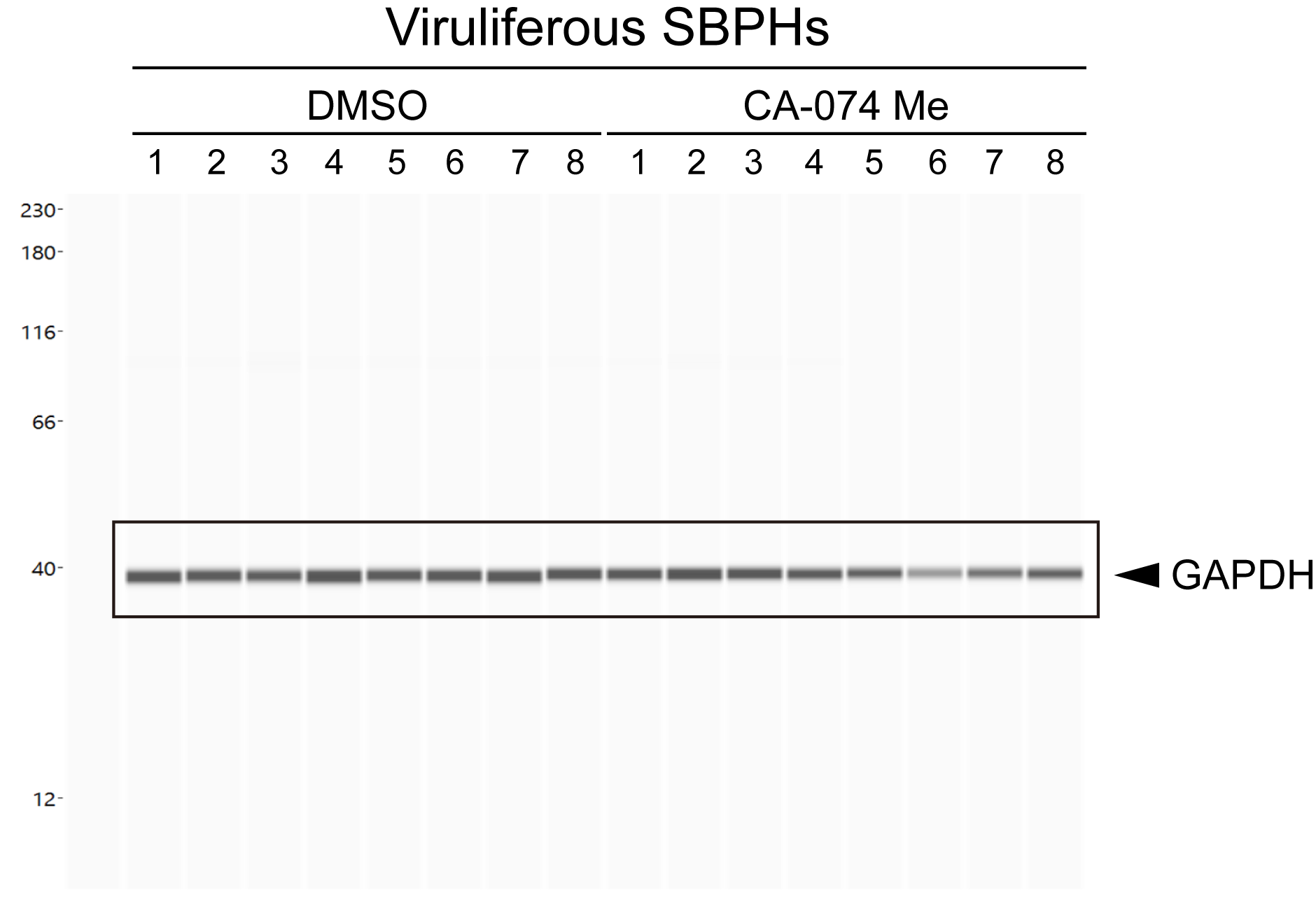

Supplement: Supplementary file 6 — Source data Fig. 2 [file 44318_2025_405_MOESM6_ESM.zip › Figure 2/2G/Western GAPDH-DMSO, CA-074Me (bottom).tif]

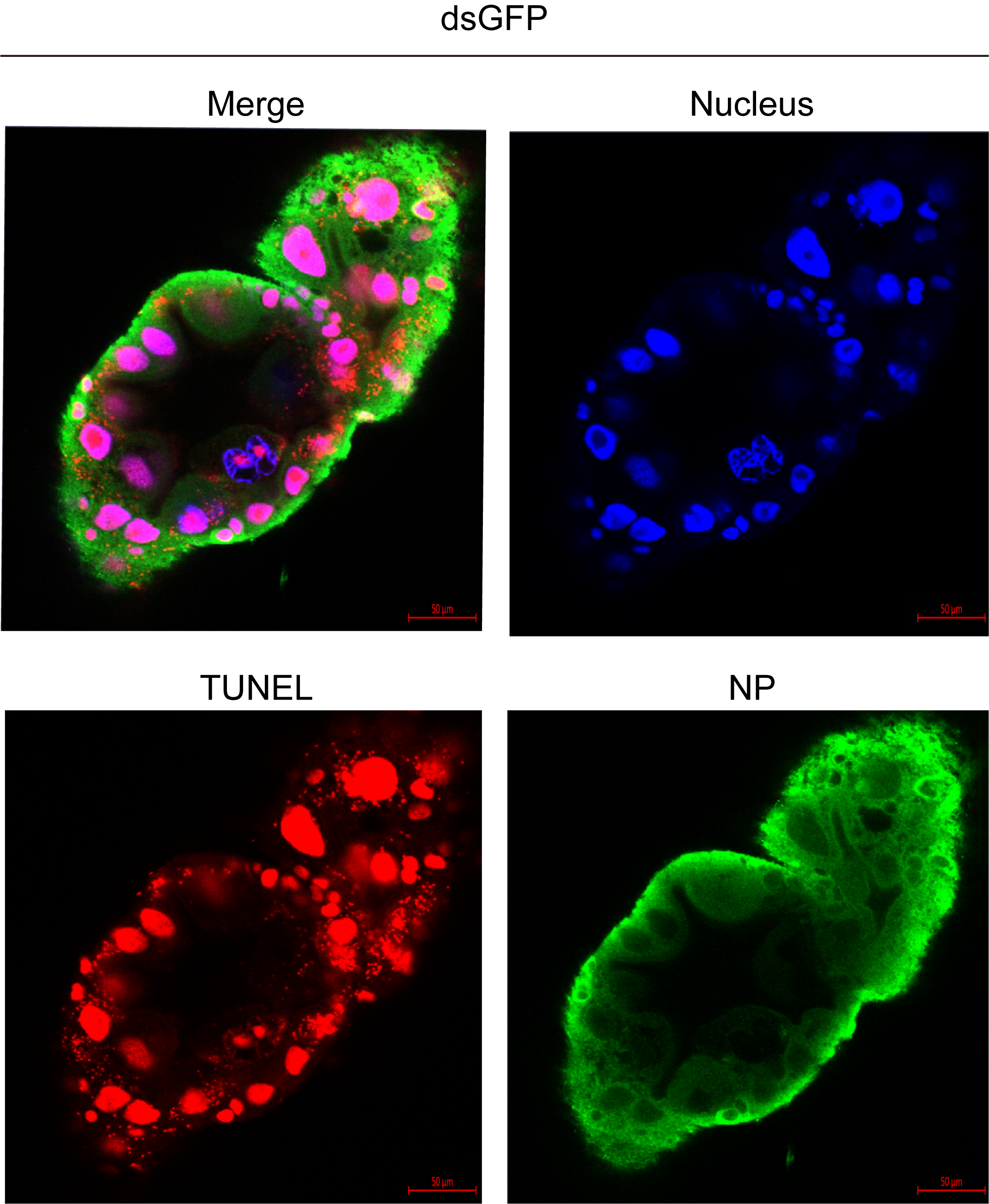

Supplement: Supplementary file 6 — Source data Fig. 2 [file 44318_2025_405_MOESM6_ESM.zip › Figure 2/2I/2I-Micr.image-Merge, Nucleus, TUNEL, NP (dsGFP).tif]

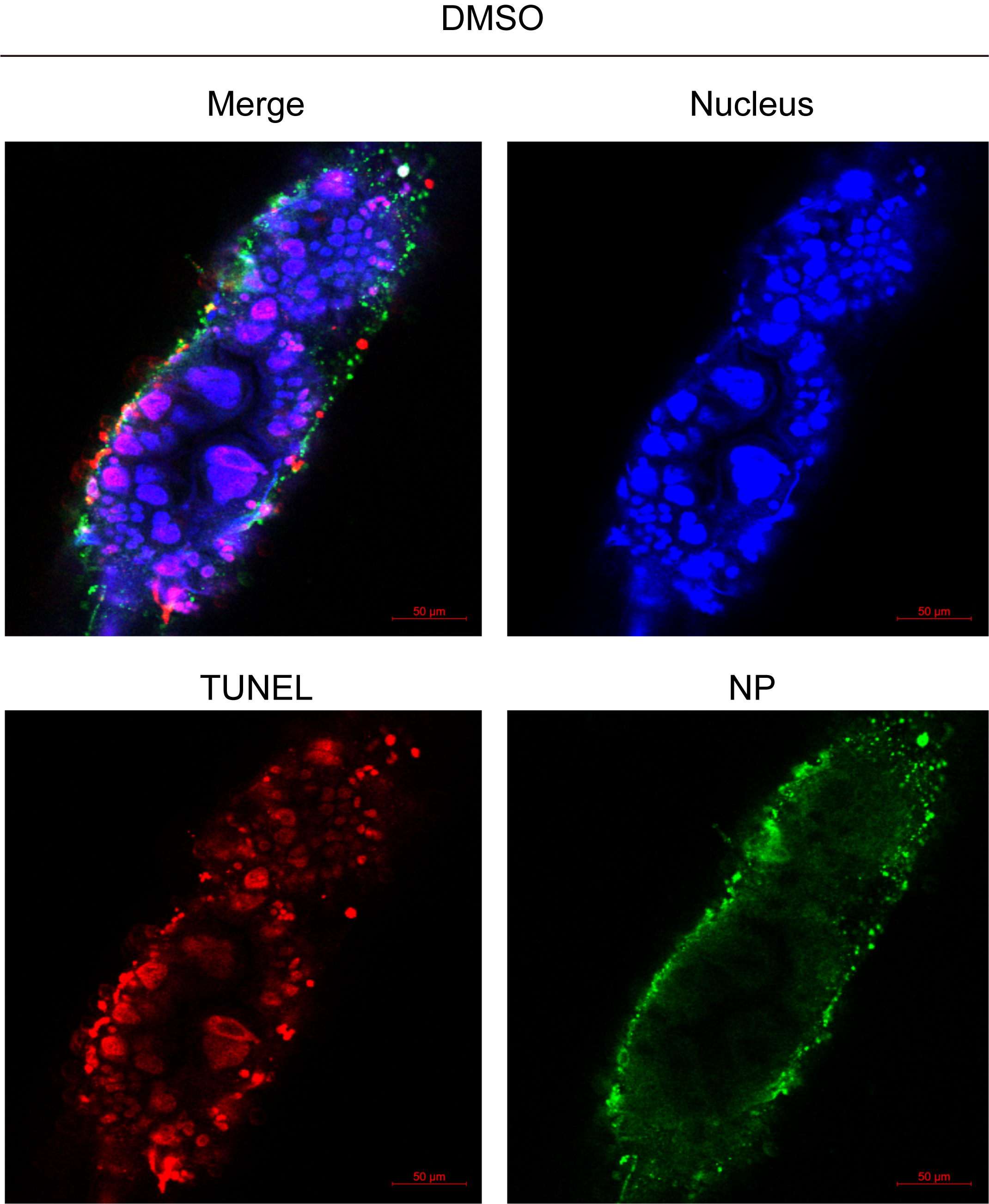

Supplement: Supplementary file 6 — Source data Fig. 2 [file 44318_2025_405_MOESM6_ESM.zip › Figure 2/2I/2I-Micr.image-Merge, Nucleus, TUNEL, NP (DMSO).tif]

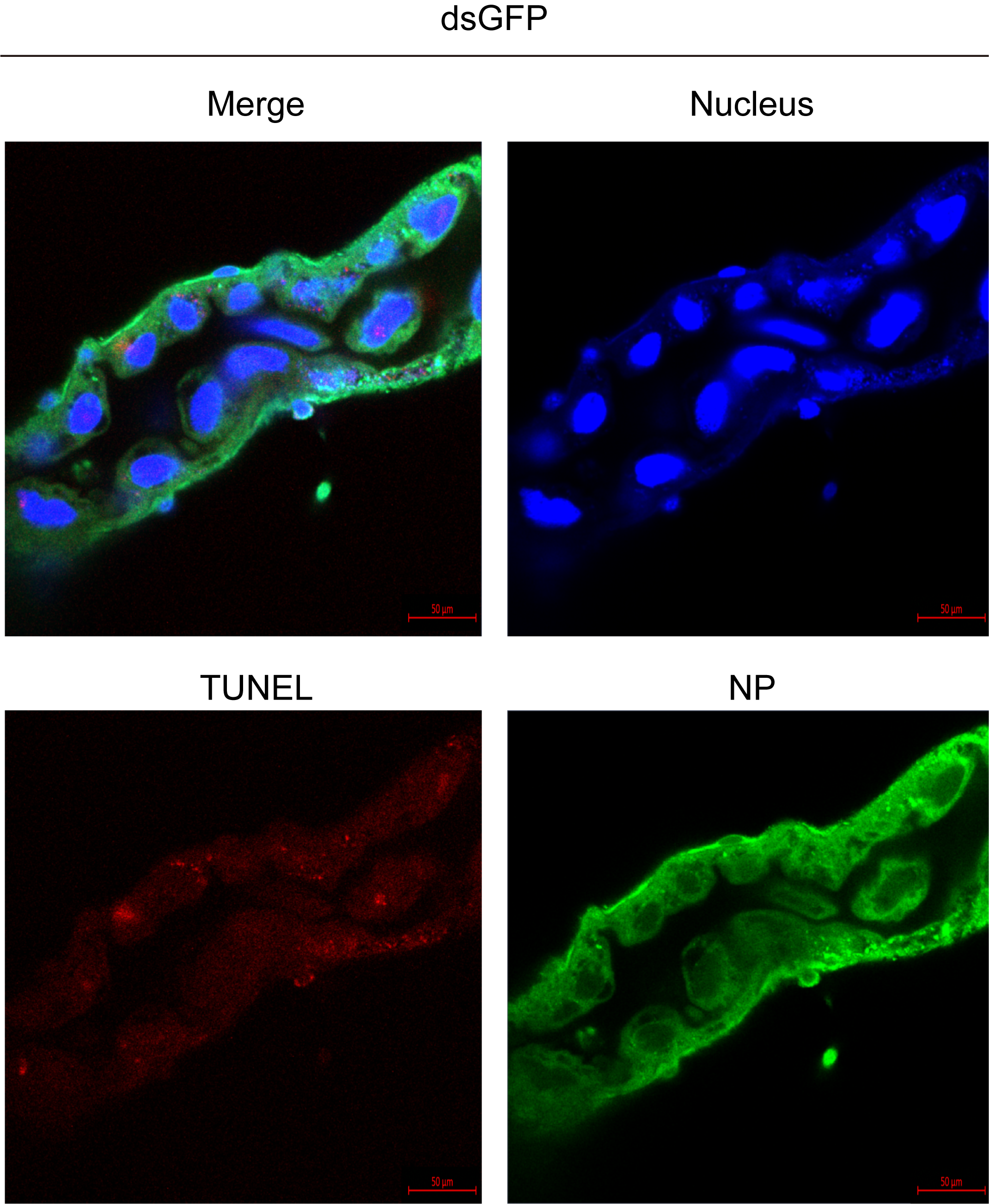

Supplement: Supplementary file 6 — Source data Fig. 2 [file 44318_2025_405_MOESM6_ESM.zip › Figure 2/2I/2I-Micr.image-Merge, Nucleus, TUNEL, NP (dsCathBL).tif]

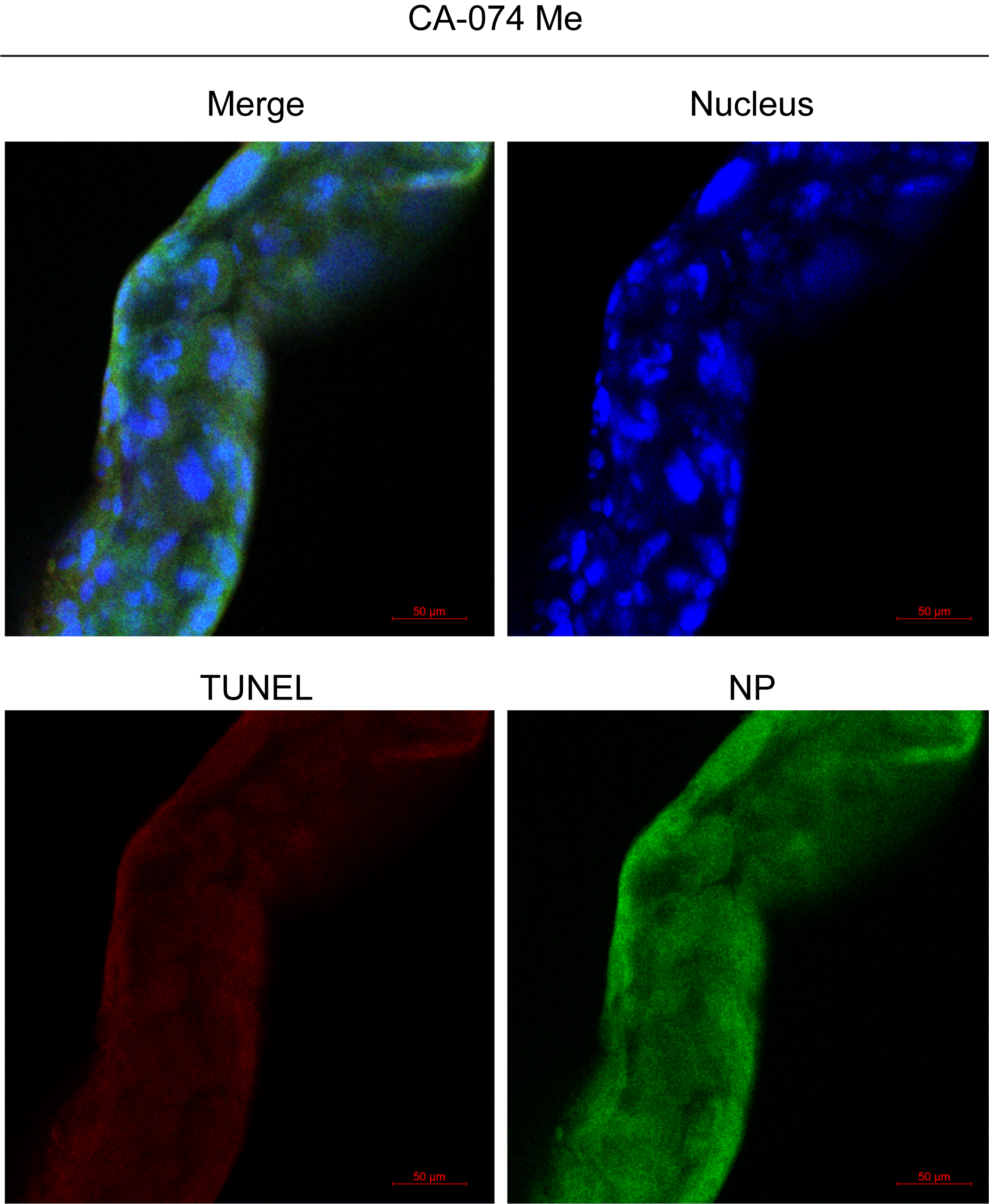

Supplement: Supplementary file 6 — Source data Fig. 2 [file 44318_2025_405_MOESM6_ESM.zip › Figure 2/2I/2I-Micr.image-Merge, Nucleus, TUNEL, NP (CA-074 Me).tif]

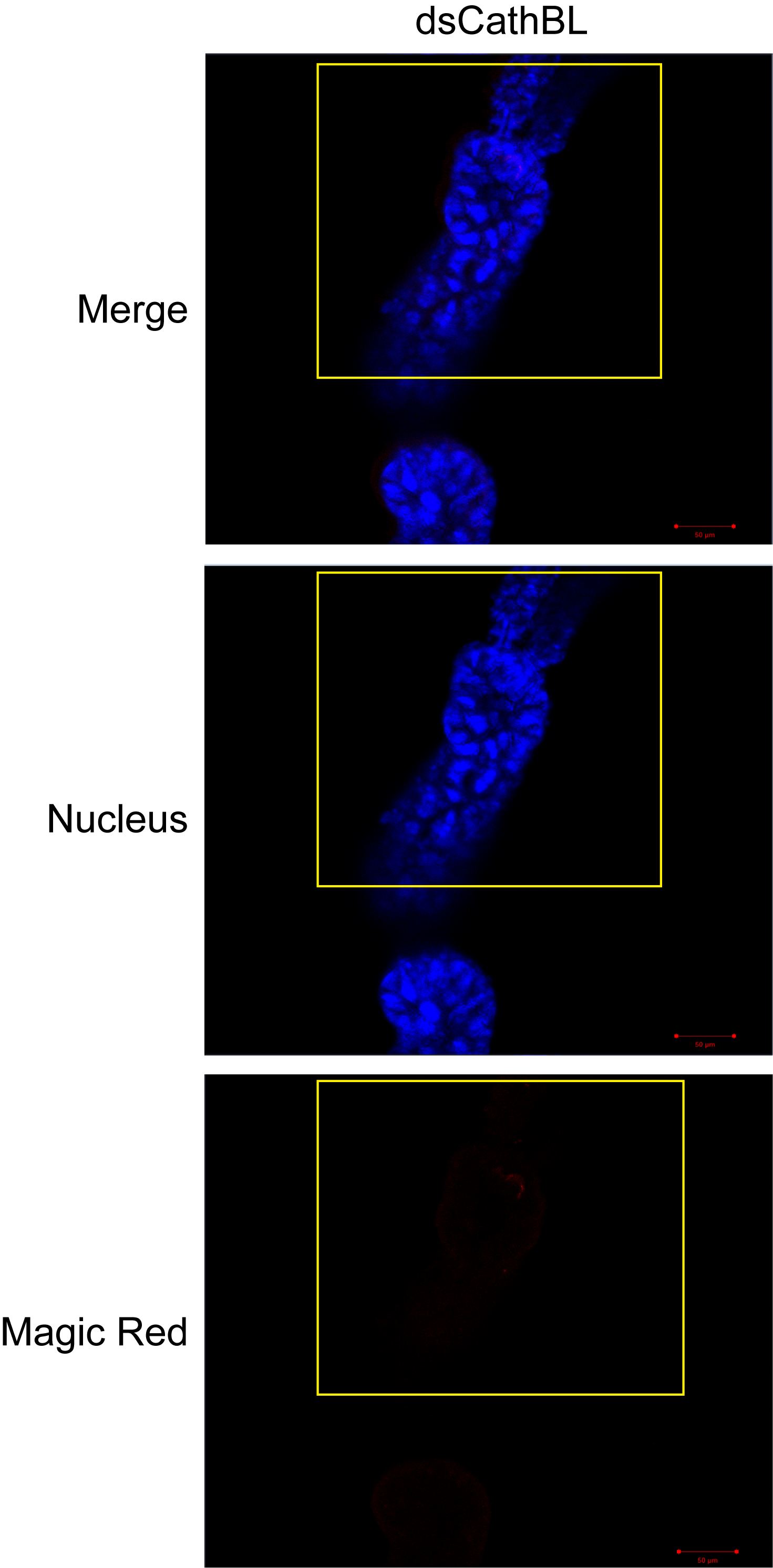

Supplement: Supplementary file 6 — Source data Fig. 2 [file 44318_2025_405_MOESM6_ESM.zip › Figure 2/2H/Micr.image-Merge, Nucleus, Magic Red (dsCathBL).tif]

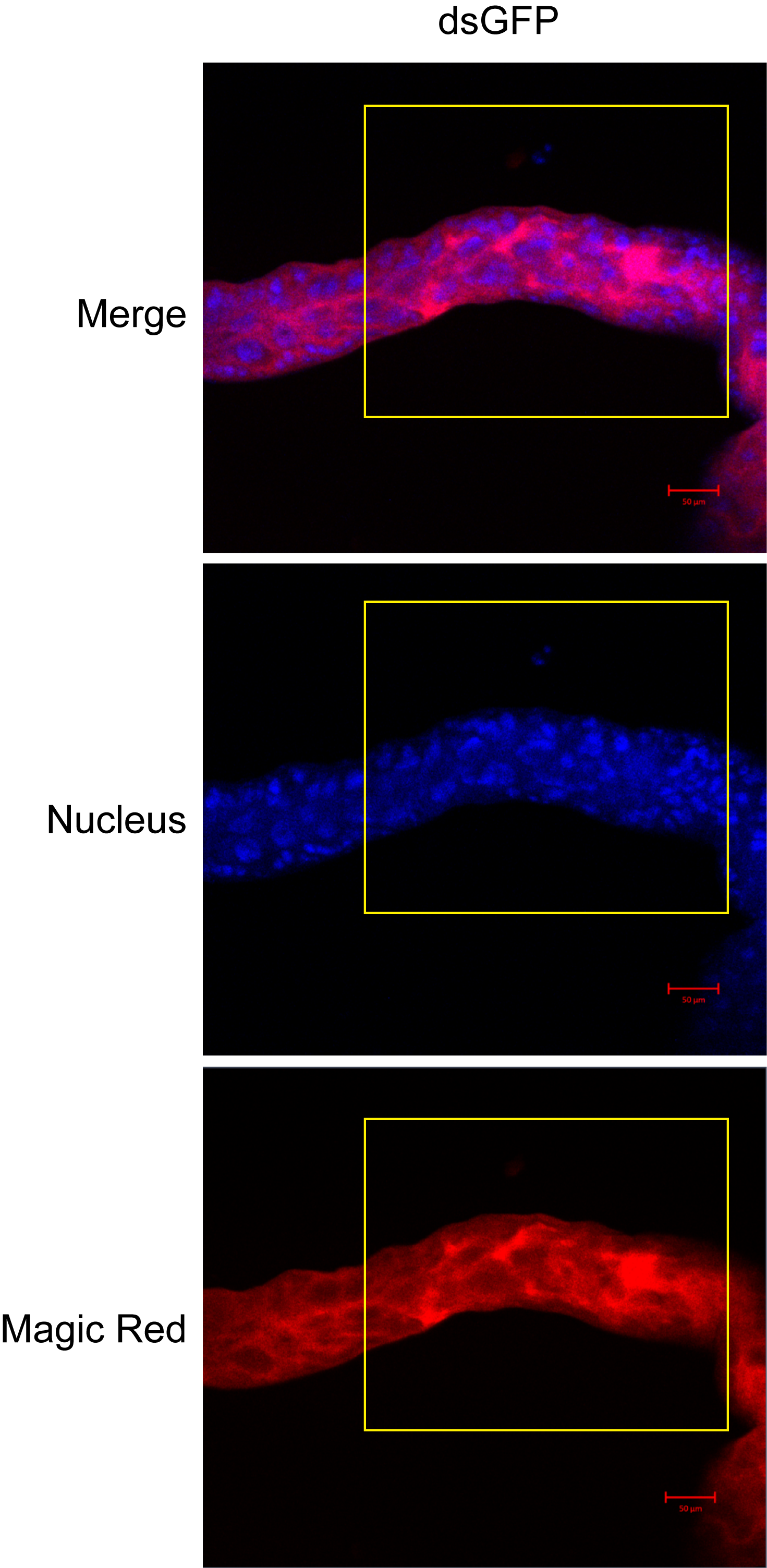

Supplement: Supplementary file 6 — Source data Fig. 2 [file 44318_2025_405_MOESM6_ESM.zip › Figure 2/2H/Micr.image-Merge, Nucleus, Magic Red (dsGFP).tif]

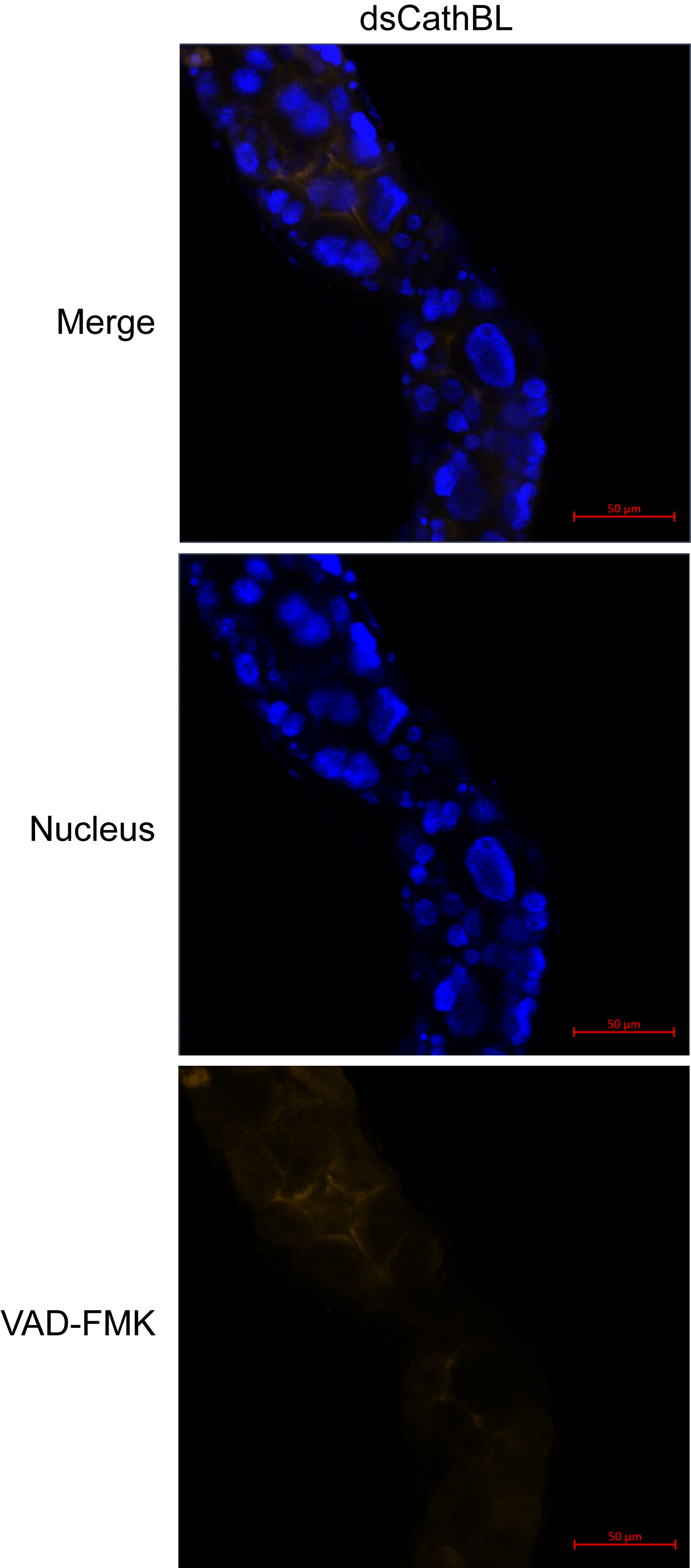

Supplement: Supplementary file 6 — Source data Fig. 2 [file 44318_2025_405_MOESM6_ESM.zip › Figure 2/2K/Micr.image-Merge, Nucleus, VAD-FMK (dsCathBL).tif]

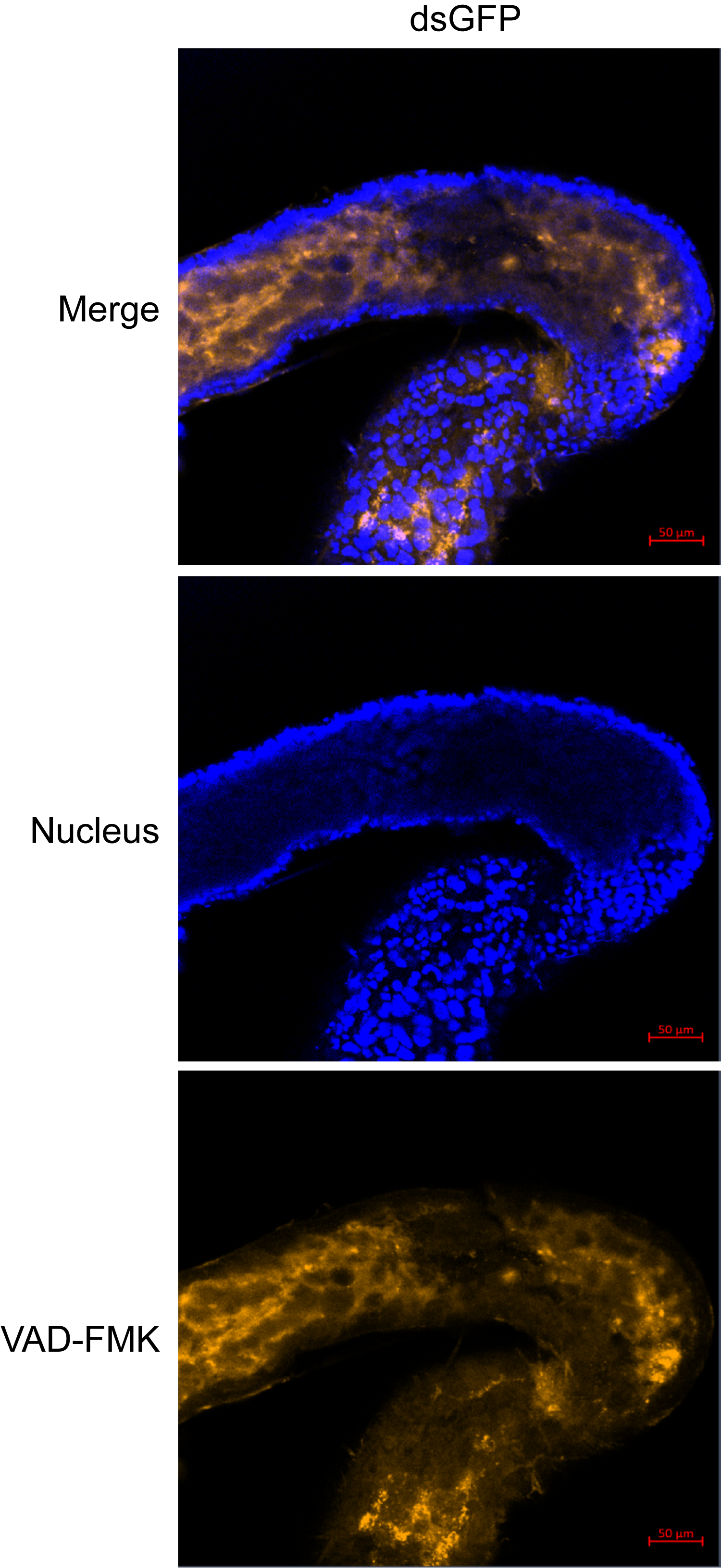

Supplement: Supplementary file 6 — Source data Fig. 2 [file 44318_2025_405_MOESM6_ESM.zip › Figure 2/2K/Micr.image-Merge, Nucleus, VAD-FMK (dsGFP).tif]

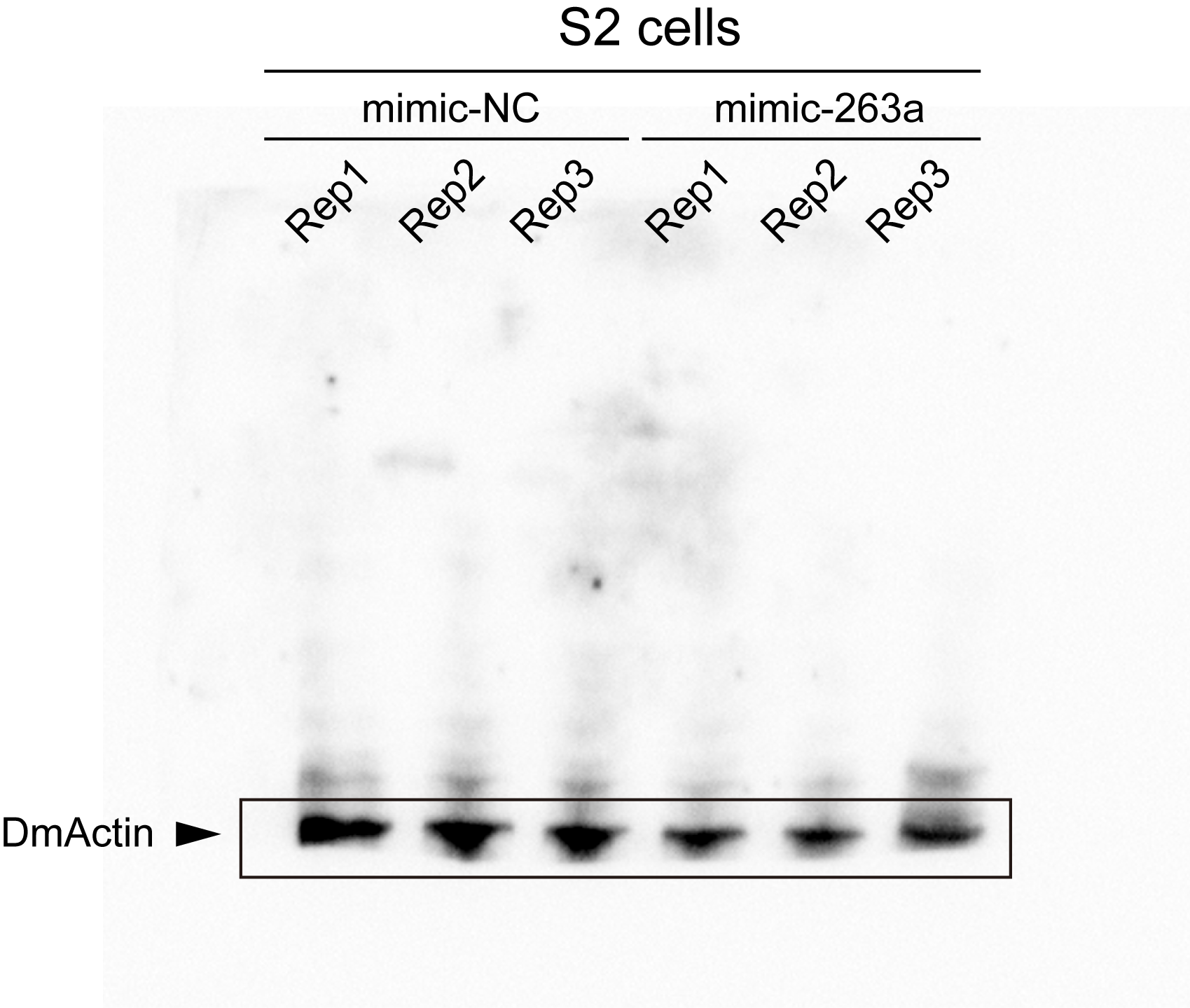

Supplement: Supplementary file 6 — Source data Fig. 2 [file 44318_2025_405_MOESM6_ESM.zip › Figure 2/2E/Northern blot DmActin (bottom).tif]

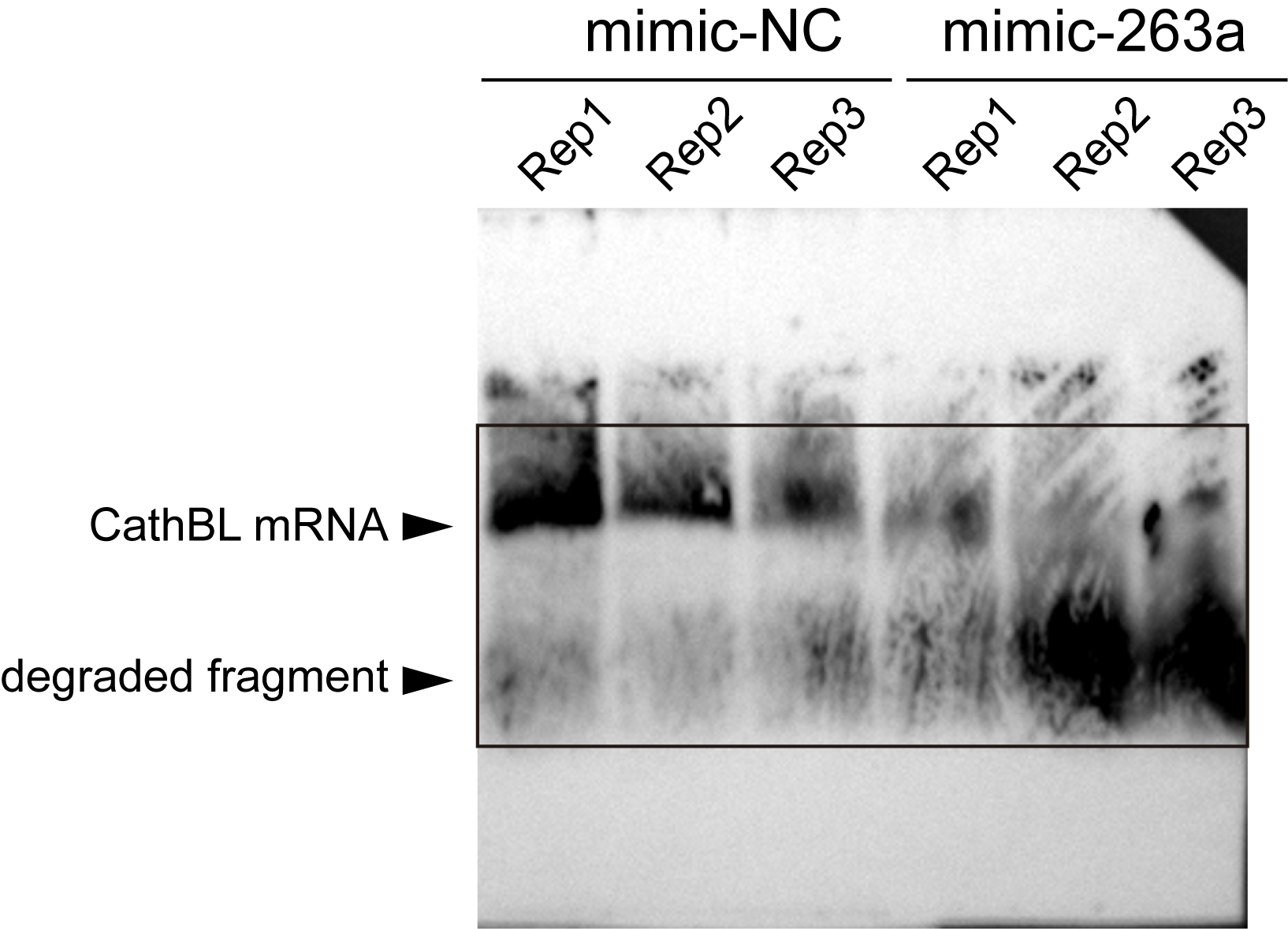

Supplement: Supplementary file 6 — Source data Fig. 2 [file 44318_2025_405_MOESM6_ESM.zip › Figure 2/2E/Northern blot CathBL-mRNA (top).tif]

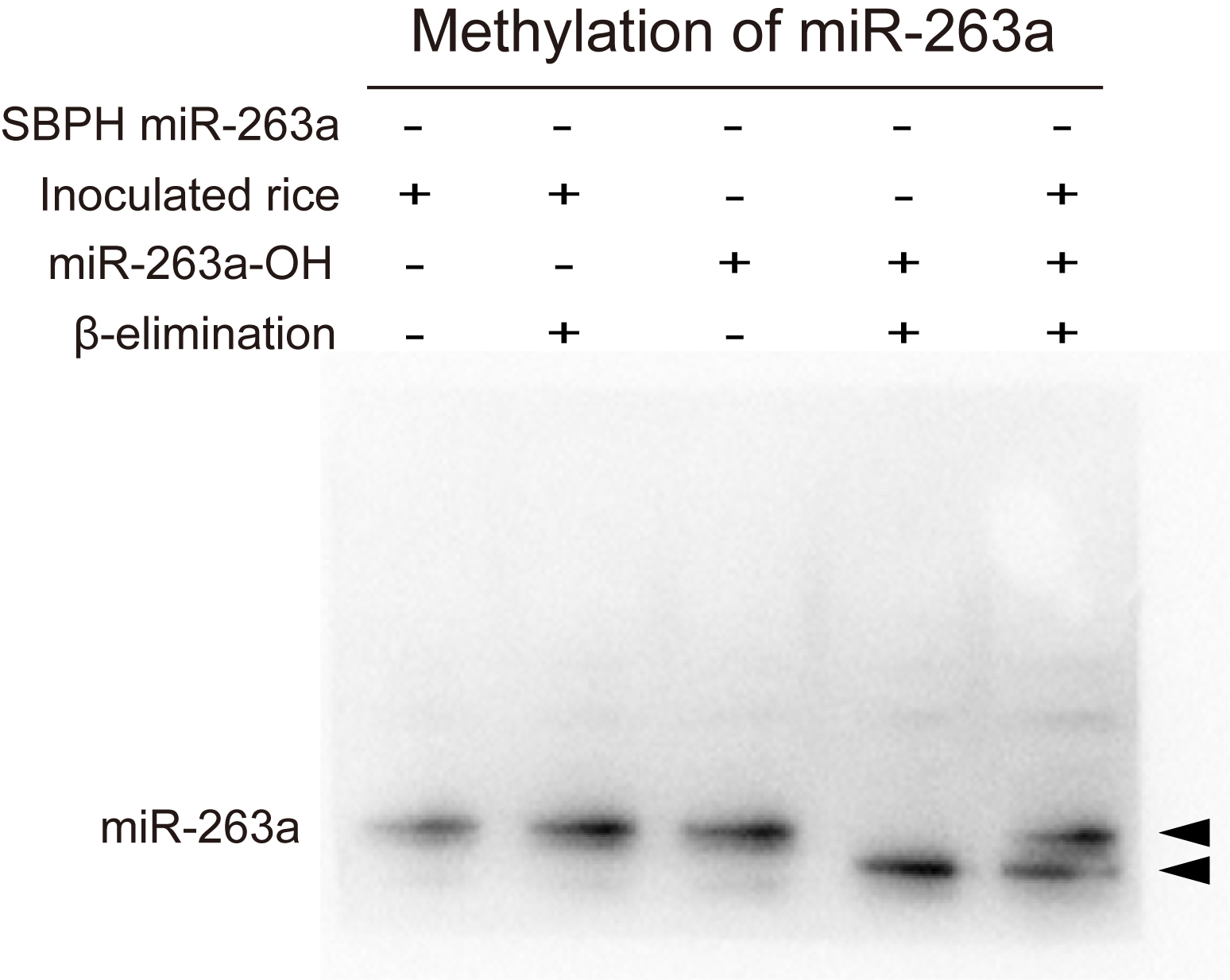

Supplement: Supplementary file 7 — Source data Fig. 3 [file 44318_2025_405_MOESM7_ESM.zip › Figure 3/3E/Northern miR-263a (top left).tif]

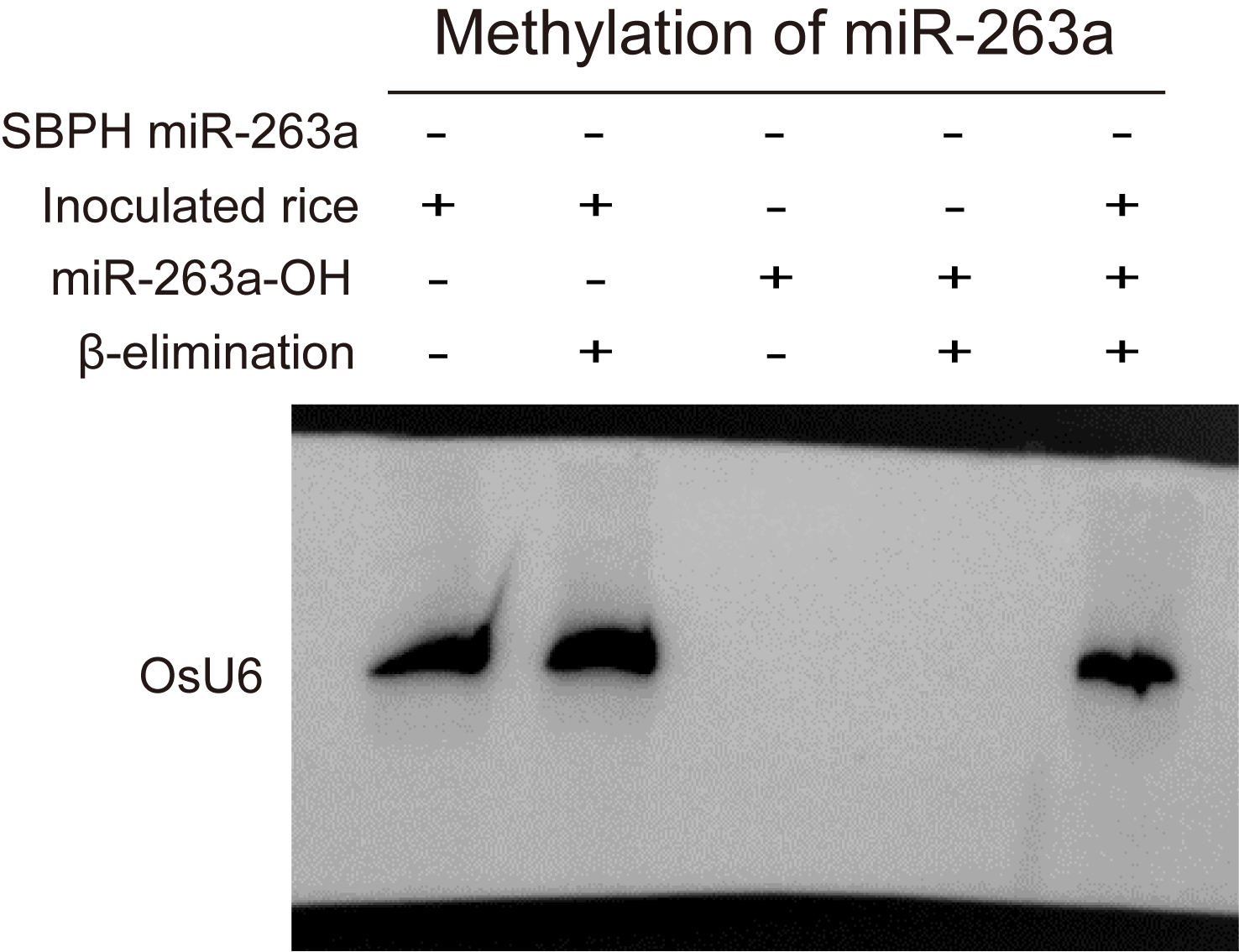

Supplement: Supplementary file 7 — Source data Fig. 3 [file 44318_2025_405_MOESM7_ESM.zip › Figure 3/3E/Northern OsU6 (bottom left).tif]

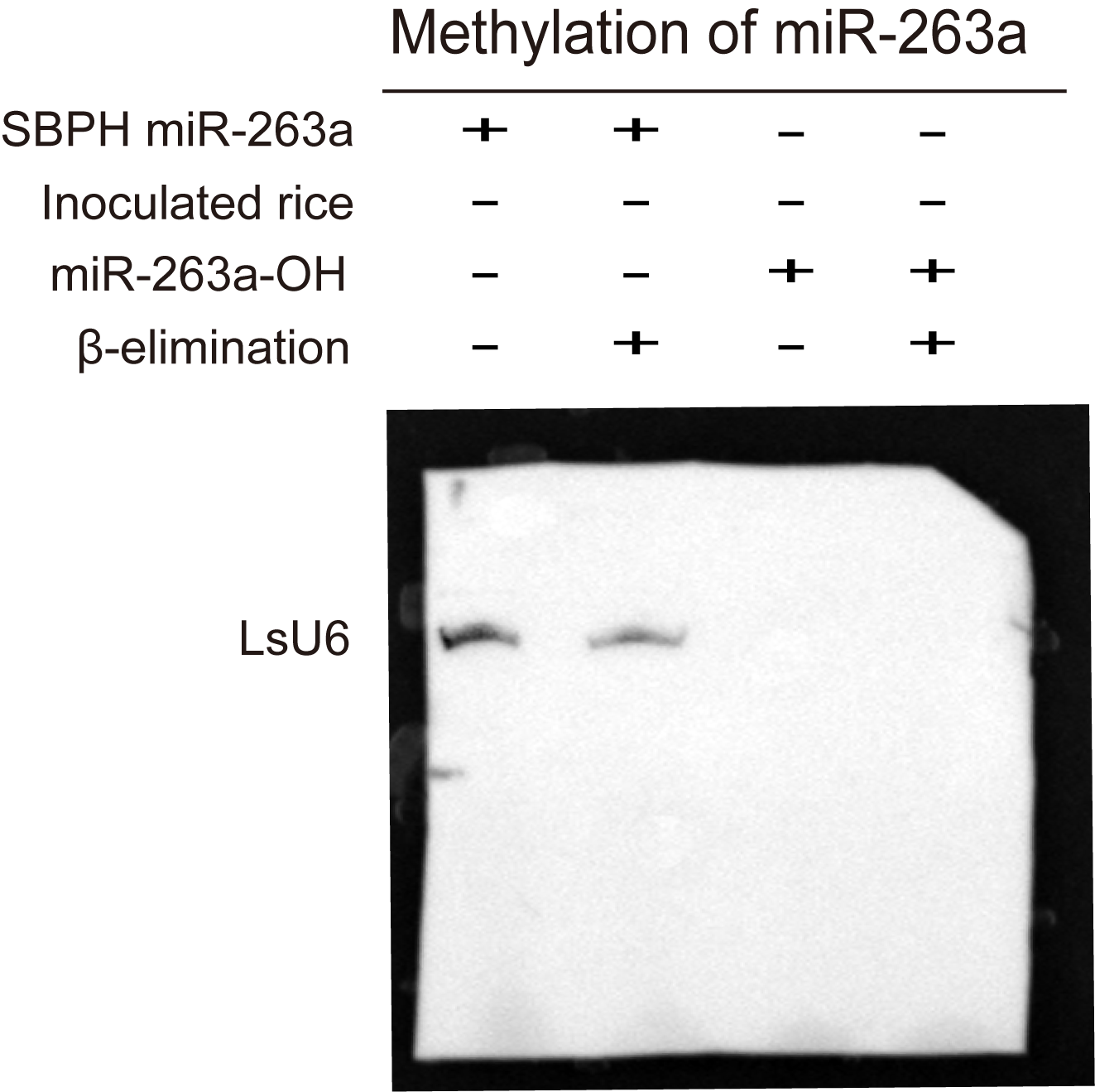

Supplement: Supplementary file 7 — Source data Fig. 3 [file 44318_2025_405_MOESM7_ESM.zip › Figure 3/3E/Northern LsU6 (bottom right).tif]

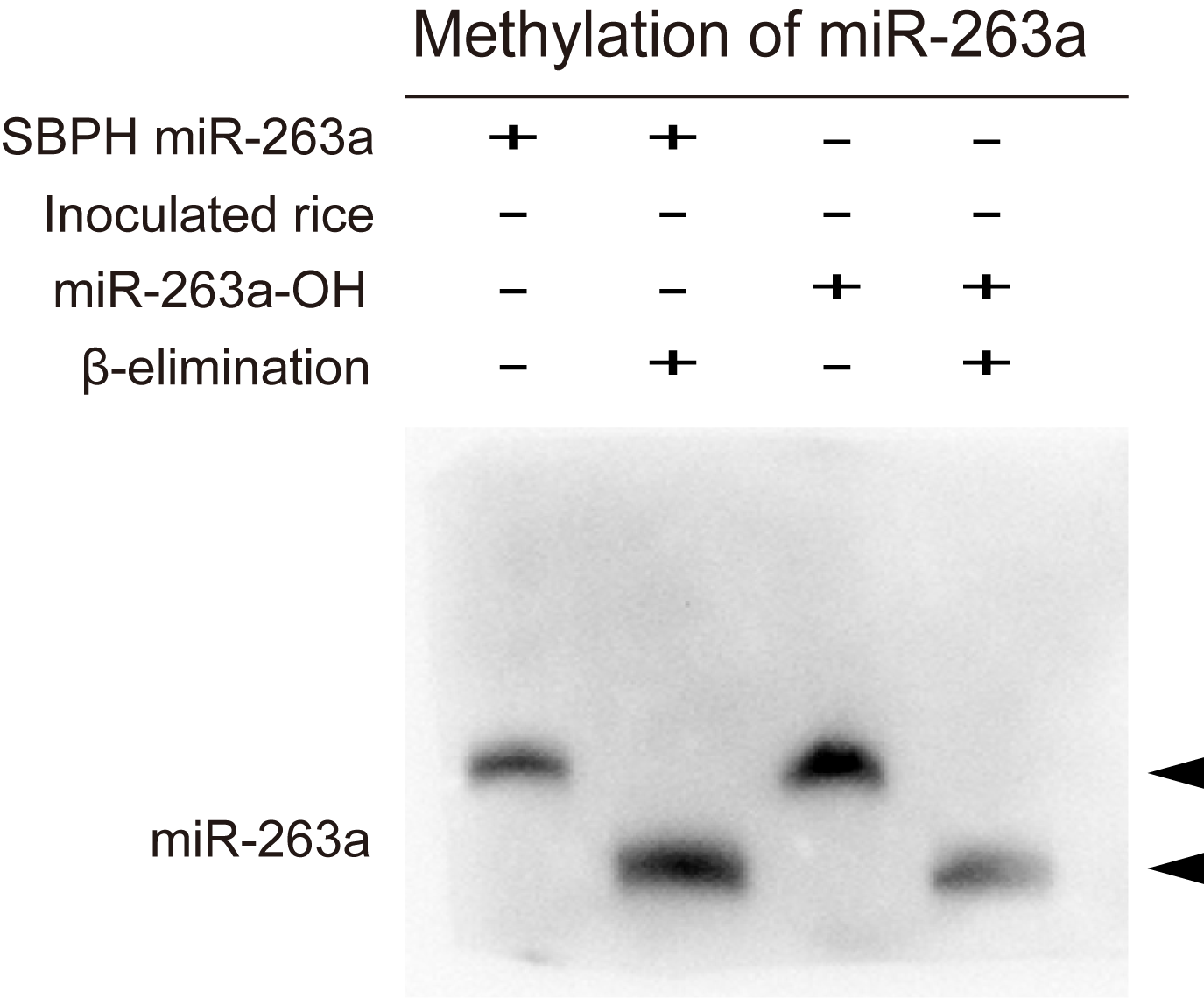

Supplement: Supplementary file 7 — Source data Fig. 3 [file 44318_2025_405_MOESM7_ESM.zip › Figure 3/3E/Northern miR-263a (top right).tif]

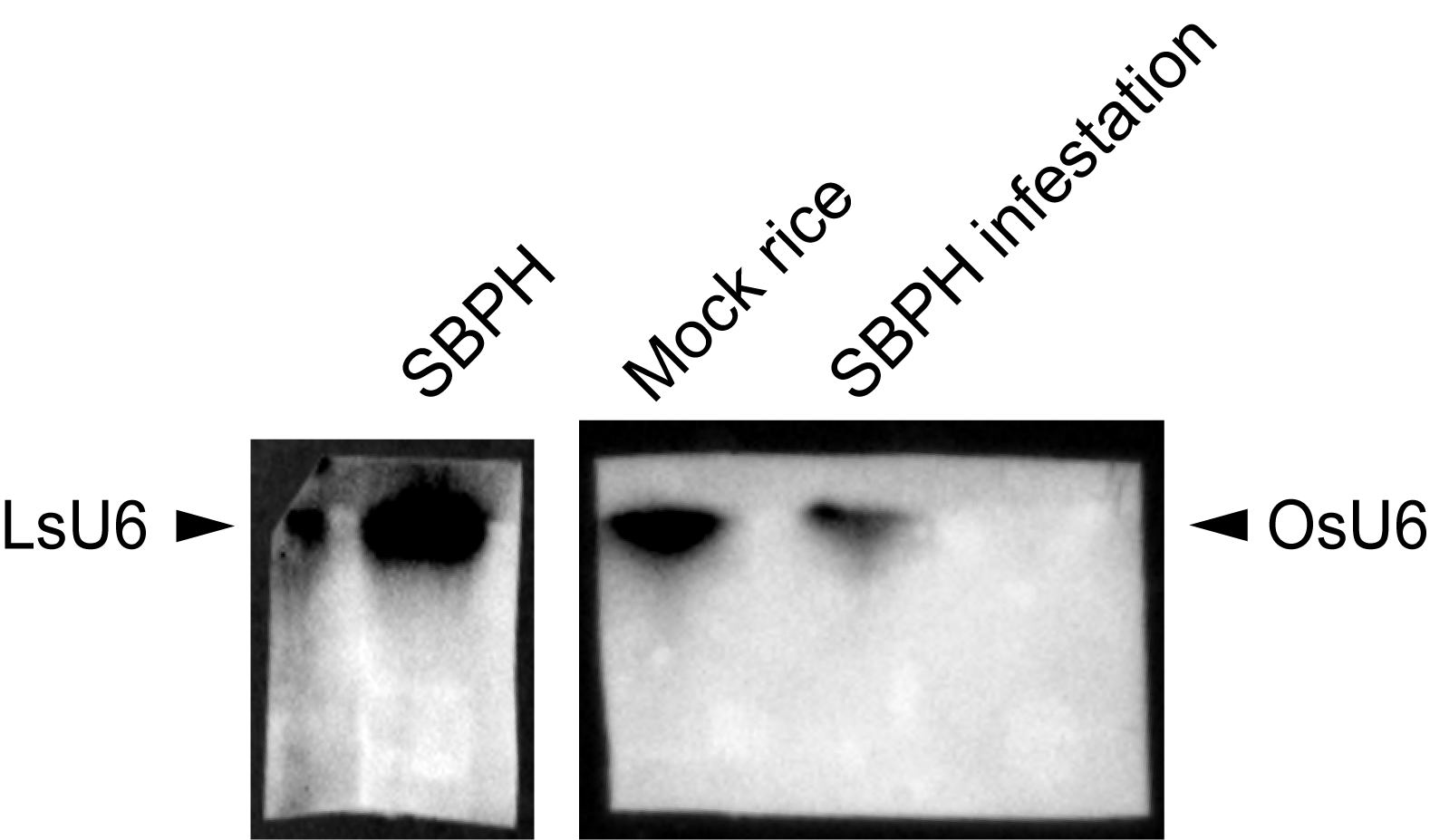

Supplement: Supplementary file 7 — Source data Fig. 3 [file 44318_2025_405_MOESM7_ESM.zip › Figure 3/3A/Northern blot-U6 (middle).tif]

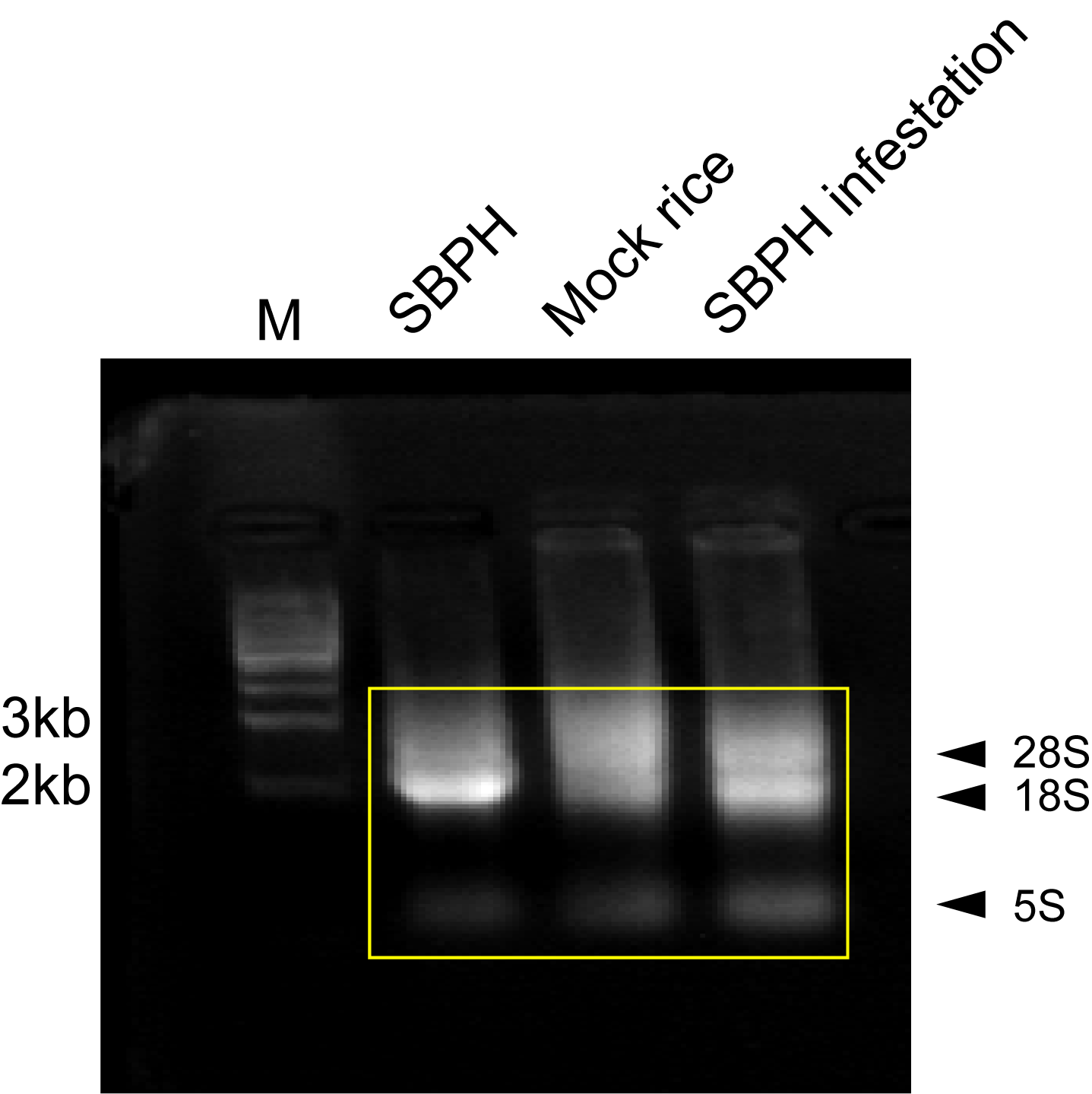

Supplement: Supplementary file 7 — Source data Fig. 3 [file 44318_2025_405_MOESM7_ESM.zip › Figure 3/3A/Gel-rRNA (bottom).tif]

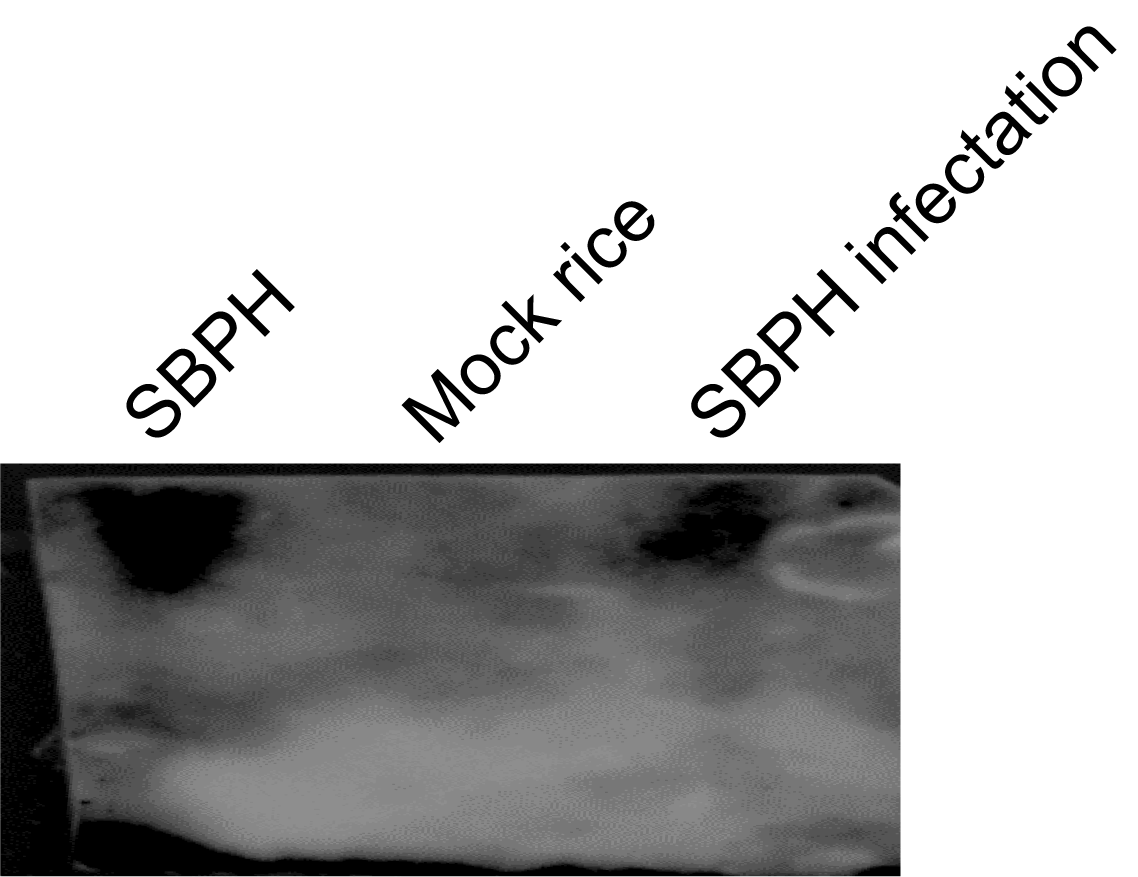

Supplement: Supplementary file 7 — Source data Fig. 3 [file 44318_2025_405_MOESM7_ESM.zip › Figure 3/3A/Northern blot-miR-263a (top).tif]

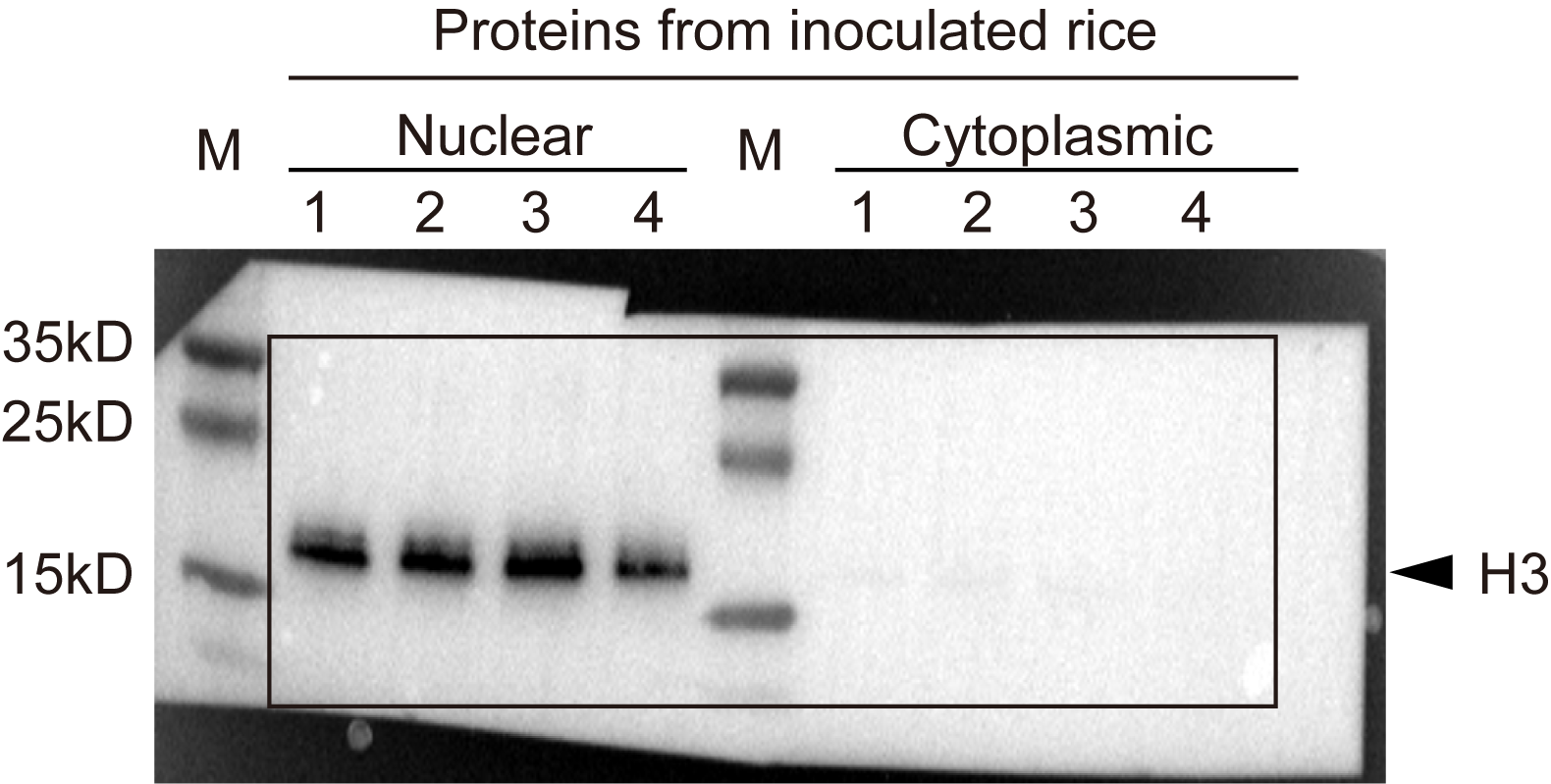

Supplement: Supplementary file 7 — Source data Fig. 3 [file 44318_2025_405_MOESM7_ESM.zip › Figure 3/3F/Western blot-H3 (top).tif]

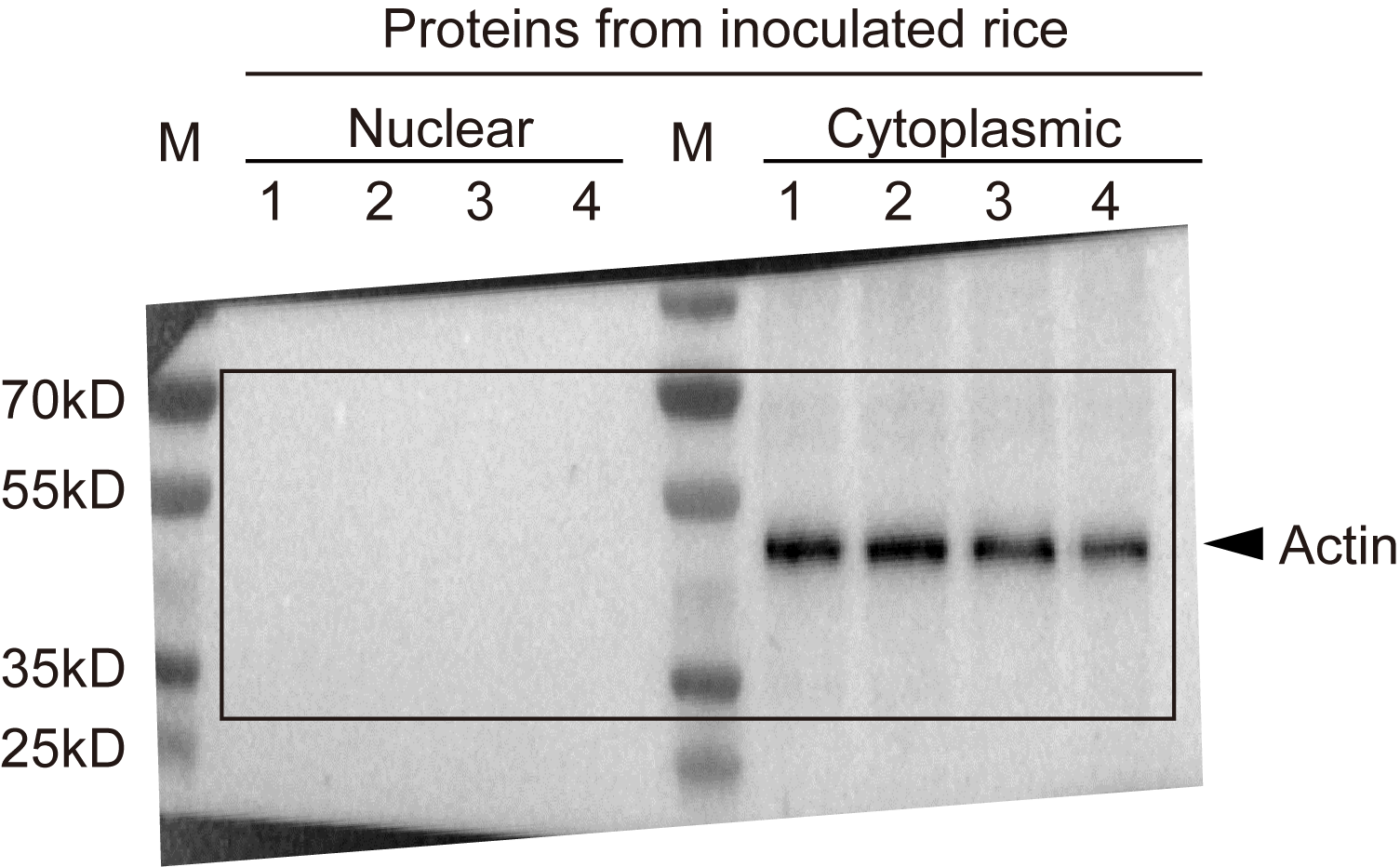

Supplement: Supplementary file 7 — Source data Fig. 3 [file 44318_2025_405_MOESM7_ESM.zip › Figure 3/3F/Western blot-Actin (bottom).tif]

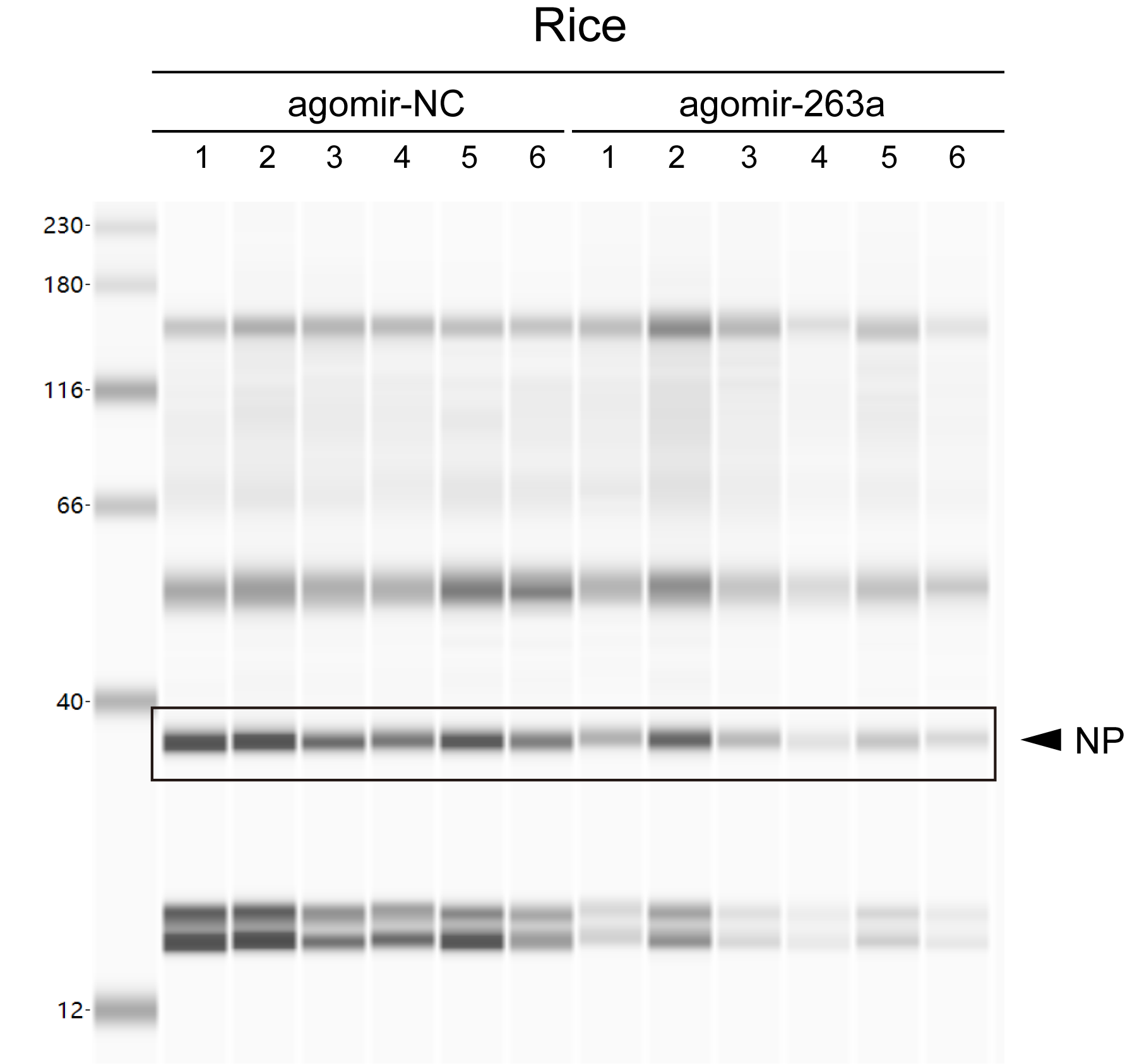

Supplement: Supplementary file 8 — Source data Fig. 4 [file 44318_2025_405_MOESM8_ESM.zip › Figure 4/4B/Western NP-Rice (top).tif]

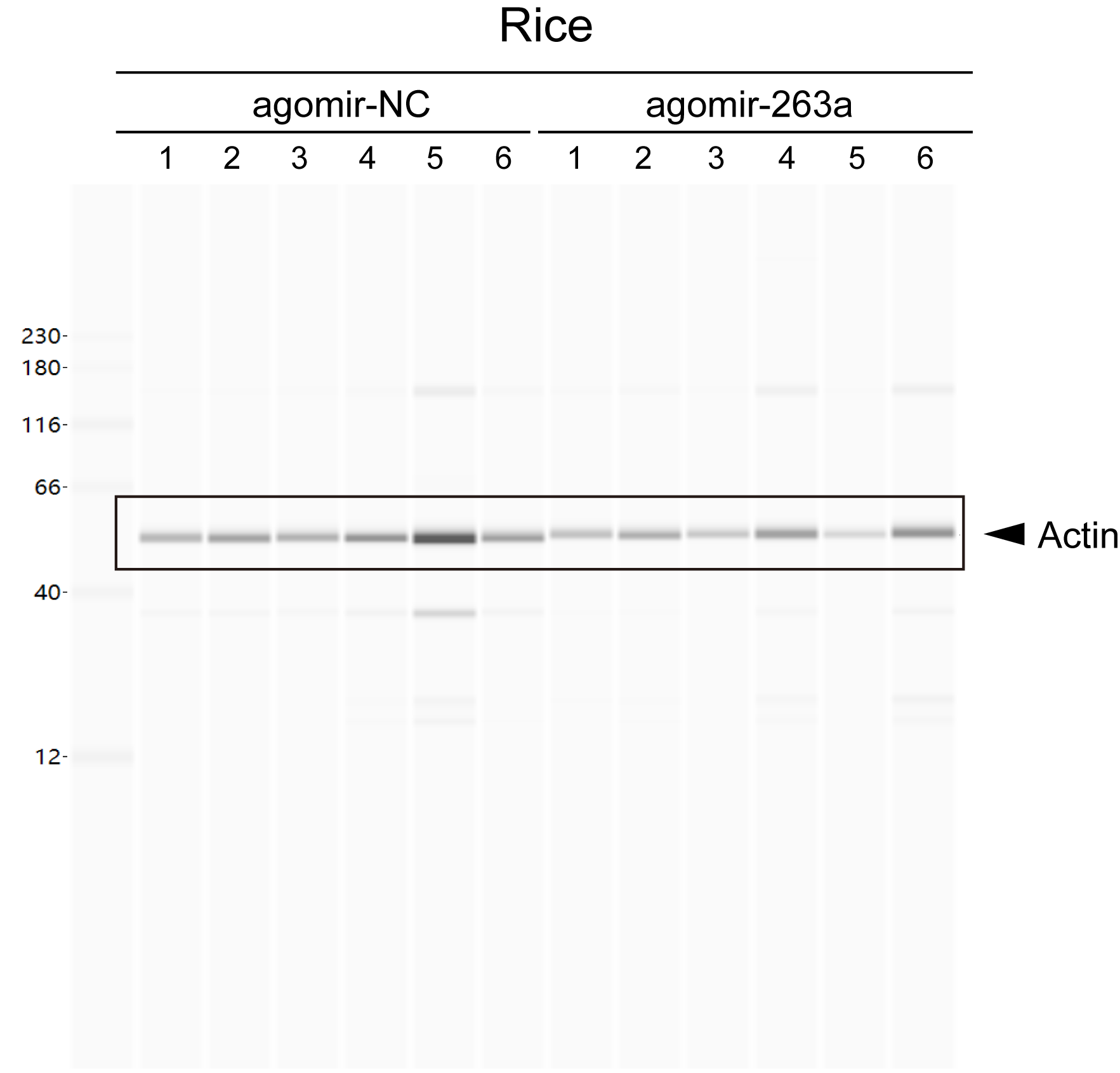

Supplement: Supplementary file 8 — Source data Fig. 4 [file 44318_2025_405_MOESM8_ESM.zip › Figure 4/4B/Western Actin-Rice (bottom).tif]

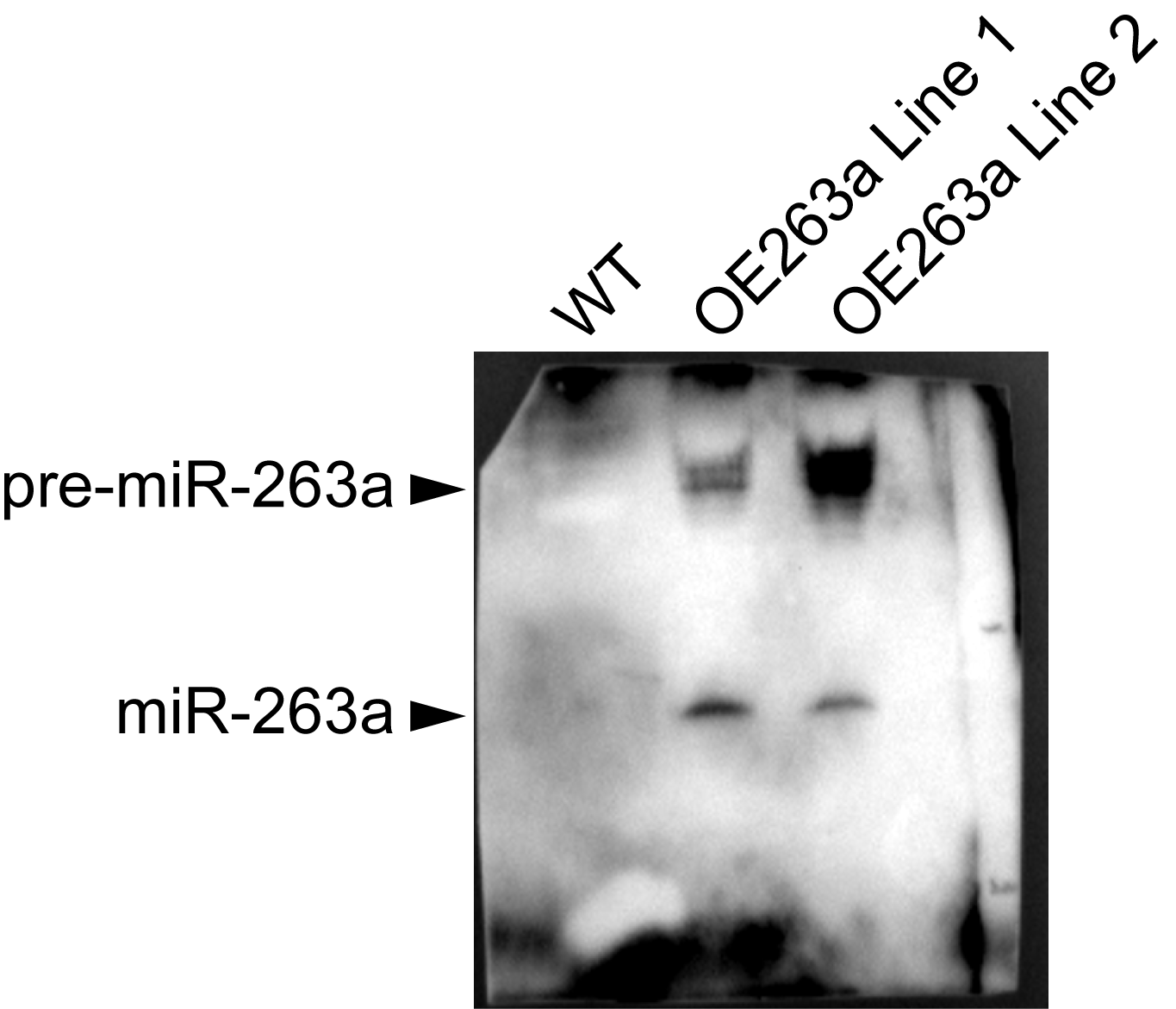

Supplement: Supplementary file 8 — Source data Fig. 4 [file 44318_2025_405_MOESM8_ESM.zip › Figure 4/4F/Northern miR-263a-WT, OE263a (top).tif]

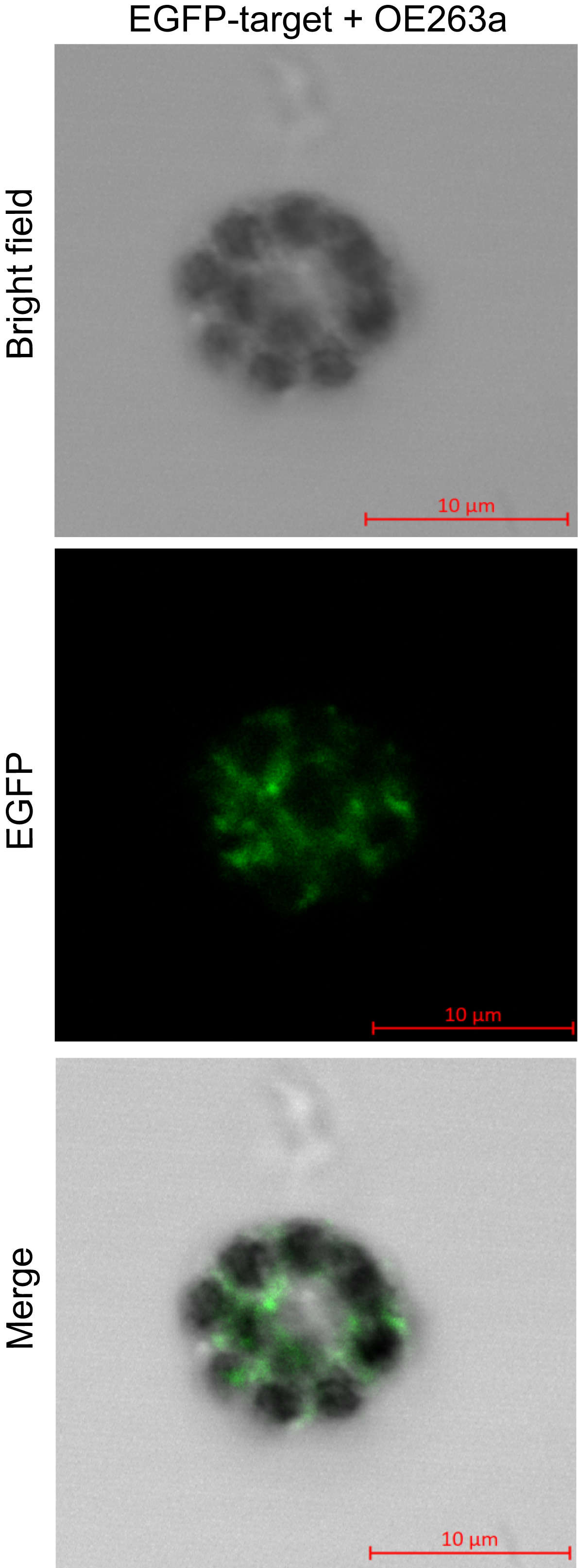

Supplement: Supplementary file 9 — Source data Fig. 5 [file 44318_2025_405_MOESM9_ESM.zip › Figure 5/5C/Micr. image-EGFP-target OE263a (bottom).tif]

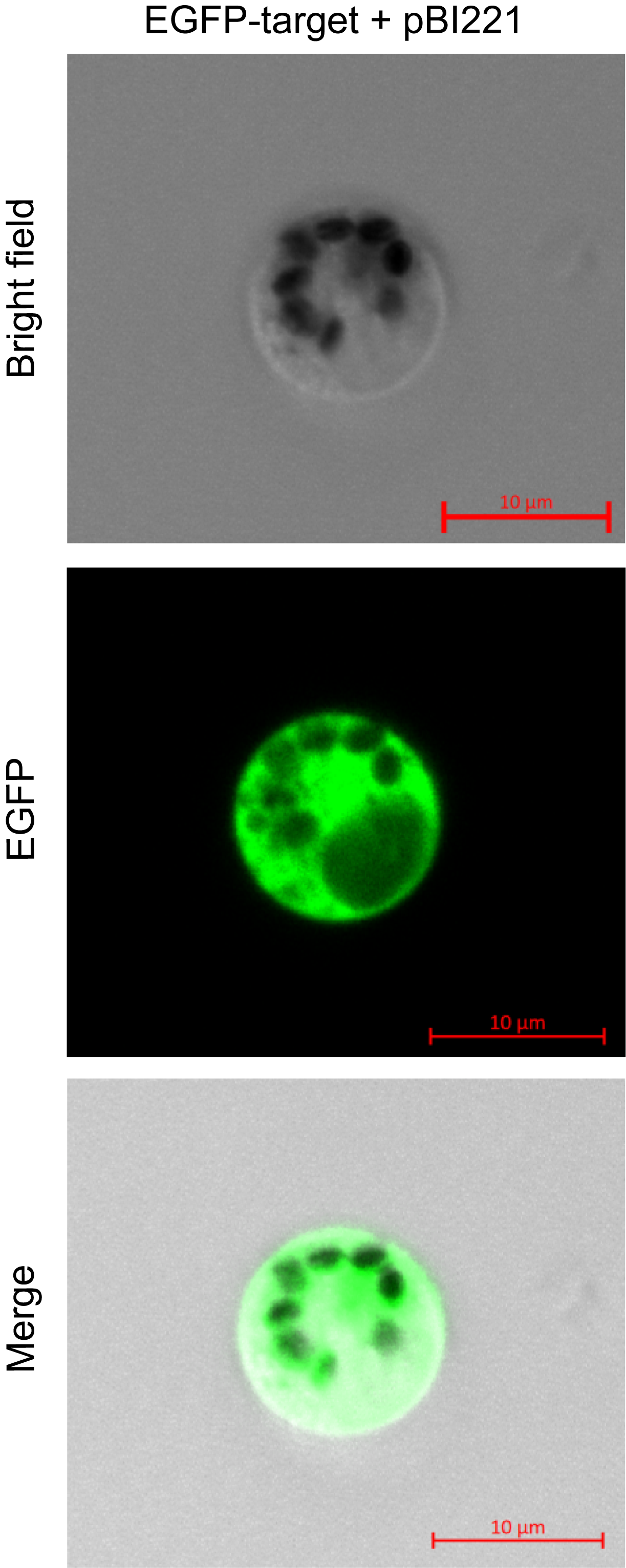

Supplement: Supplementary file 9 — Source data Fig. 5 [file 44318_2025_405_MOESM9_ESM.zip › Figure 5/5C/Micr. image-EGFP-target pBI221 (top).tif]

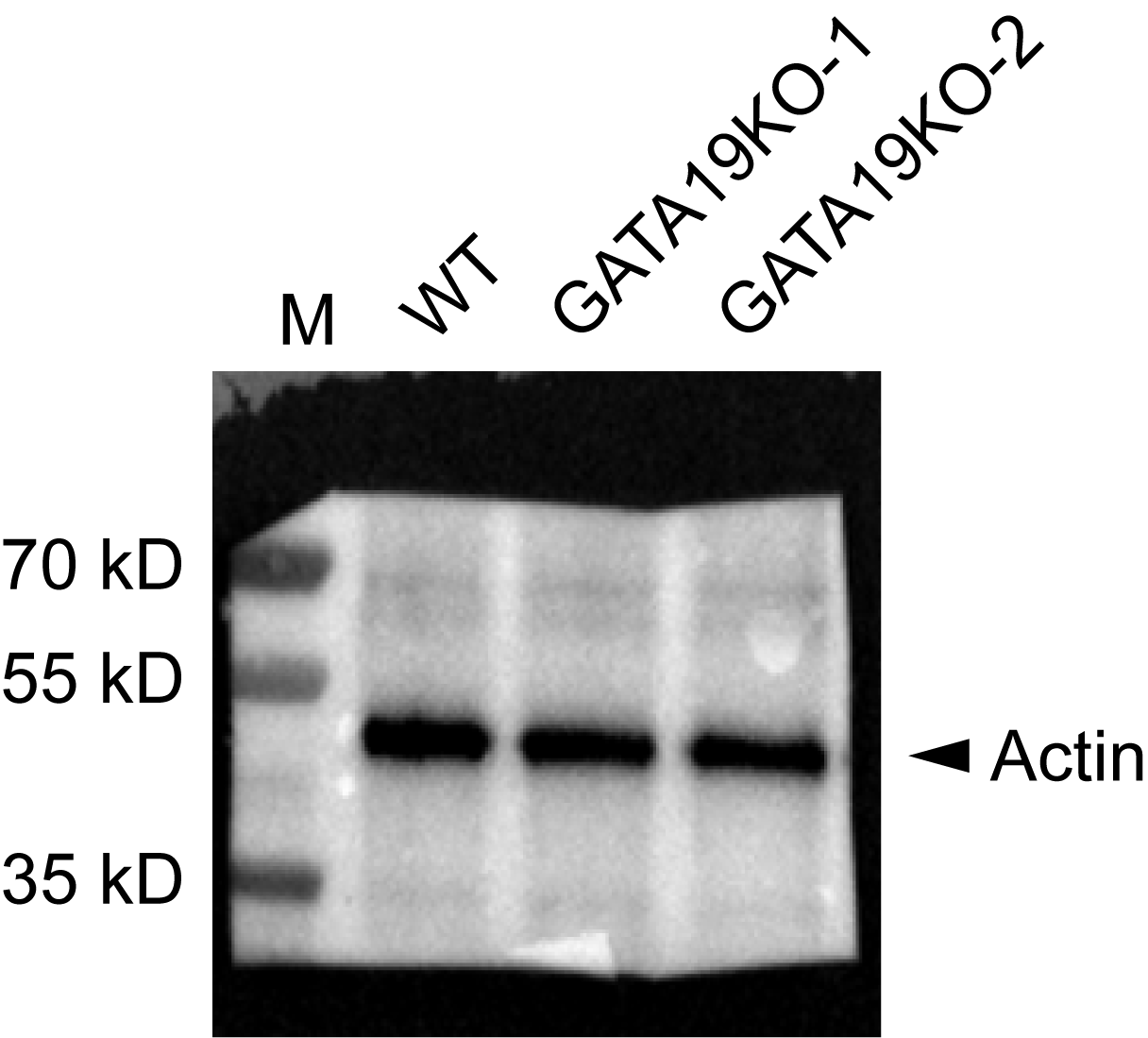

Supplement: Supplementary file 9 — Source data Fig. 5 [file 44318_2025_405_MOESM9_ESM.zip › Figure 5/5E/Western blot-Actin (bottom).tif]

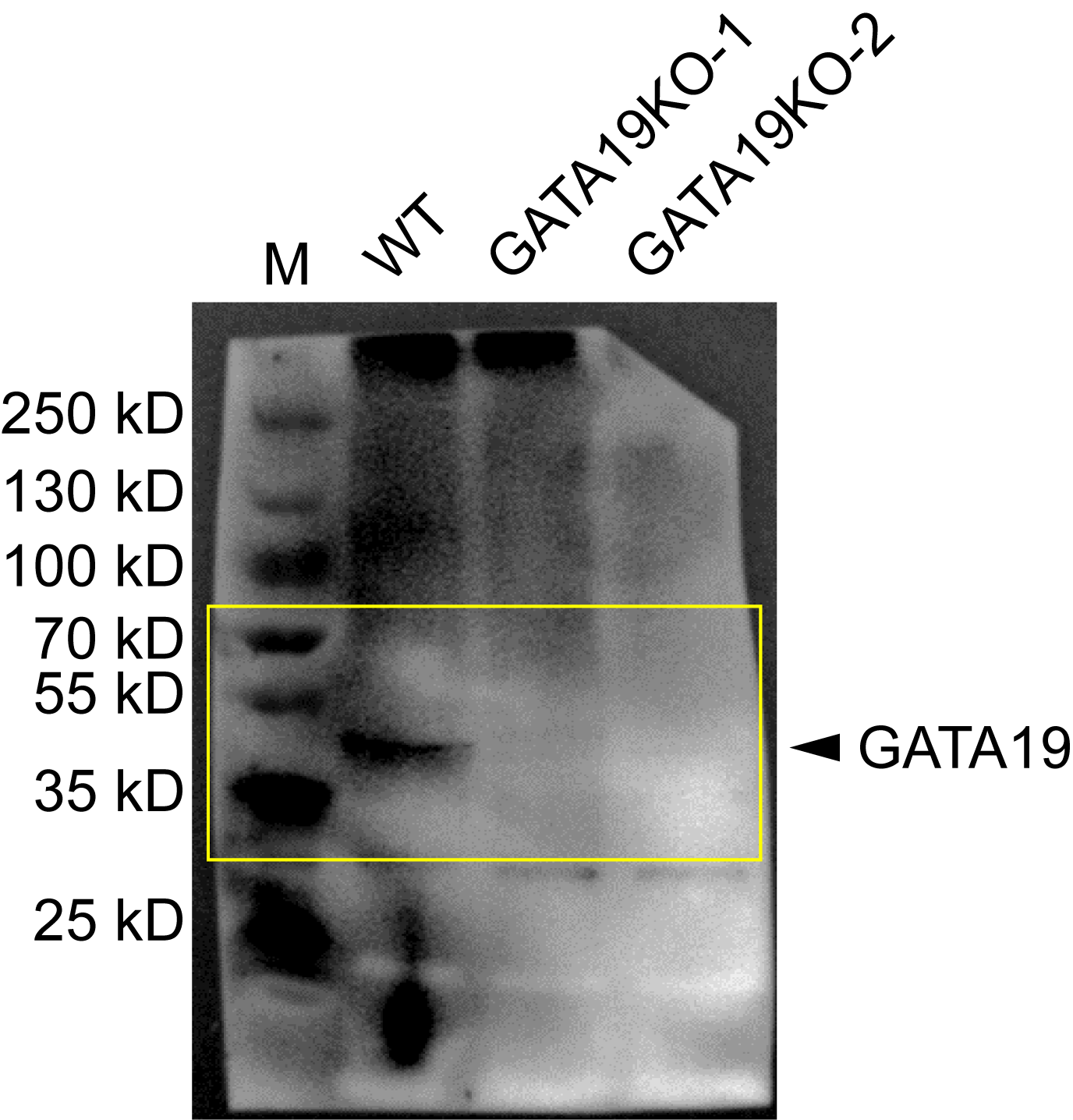

Supplement: Supplementary file 9 — Source data Fig. 5 [file 44318_2025_405_MOESM9_ESM.zip › Figure 5/5E/Western blot-GATA19 (top).tif]

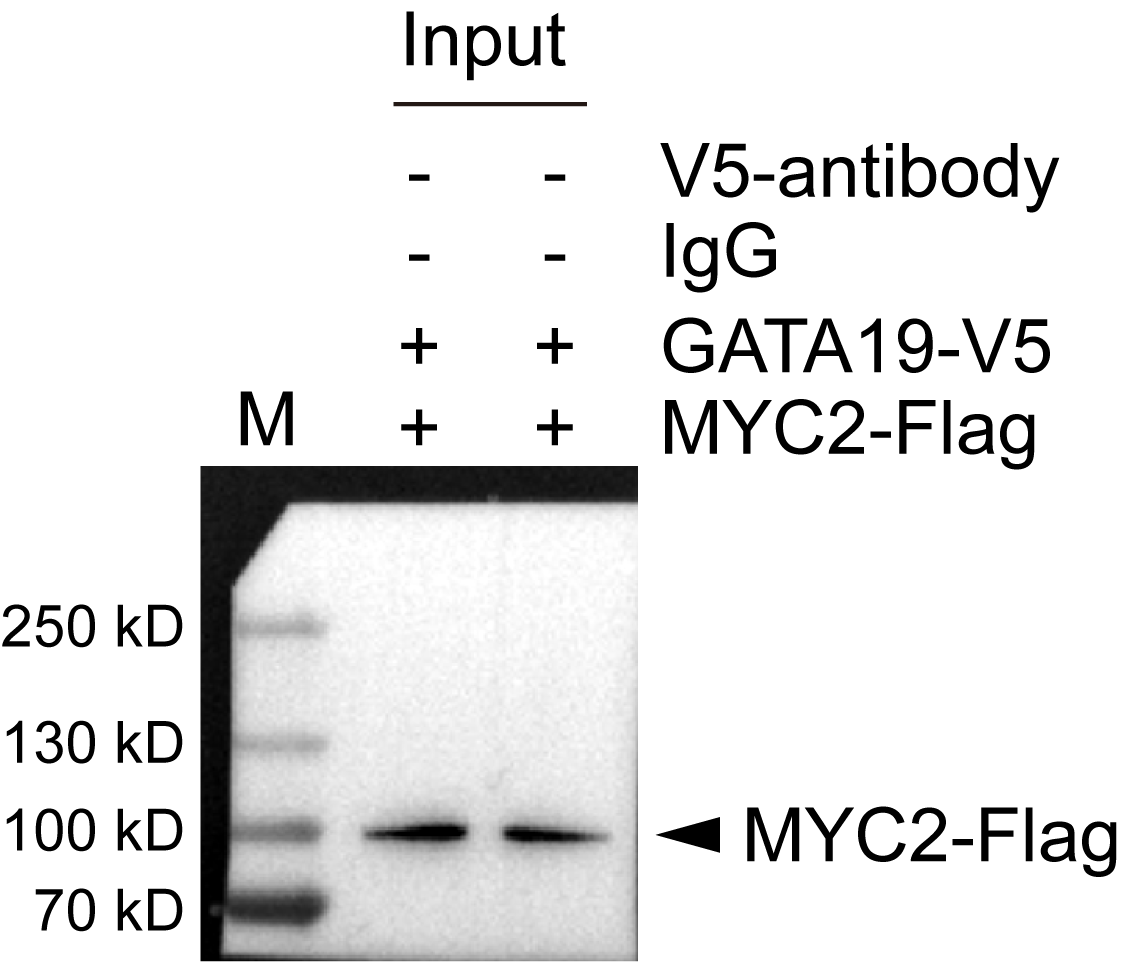

Supplement: Supplementary file 10 — Source data Fig. 6 [file 44318_2025_405_MOESM10_ESM.zip › Figure 6/6F/Input-MYC2Flag (bottom left).tif]

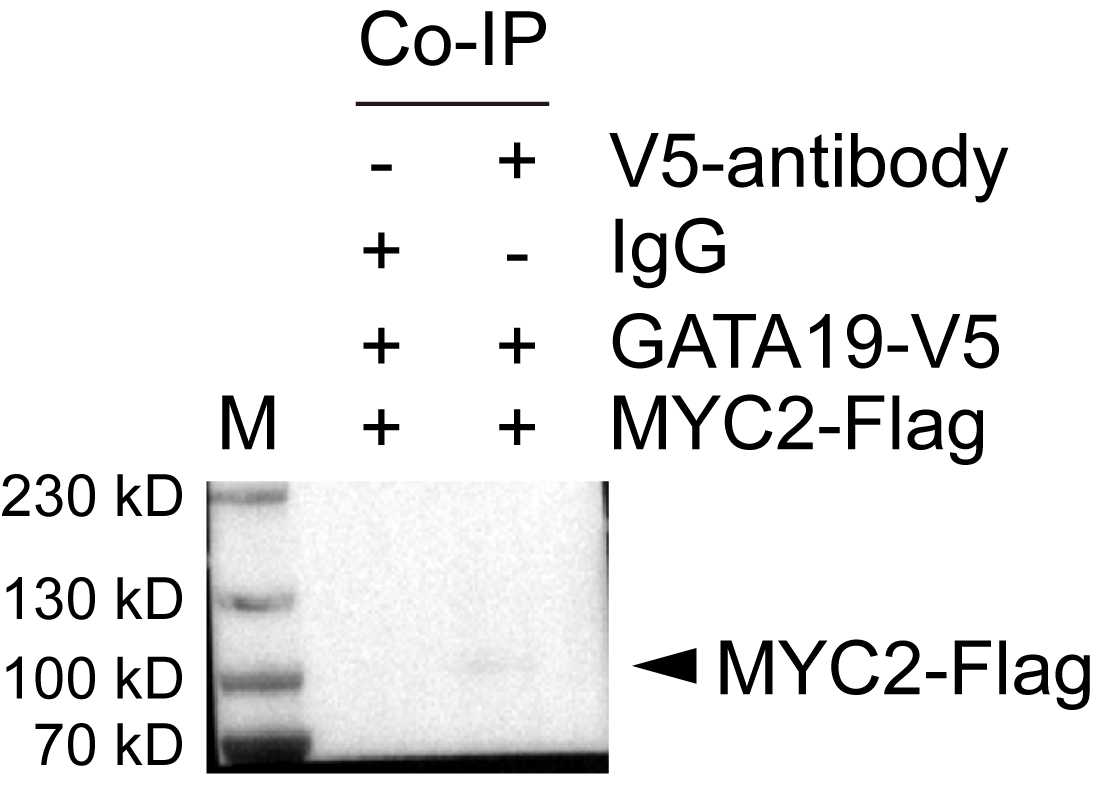

Supplement: Supplementary file 10 — Source data Fig. 6 [file 44318_2025_405_MOESM10_ESM.zip › Figure 6/6F/CoIP-MYC2Flag (bottom right).tif]

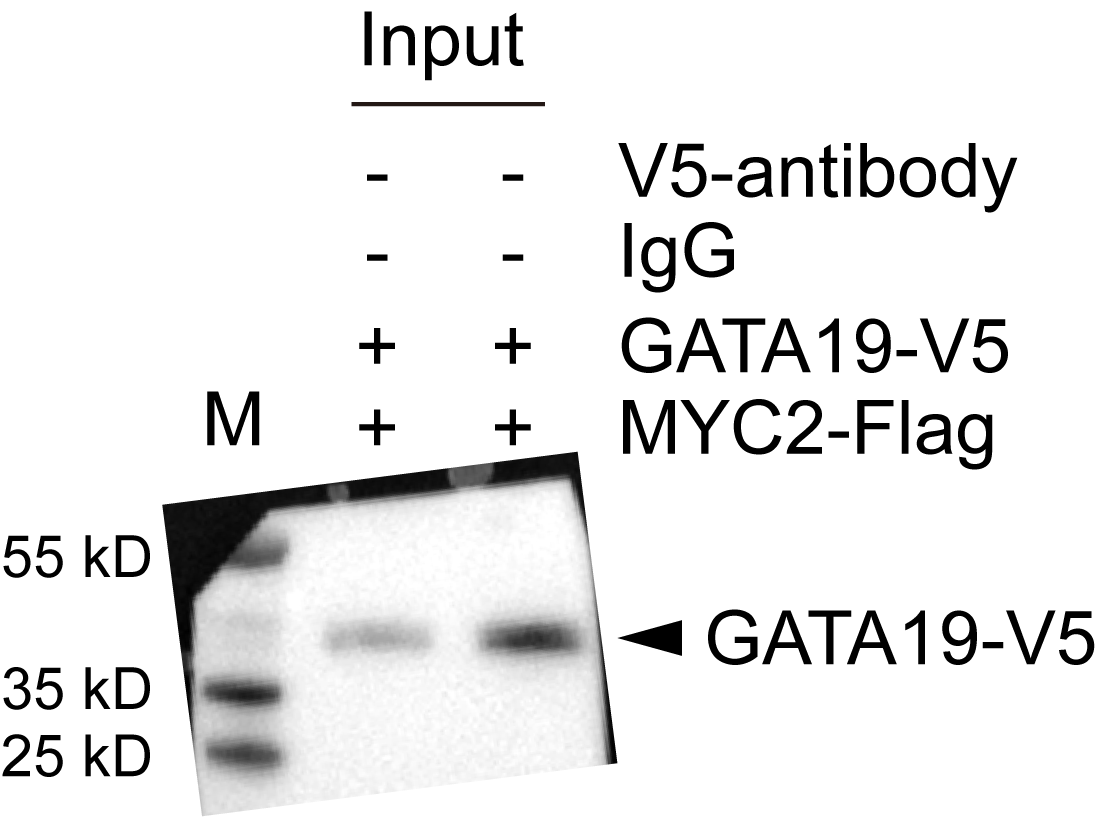

Supplement: Supplementary file 10 — Source data Fig. 6 [file 44318_2025_405_MOESM10_ESM.zip › Figure 6/6F/Input-GATA19V5 (top left).tif]

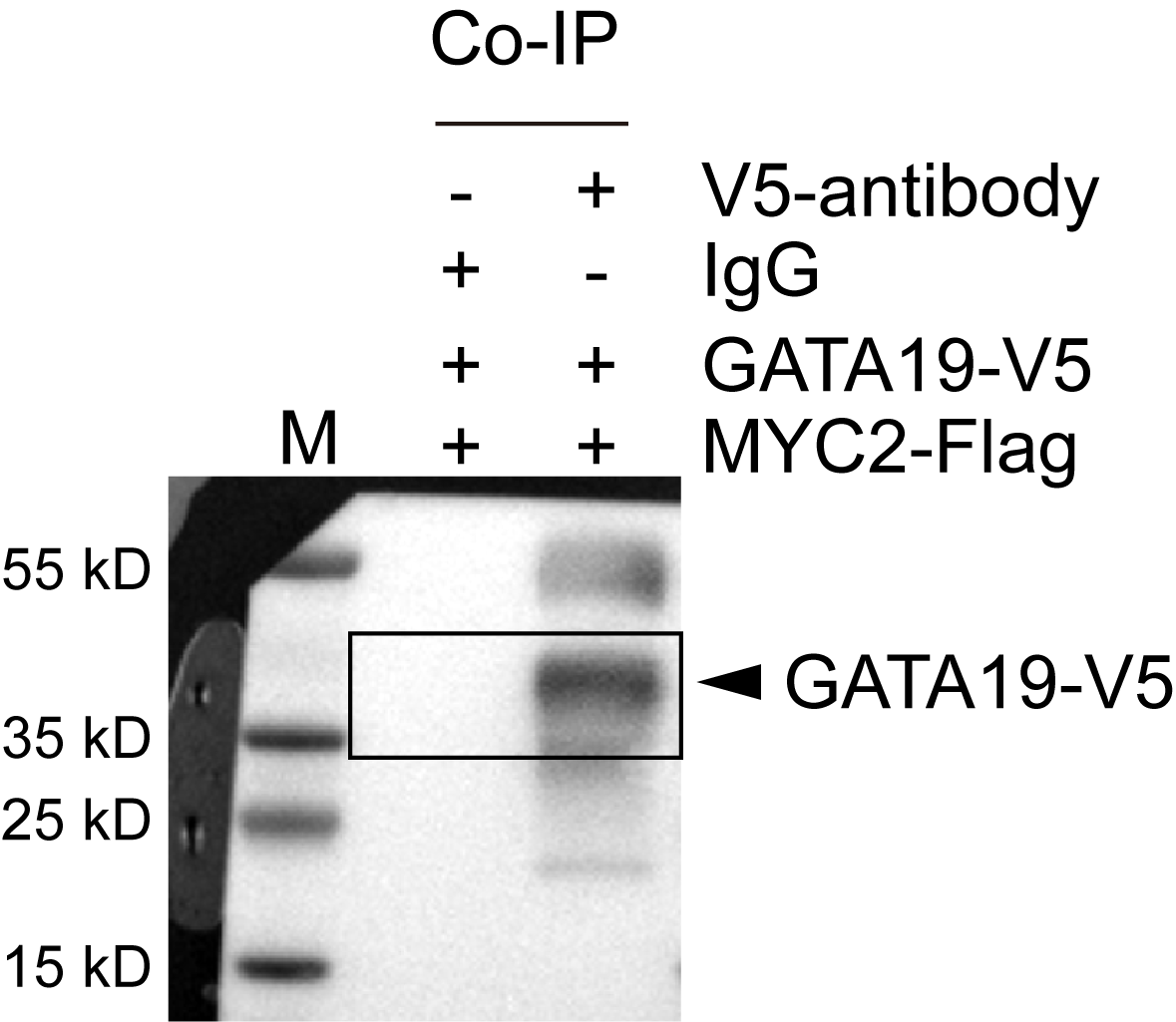

Supplement: Supplementary file 10 — Source data Fig. 6 [file 44318_2025_405_MOESM10_ESM.zip › Figure 6/6F/CoIP-GATA19V5 (top right).tif]

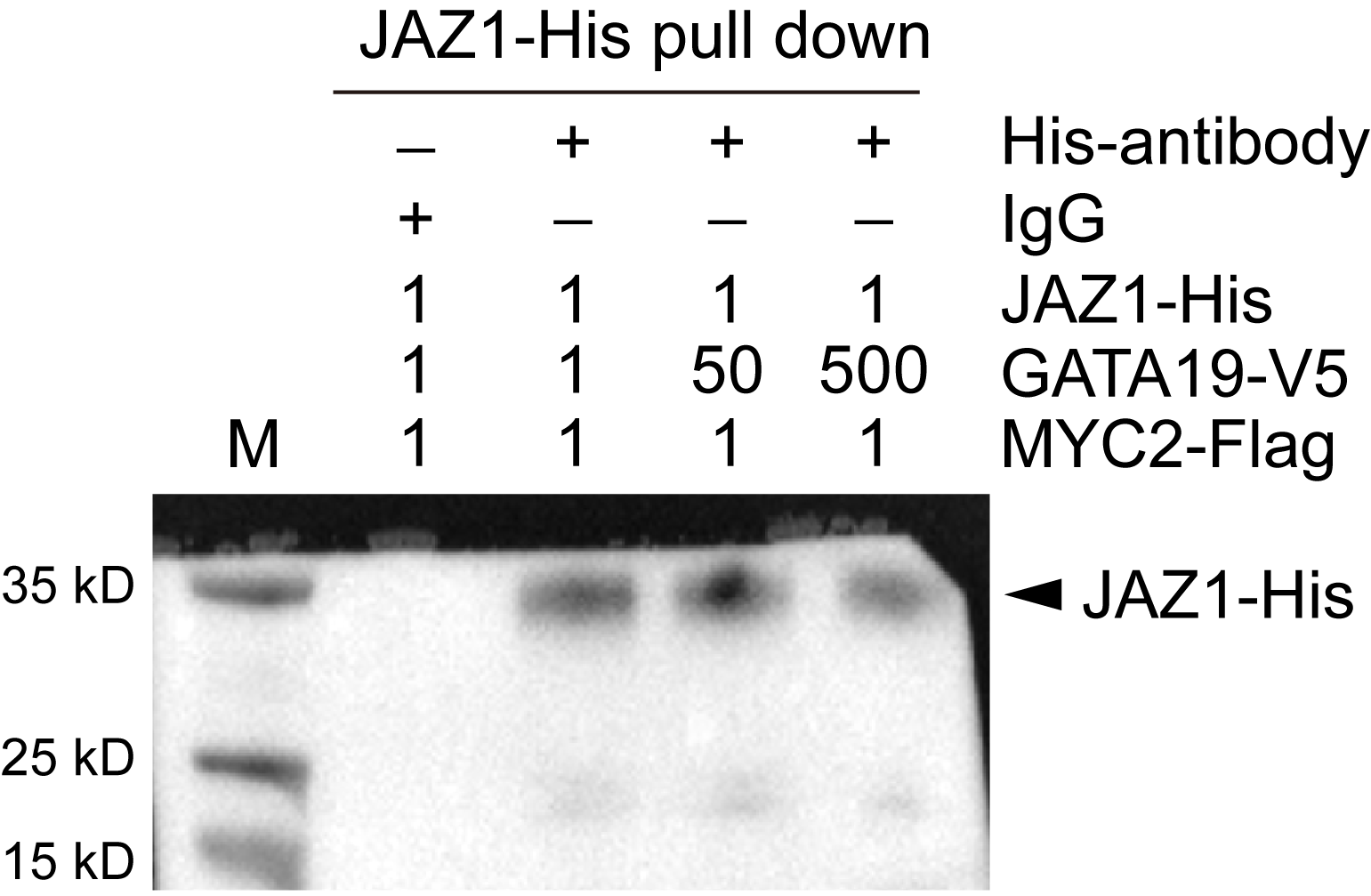

Supplement: Supplementary file 10 — Source data Fig. 6 [file 44318_2025_405_MOESM10_ESM.zip › Figure 6/6G/Pull down-JAZ1His (top right).tif]

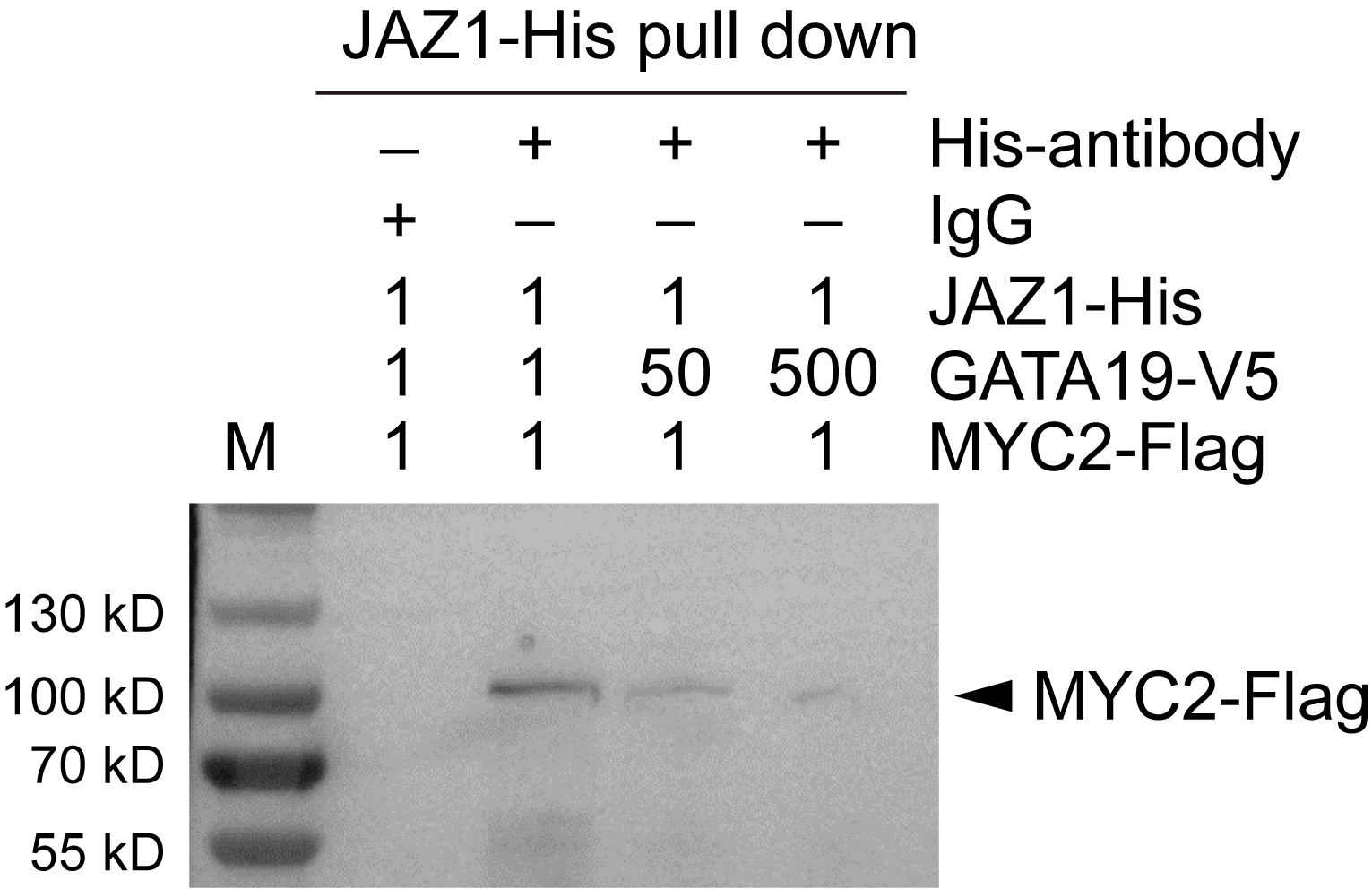

Supplement: Supplementary file 10 — Source data Fig. 6 [file 44318_2025_405_MOESM10_ESM.zip › Figure 6/6G/Pull down-MYC2Flag (bottom right).tif]

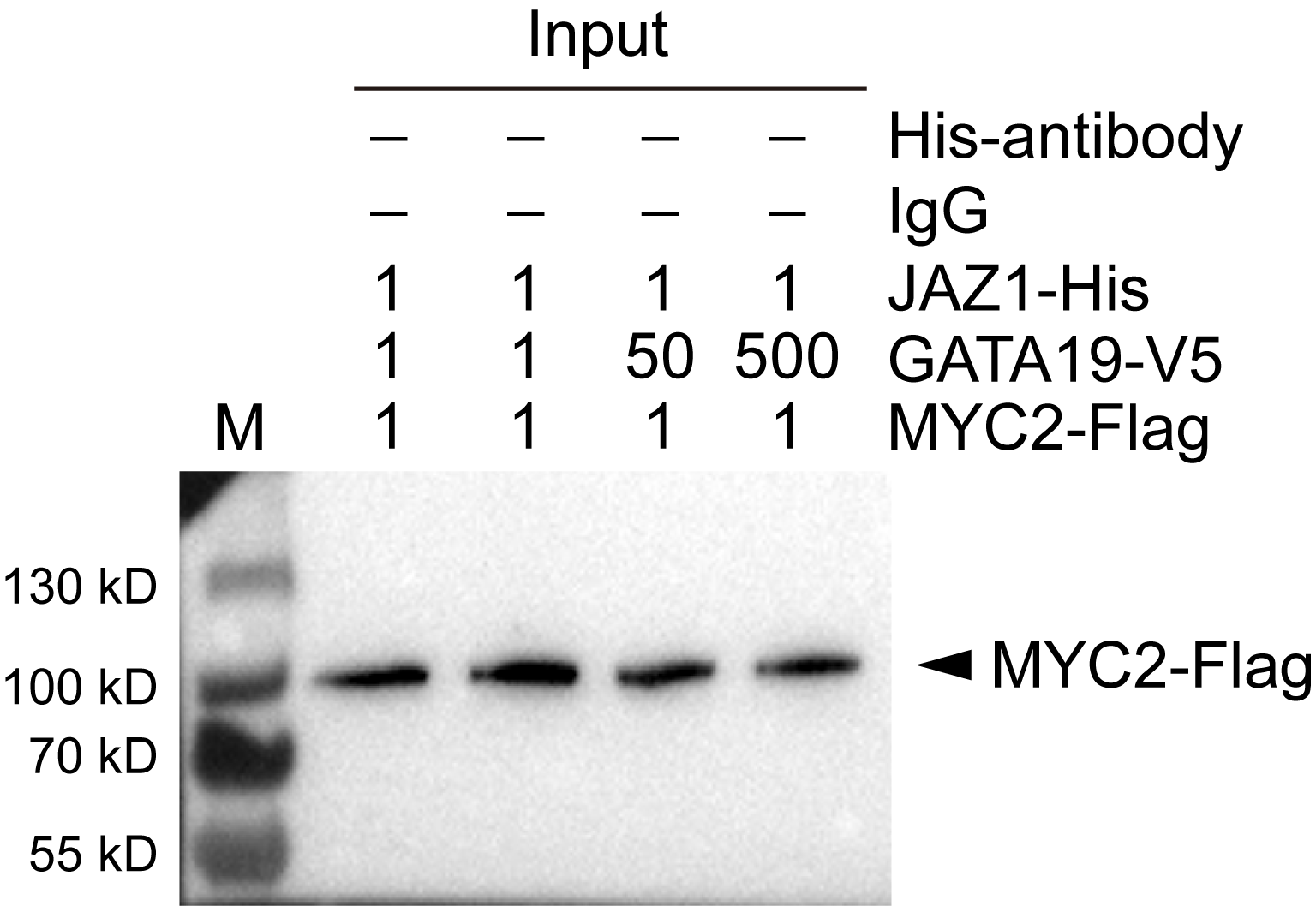

Supplement: Supplementary file 10 — Source data Fig. 6 [file 44318_2025_405_MOESM10_ESM.zip › Figure 6/6G/Input-MYC2Flag (bottom left).tif]

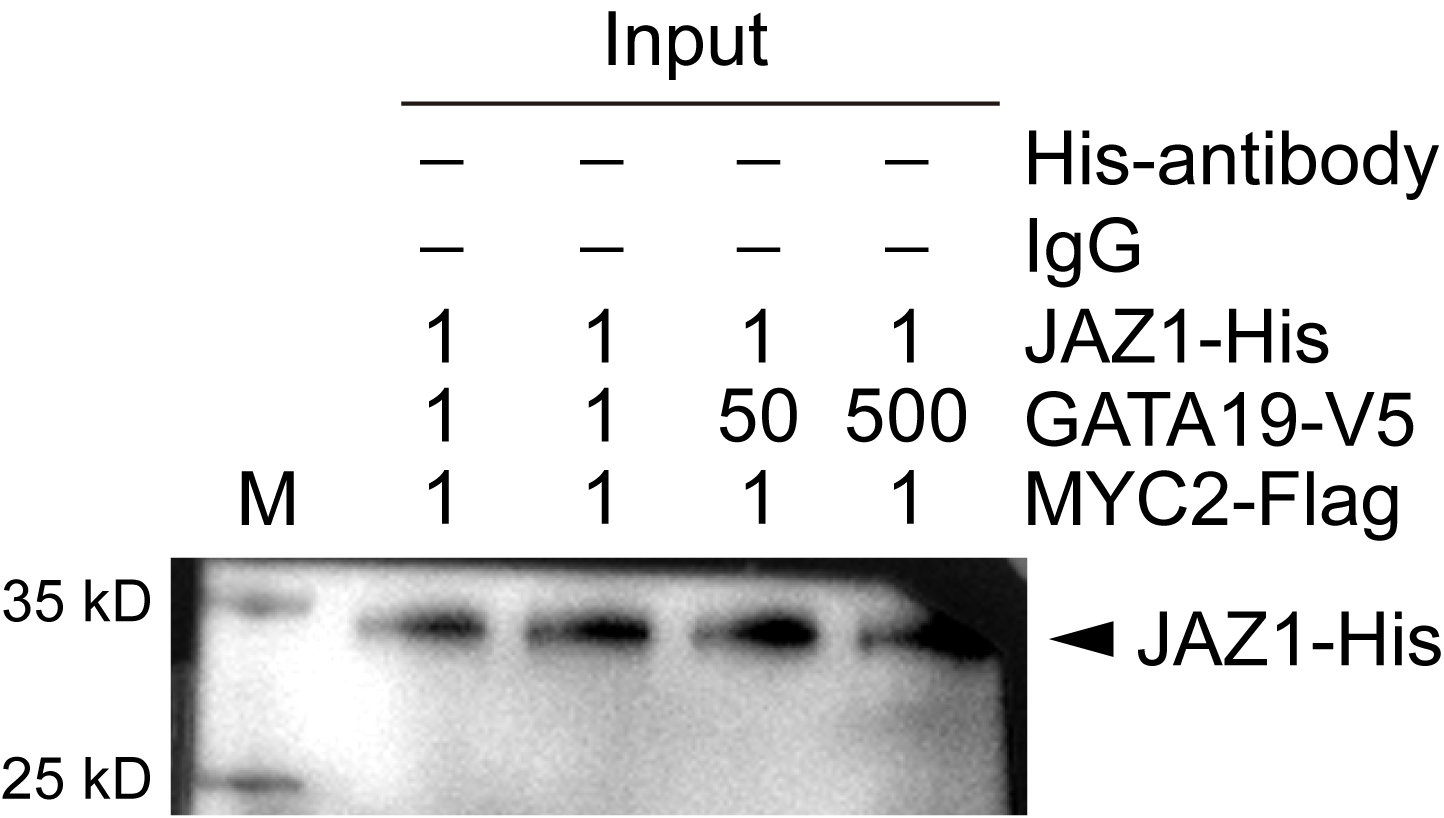

Supplement: Supplementary file 10 — Source data Fig. 6 [file 44318_2025_405_MOESM10_ESM.zip › Figure 6/6G/Input-JAZ1His (top left).tif]

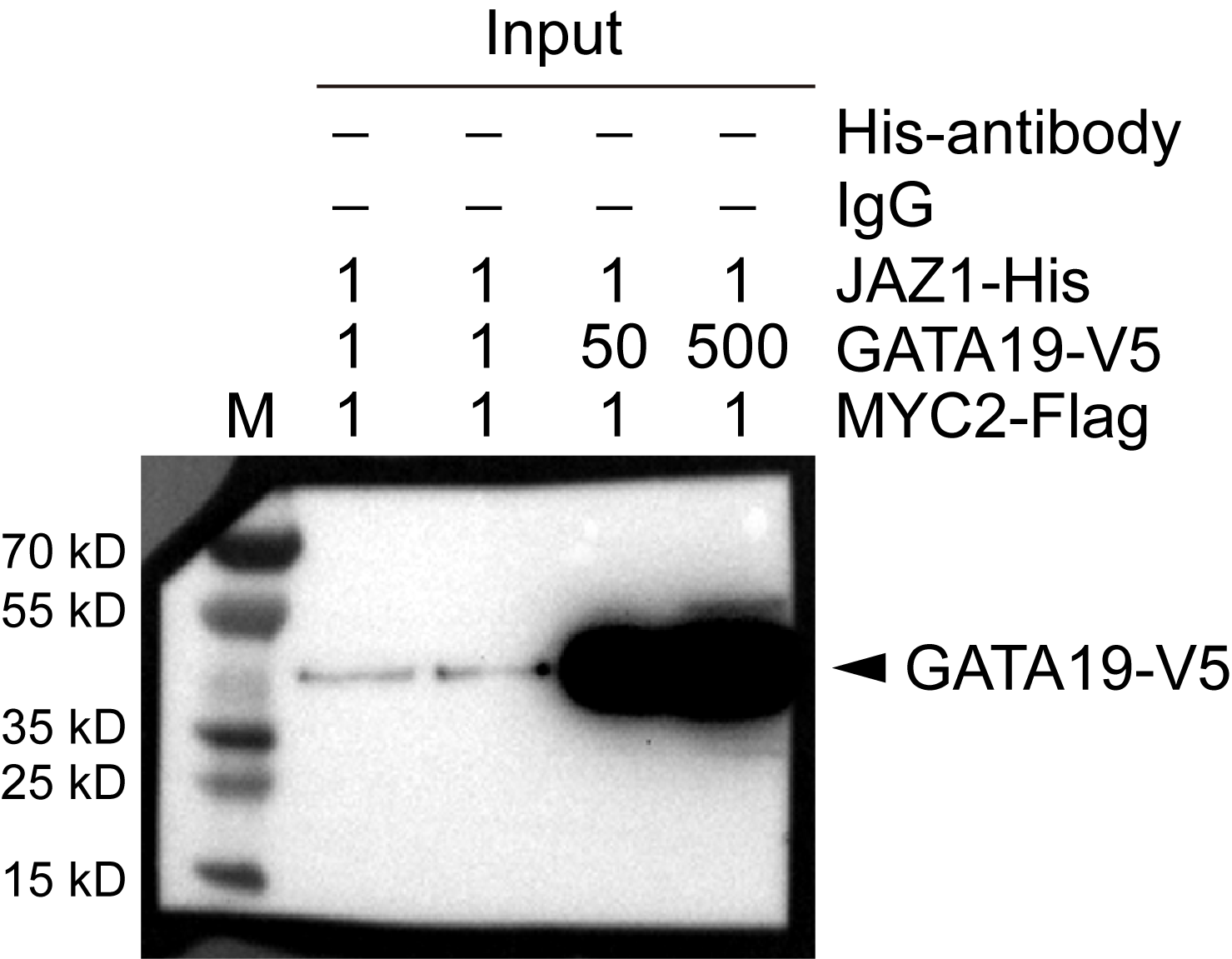

Supplement: Supplementary file 10 — Source data Fig. 6 [file 44318_2025_405_MOESM10_ESM.zip › Figure 6/6G/Western bolt-input-GATA19V5 (middle left).tif]

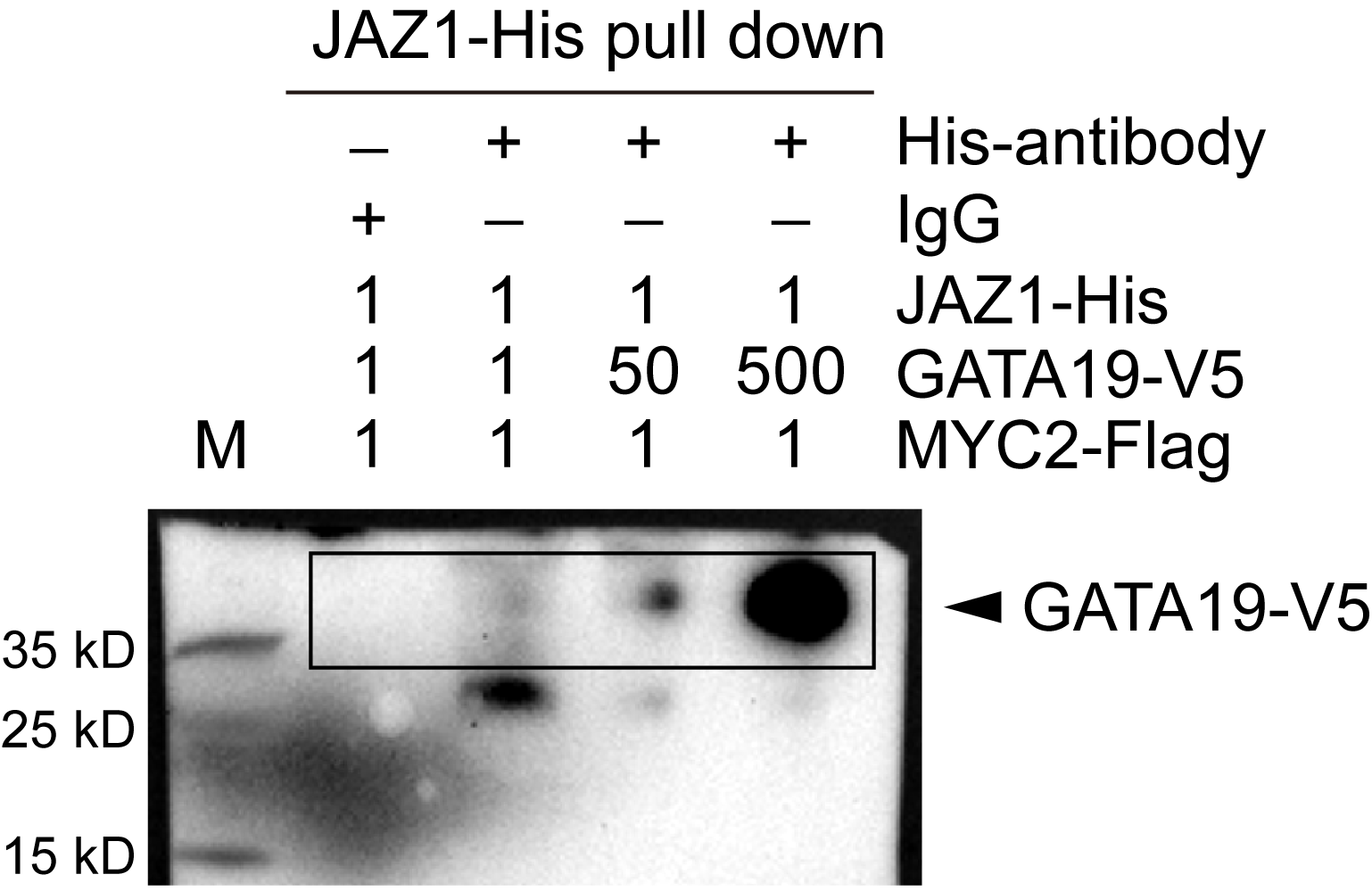

Supplement: Supplementary file 10 — Source data Fig. 6 [file 44318_2025_405_MOESM10_ESM.zip › Figure 6/6G/Pull down-GATA19V5 (middle right).tif]

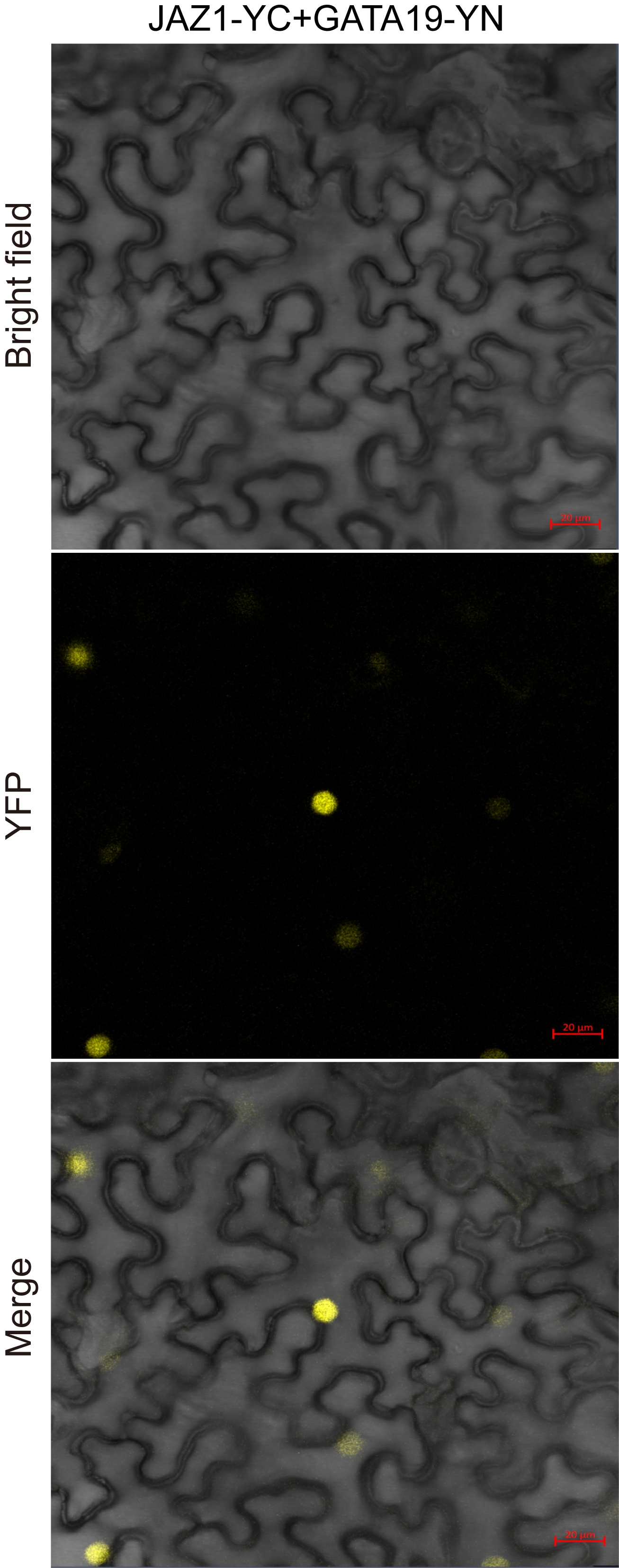

Supplement: Supplementary file 10 — Source data Fig. 6 [file 44318_2025_405_MOESM10_ESM.zip › Figure 6/6E/BiFc JAZ1-YC GATA19-YN (bottom).tif]

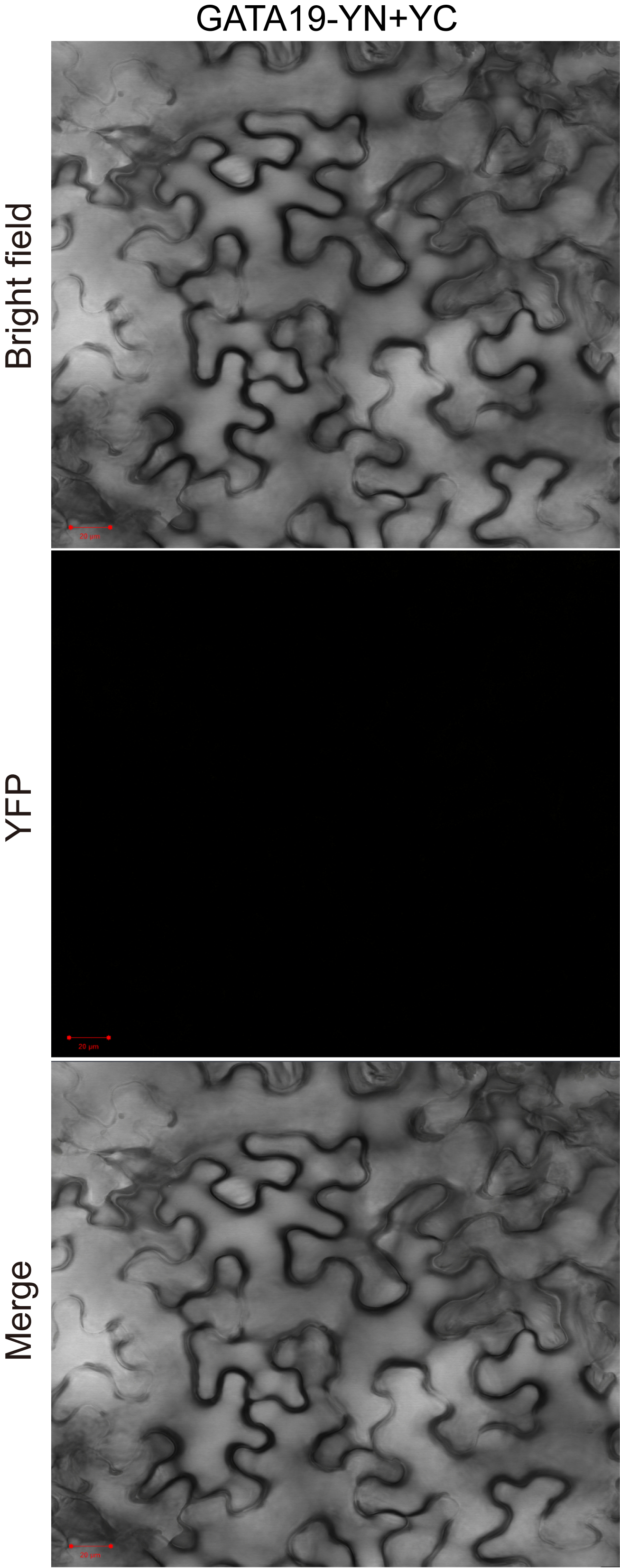

Supplement: Supplementary file 10 — Source data Fig. 6 [file 44318_2025_405_MOESM10_ESM.zip › Figure 6/6E/BiFc GATA19-YN YC (middle).tif]

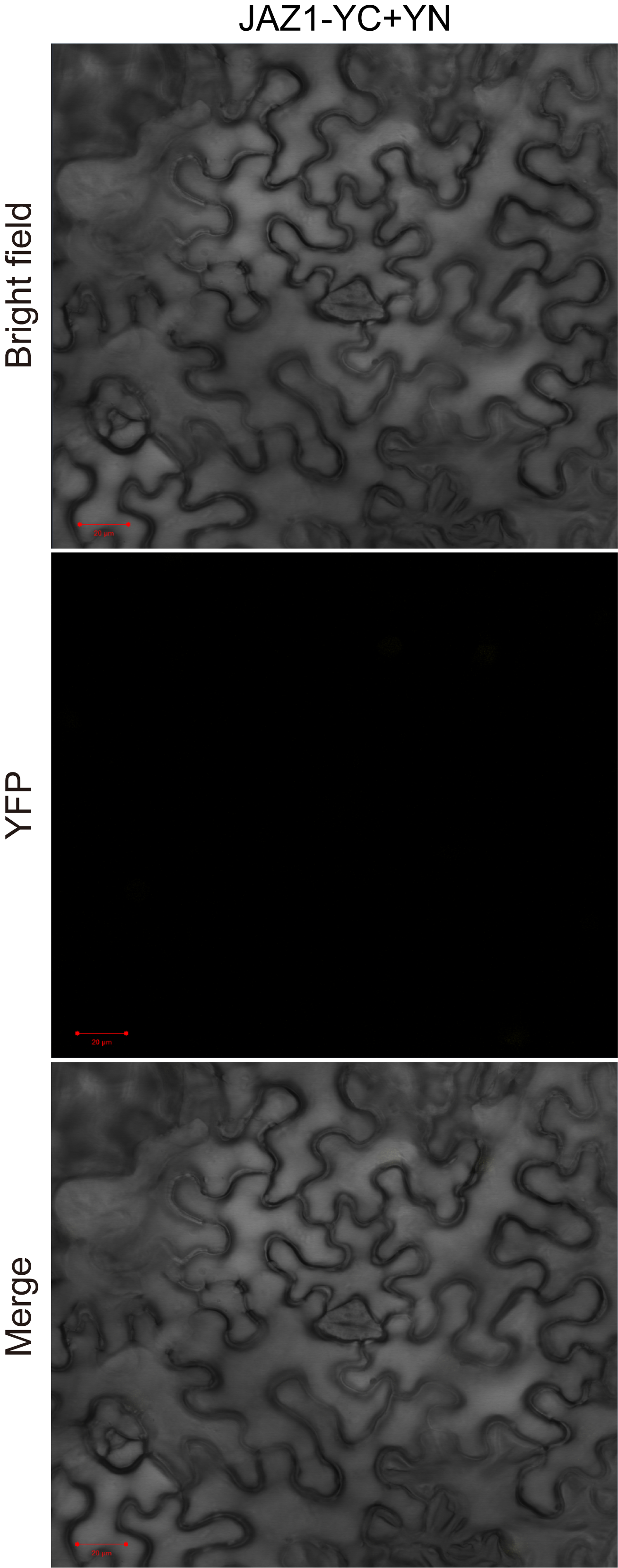

Supplement: Supplementary file 10 — Source data Fig. 6 [file 44318_2025_405_MOESM10_ESM.zip › Figure 6/6E/BiFc JAZ1-YC YN (top).tif]

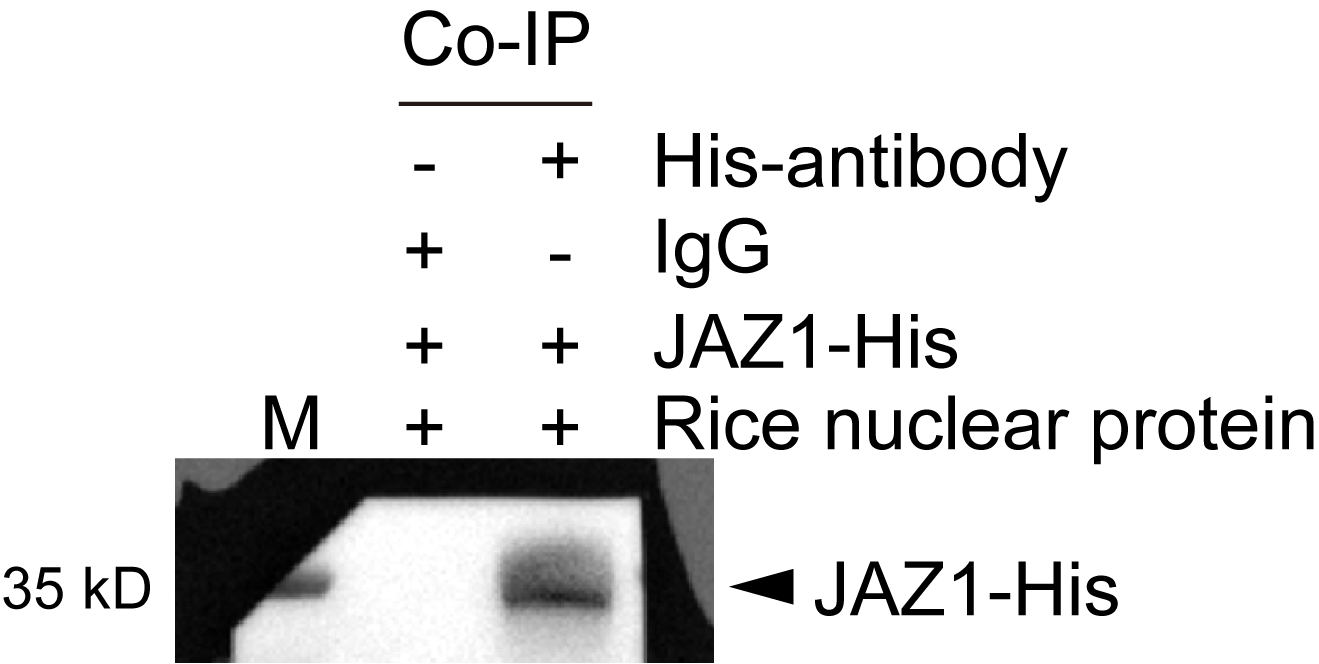

Supplement: Supplementary file 10 — Source data Fig. 6 [file 44318_2025_405_MOESM10_ESM.zip › Figure 6/6D/CoIP-JAZ1His (top right).tif]

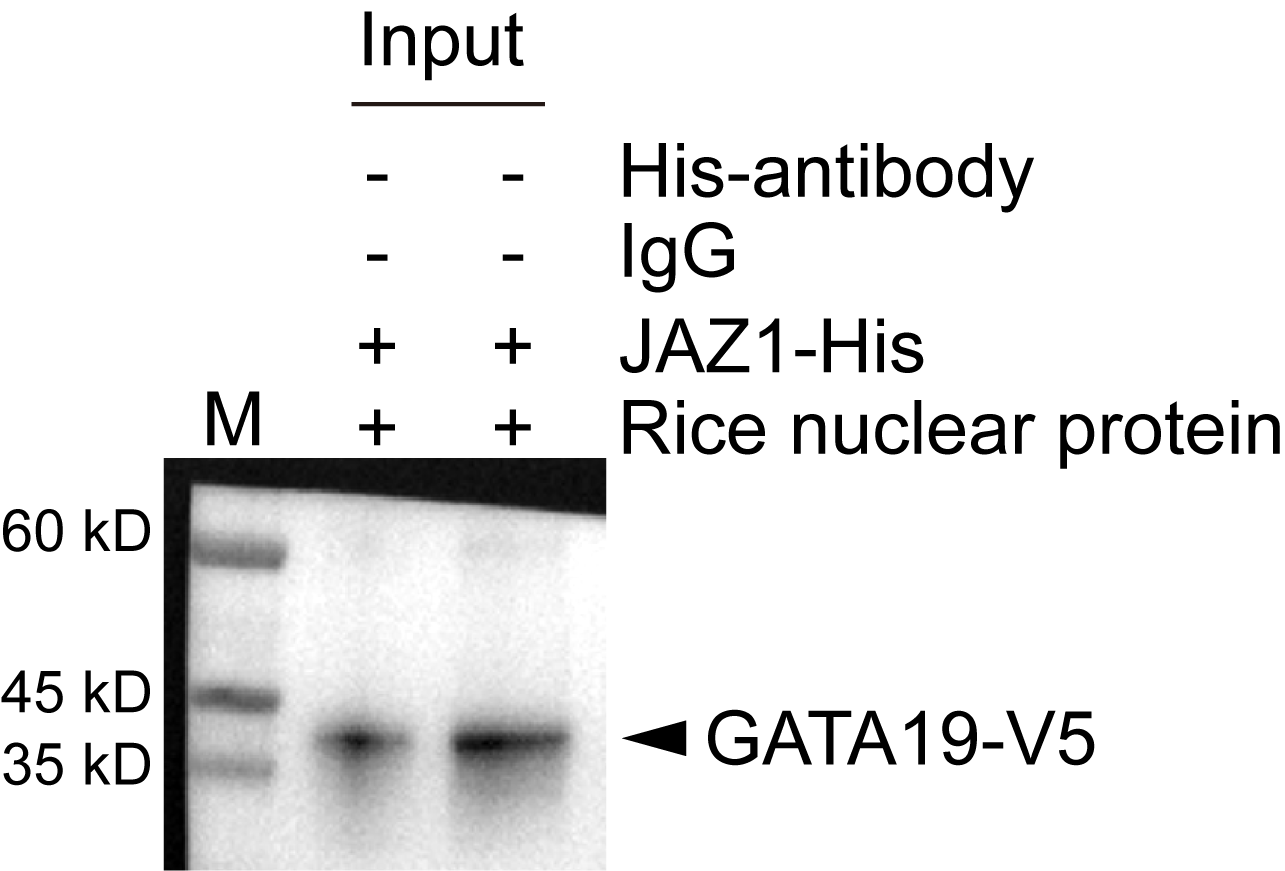

Supplement: Supplementary file 10 — Source data Fig. 6 [file 44318_2025_405_MOESM10_ESM.zip › Figure 6/6D/Input-GATA19V5 (bottom left).tif]

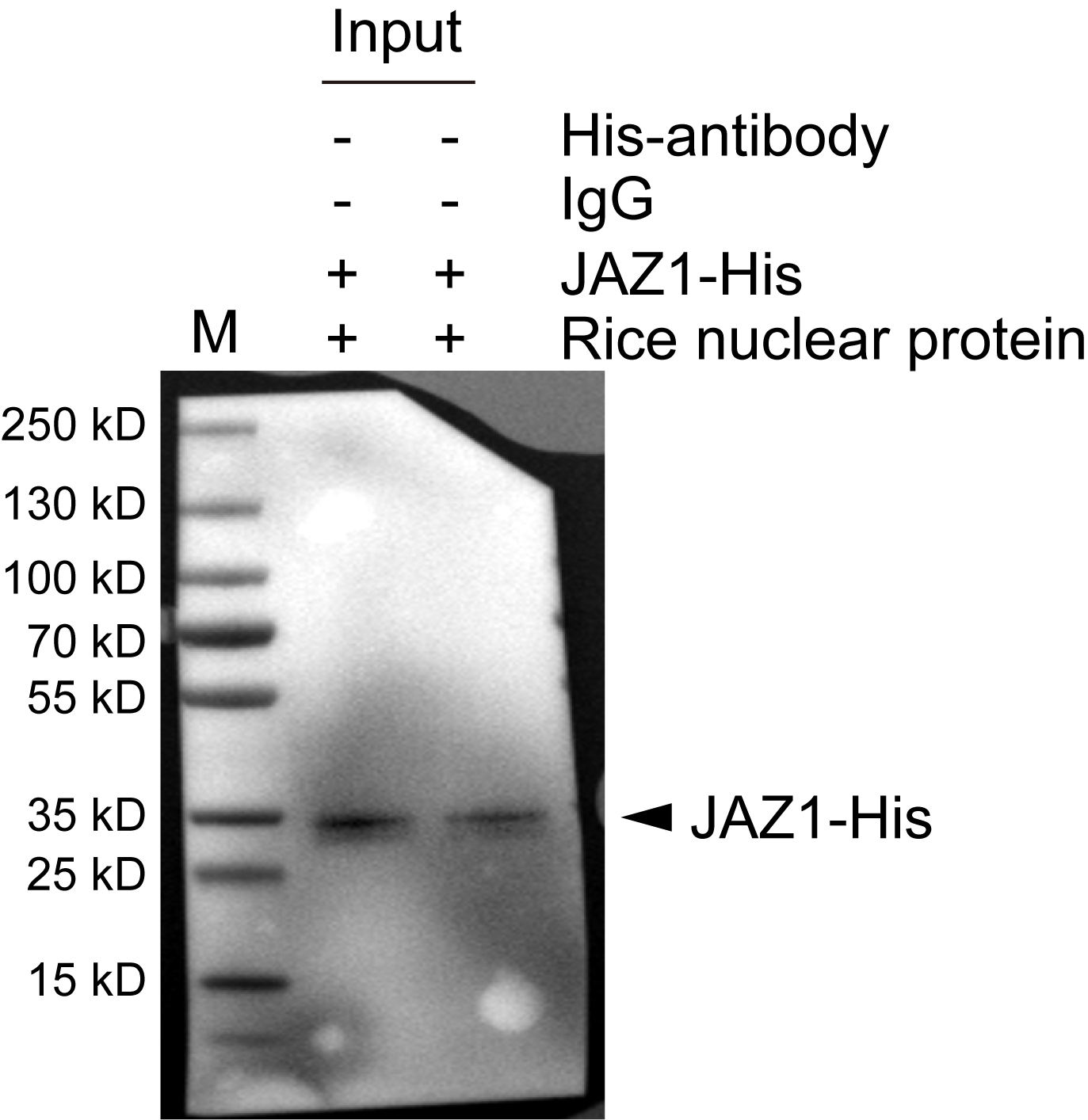

Supplement: Supplementary file 10 — Source data Fig. 6 [file 44318_2025_405_MOESM10_ESM.zip › Figure 6/6D/Input-JAZ1His (top left).tif]

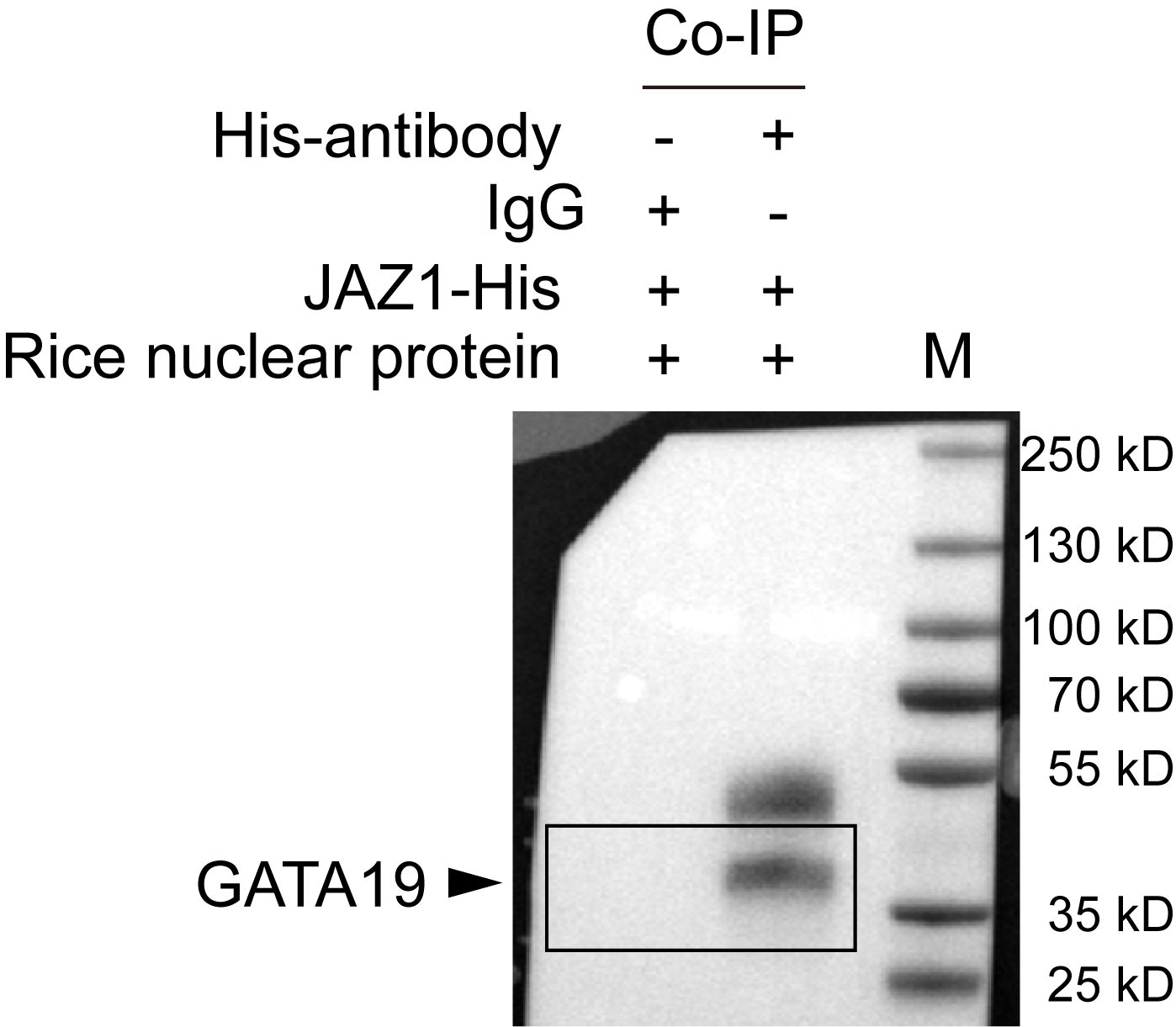

Supplement: Supplementary file 10 — Source data Fig. 6 [file 44318_2025_405_MOESM10_ESM.zip › Figure 6/6D/CoIP-GATA19 (bottom right).tif]

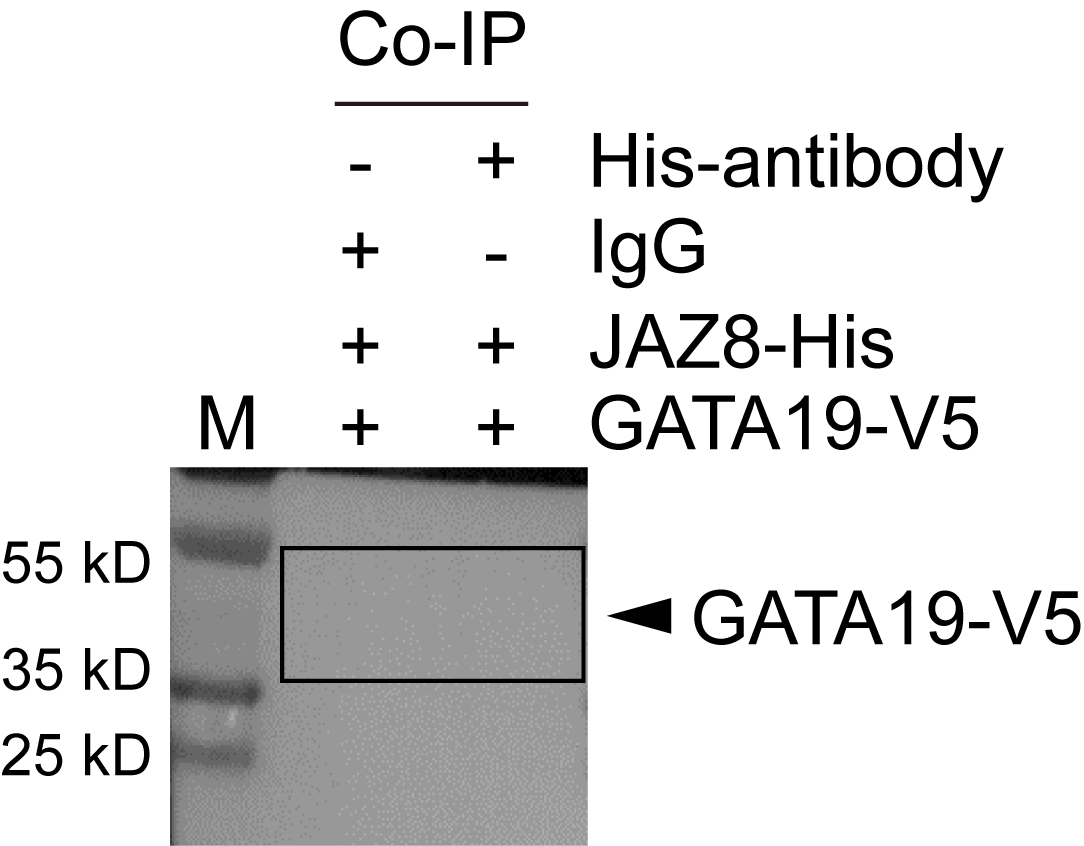

Supplement: Supplementary file 10 — Source data Fig. 6 [file 44318_2025_405_MOESM10_ESM.zip › Figure 6/6C/JAZ8His-GATA19V5 (middle)/CoIP-GATA19V5 (bottom right).tif]

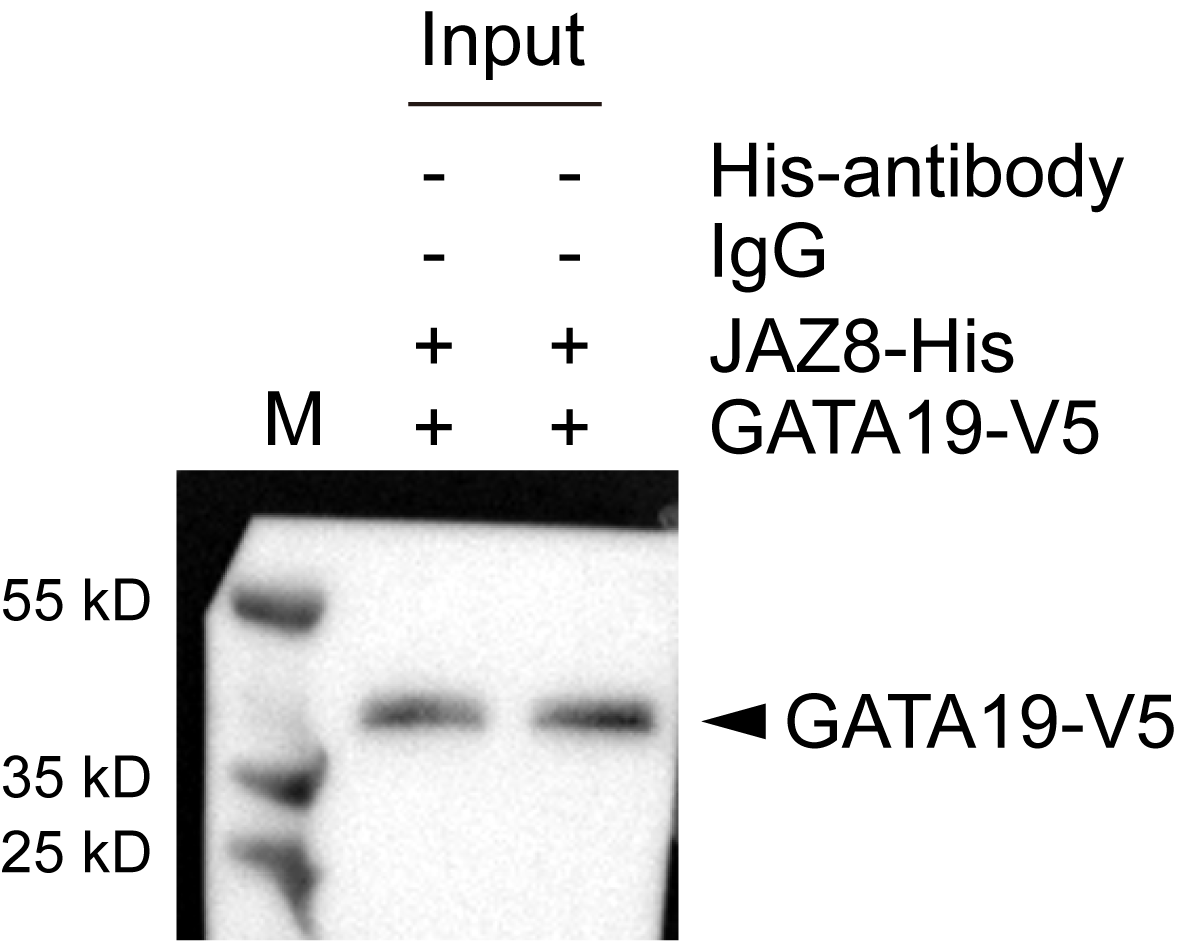

Supplement: Supplementary file 10 — Source data Fig. 6 [file 44318_2025_405_MOESM10_ESM.zip › Figure 6/6C/JAZ8His-GATA19V5 (middle)/Input-GATA19V5 (bottom left).tif]

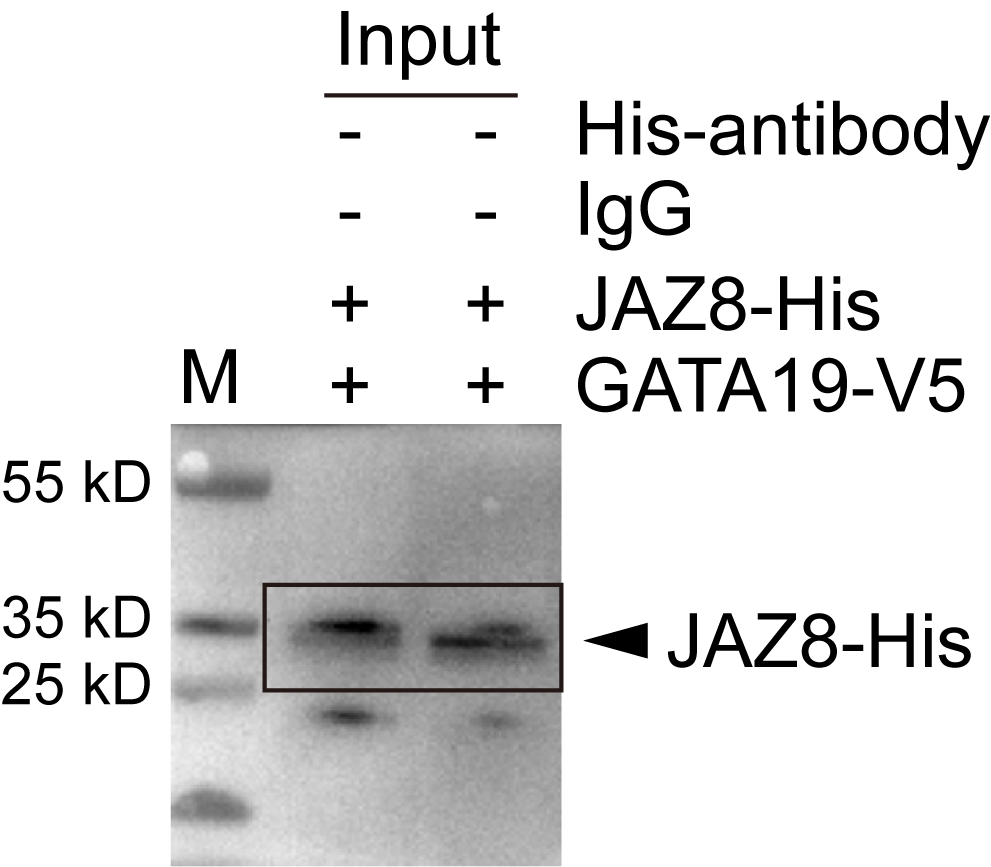

Supplement: Supplementary file 10 — Source data Fig. 6 [file 44318_2025_405_MOESM10_ESM.zip › Figure 6/6C/JAZ8His-GATA19V5 (middle)/Input-JAZ8His (top left).tif]

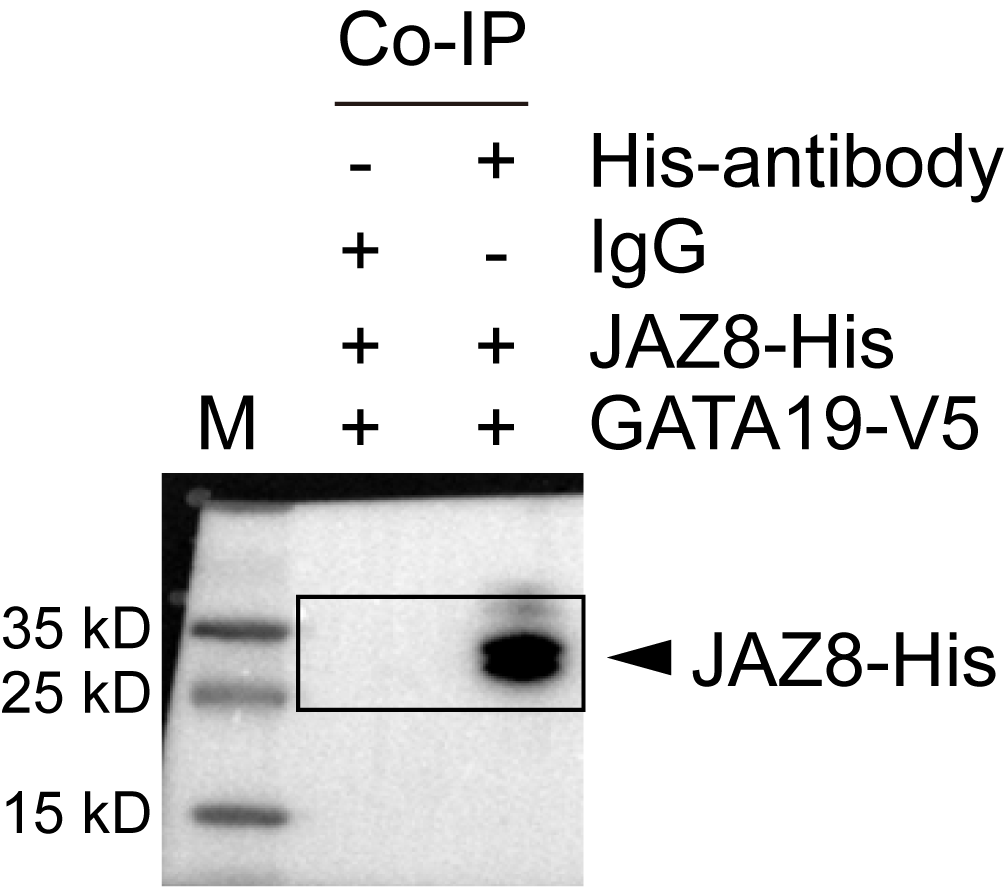

Supplement: Supplementary file 10 — Source data Fig. 6 [file 44318_2025_405_MOESM10_ESM.zip › Figure 6/6C/JAZ8His-GATA19V5 (middle)/CoIP-JAZ8His (top right).tif]

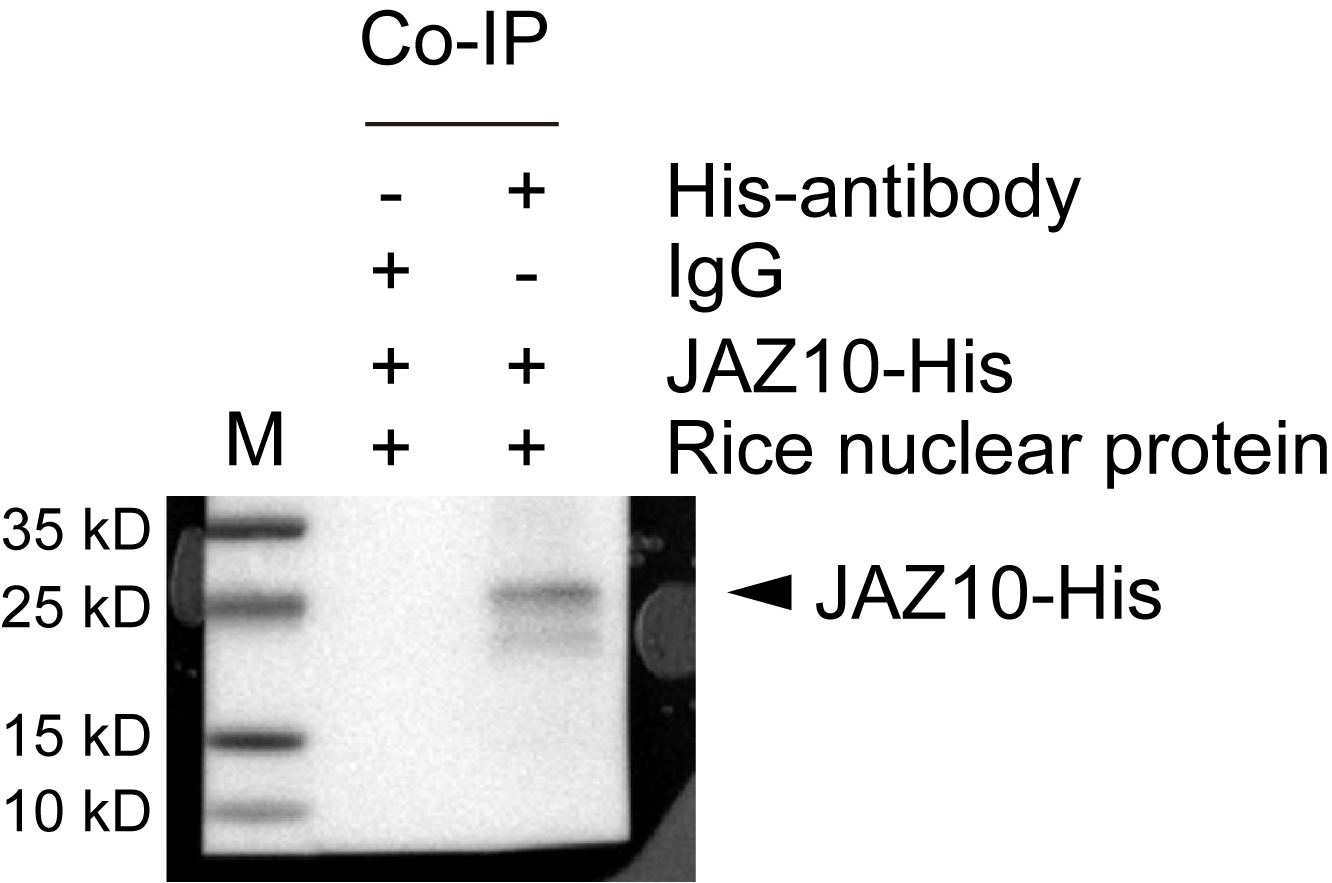

Supplement: Supplementary file 10 — Source data Fig. 6 [file 44318_2025_405_MOESM10_ESM.zip › Figure 6/6C/JAZ10His-GATA19V5 (bottom)/CoIP-JAZ10His (top right).tif]

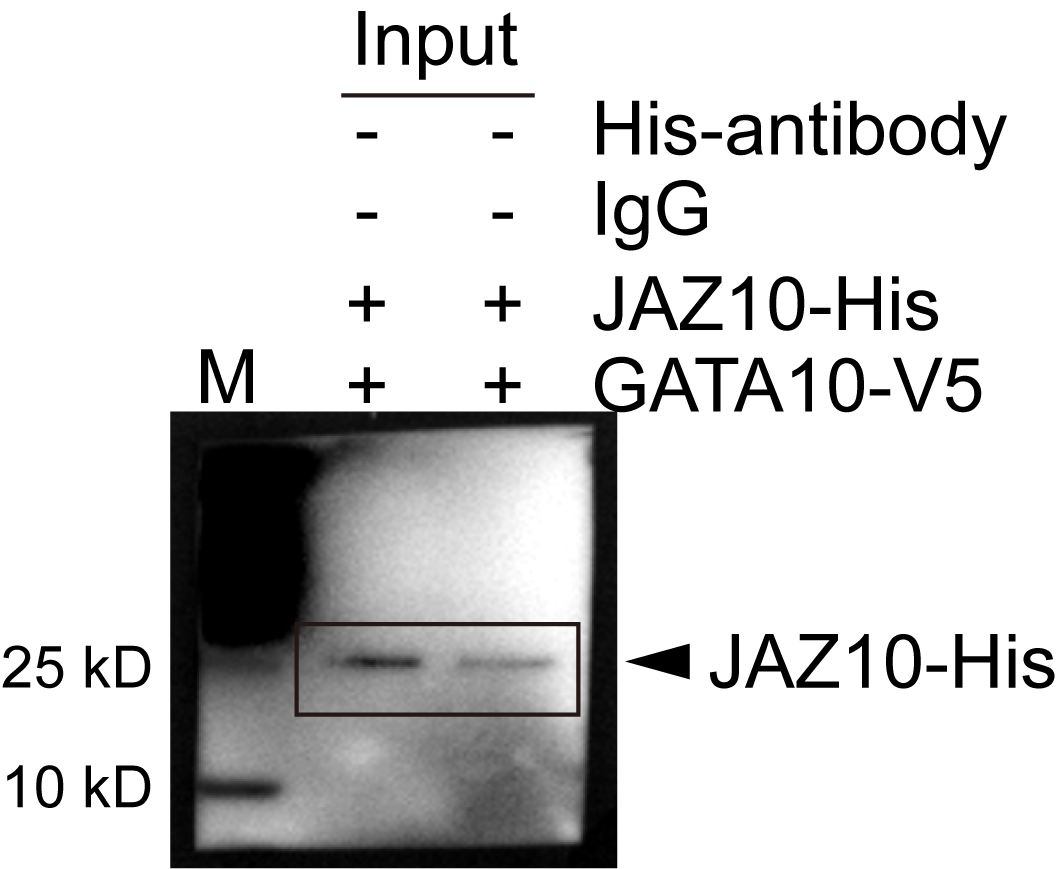

Supplement: Supplementary file 10 — Source data Fig. 6 [file 44318_2025_405_MOESM10_ESM.zip › Figure 6/6C/JAZ10His-GATA19V5 (bottom)/6C-JAZ10-Input-JAZ10His (top left).tif]

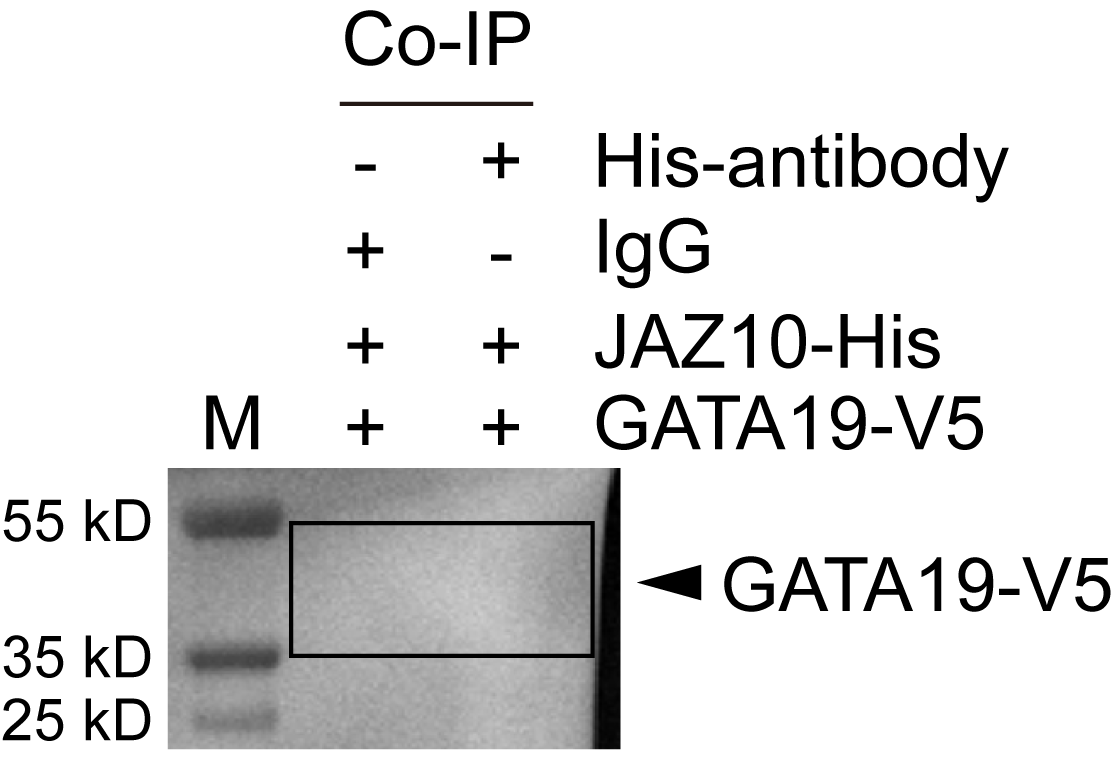

Supplement: Supplementary file 10 — Source data Fig. 6 [file 44318_2025_405_MOESM10_ESM.zip › Figure 6/6C/JAZ10His-GATA19V5 (bottom)/CoIP-GATA19V5 (bottom right).tif]

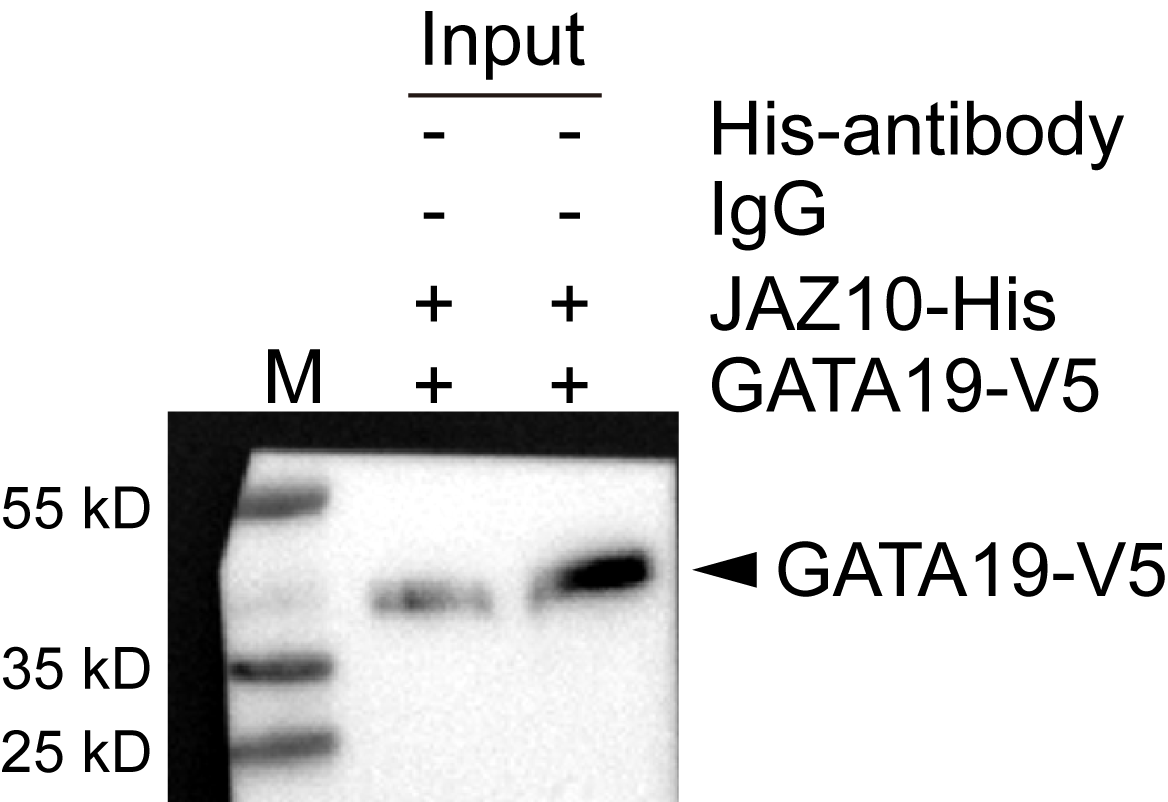

Supplement: Supplementary file 10 — Source data Fig. 6 [file 44318_2025_405_MOESM10_ESM.zip › Figure 6/6C/JAZ10His-GATA19V5 (bottom)/Input-GATA19V5 (bottom left).tif]

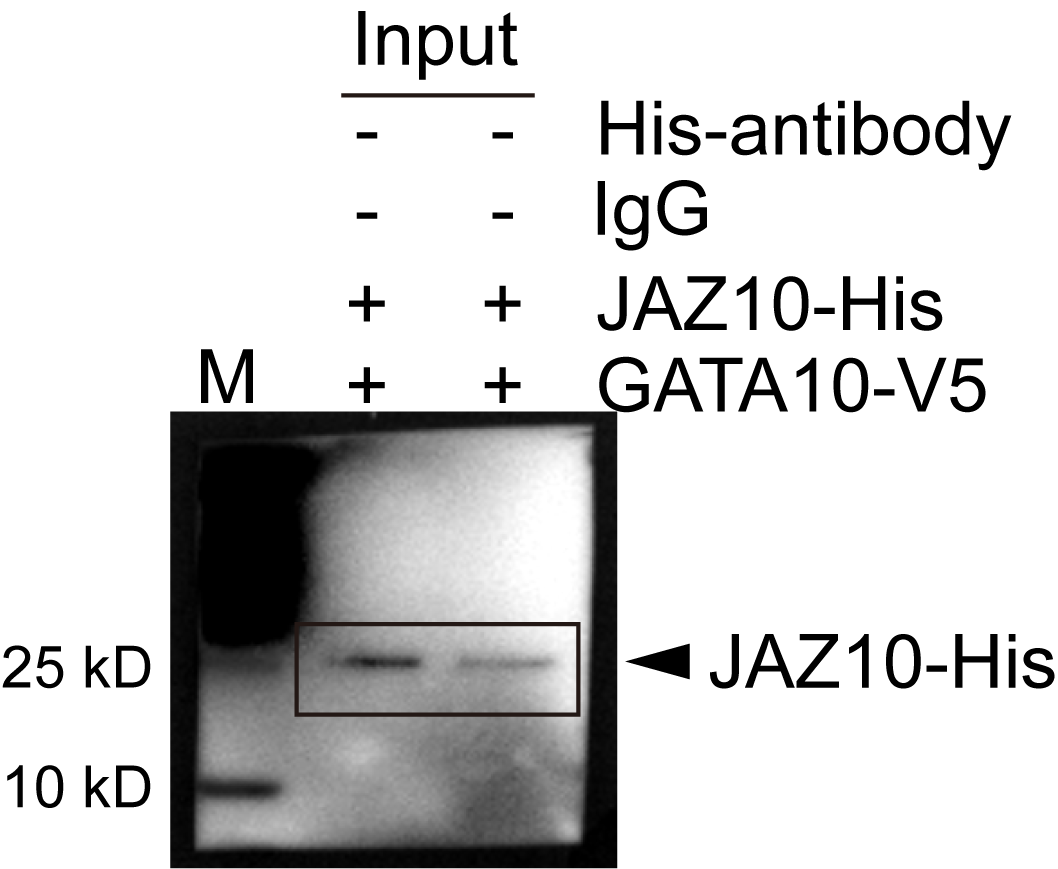

Supplement: Supplementary file 10 — Source data Fig. 6 [file 44318_2025_405_MOESM10_ESM.zip › Figure 6/6C/JAZ10His-GATA19V5 (bottom)/Input-JAZ10His (top left).tif]

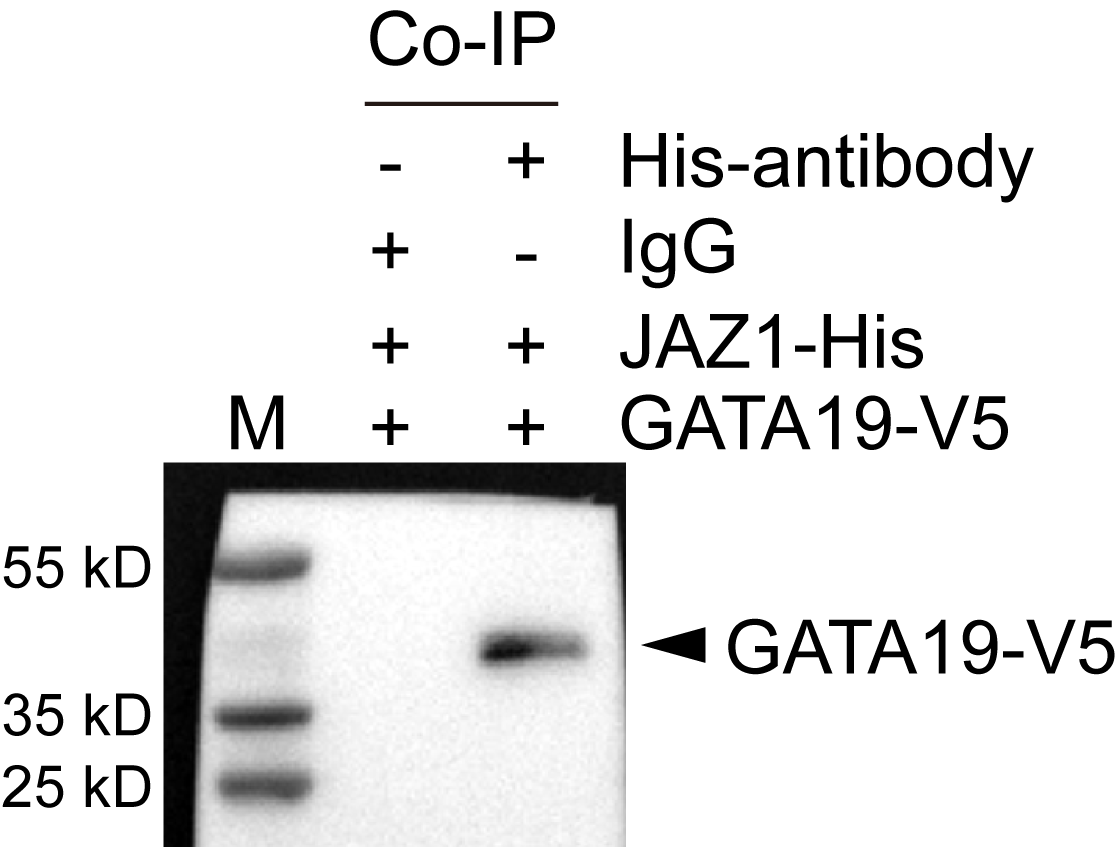

Supplement: Supplementary file 10 — Source data Fig. 6 [file 44318_2025_405_MOESM10_ESM.zip › Figure 6/6C/JAZ1His-GATA19V5 (top)/CoIP-GATA19V5 (bottom right).tif]

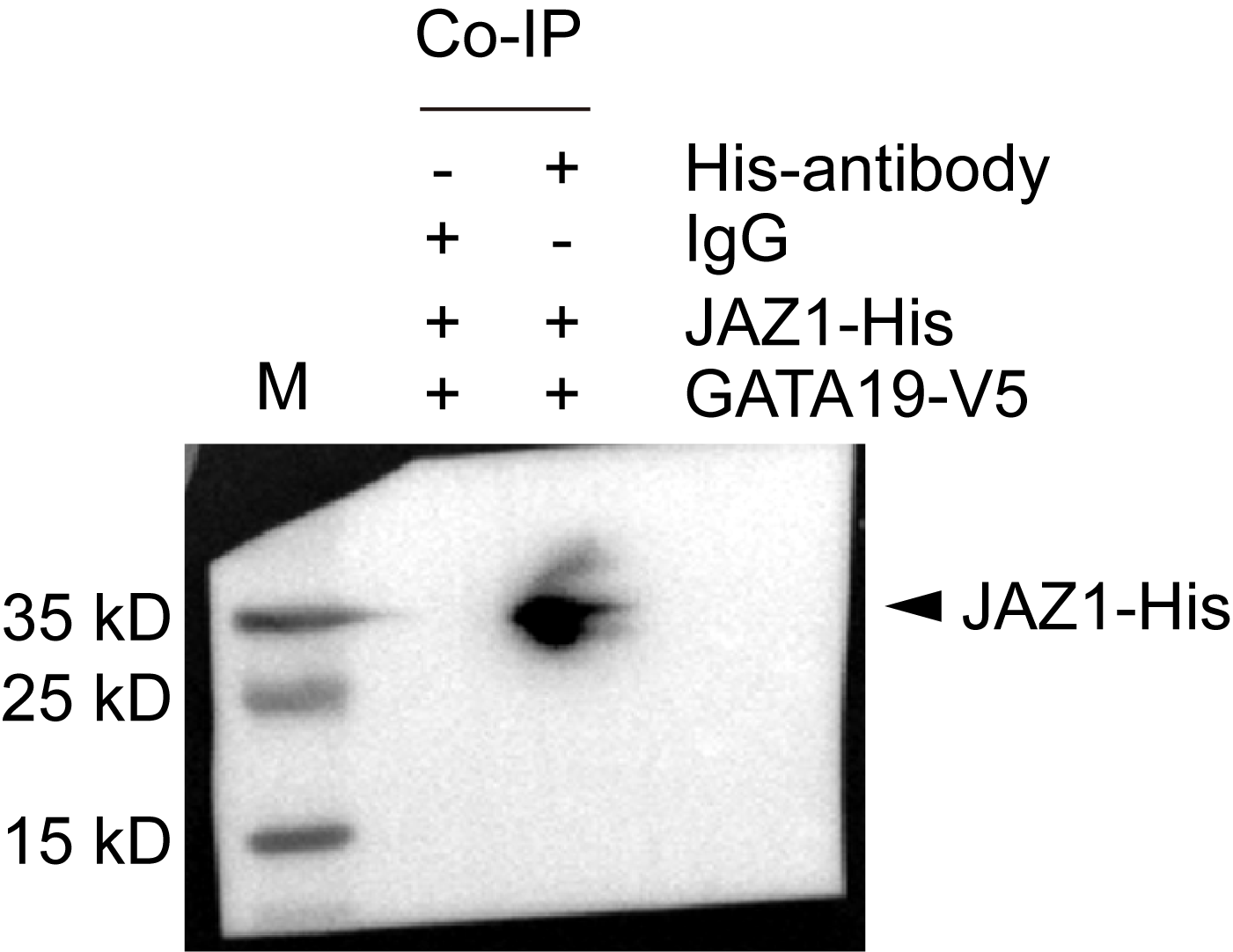

Supplement: Supplementary file 10 — Source data Fig. 6 [file 44318_2025_405_MOESM10_ESM.zip › Figure 6/6C/JAZ1His-GATA19V5 (top)/CoIP-JAZ1His (top right).tif]

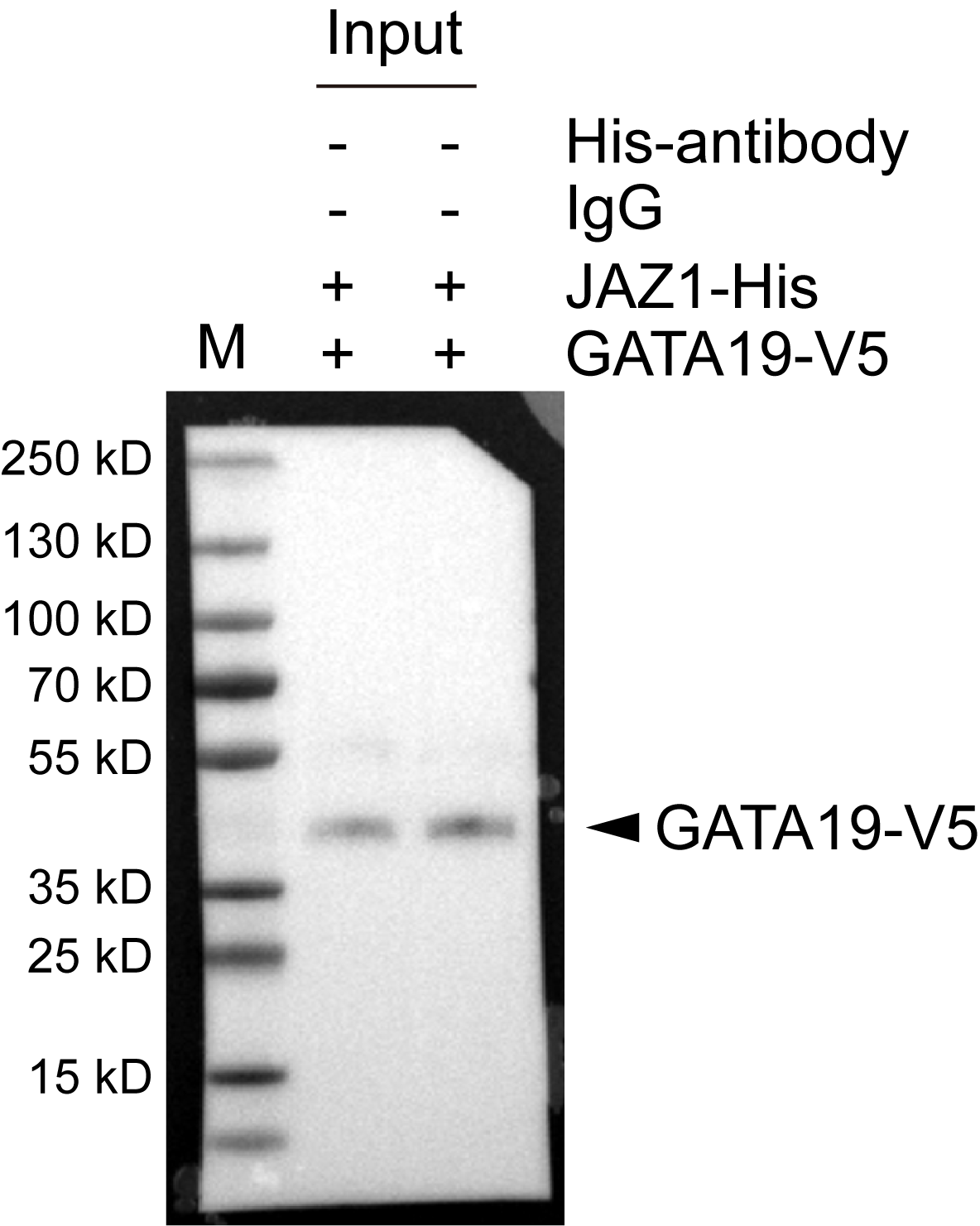

Supplement: Supplementary file 10 — Source data Fig. 6 [file 44318_2025_405_MOESM10_ESM.zip › Figure 6/6C/JAZ1His-GATA19V5 (top)/Input-GATA19V5 (bottom left).tif]

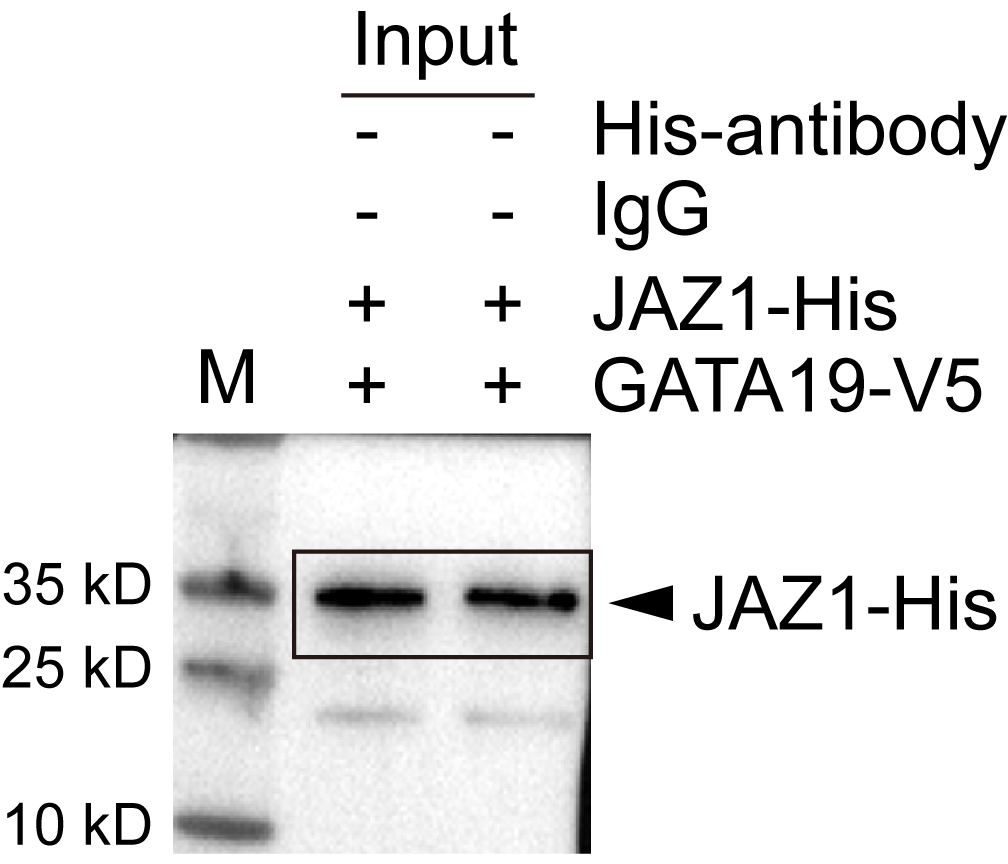

Supplement: Supplementary file 10 — Source data Fig. 6 [file 44318_2025_405_MOESM10_ESM.zip › Figure 6/6C/JAZ1His-GATA19V5 (top)/Input-JAZ1His (top left).tif]
