# Supplementary material for: A double-agent microRNA regulates viral cross-kingdom infection in animals and plants
Source: EMBO J. 2025 Mar 5;44(9):2446–72. doi: 10.1038/s44318-025-00405-4 (PMC12048567; doi:10.1038/s44318-025-00405-4)
Supplement: Supplementary file 11 — Expanded View Figures [file 44318_2025_405_MOESM11_ESM.pdf]

Expanded View Figures

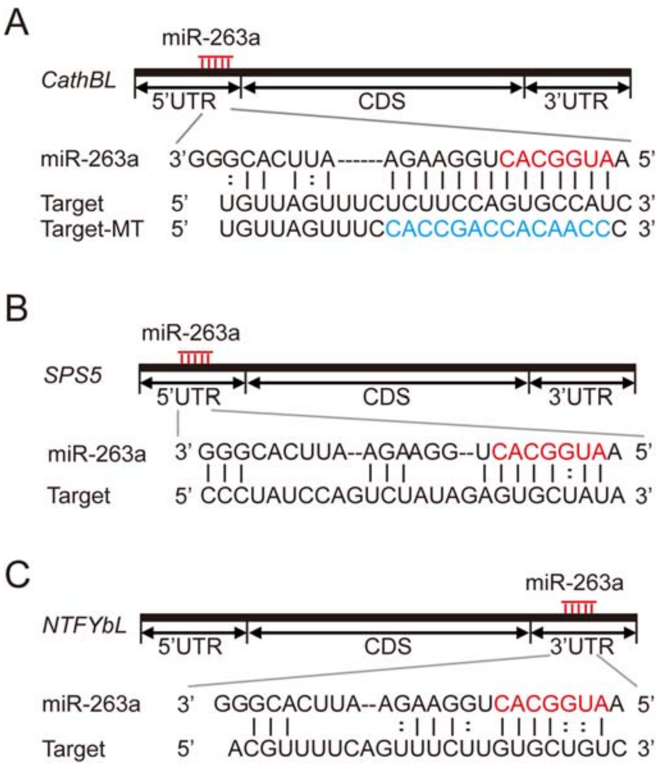

Figure EV1. Sequence alignments of miR-263a with the candidate target genes of SBPH.

(**A**) *Cathepsin B-like* (*CathBL*). The mutated target (Target-MT) is also shown. (**B**) *Nuclear transcription factor Y subunit beta-like* (*NTFYbL*). (**C**) *Serine protease snake-5* (*SPS5*). The miR-263a seed sequence is highlighted in red and the mutated sequences are indicated in blue.

© The Author(s)

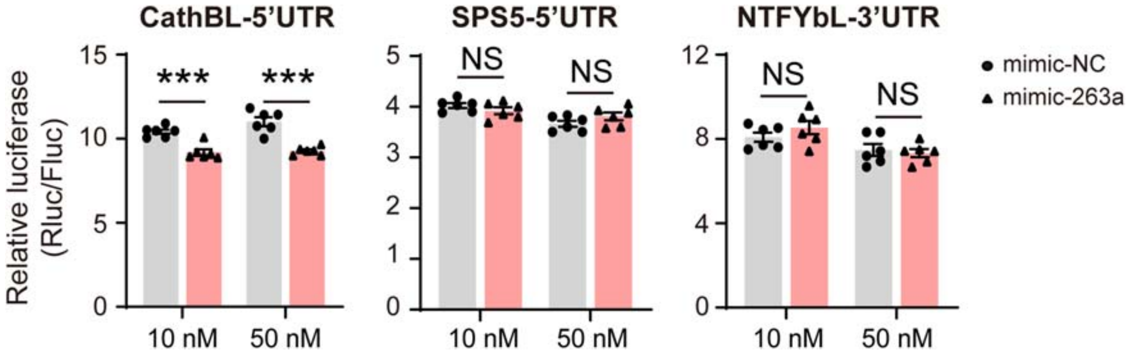

Figure EV2. Luciferase reporter assays in *Drosophila* S2 cells cotransfected with miR-263a/NC mimics and psiCHECK2 vectors containing *CathBL* 5′UTR, *SPS5* 5′ UTR, and *NTFYbL* 3′UTR as the candidate targets of miR-263a.

P values from left to right, $P = 2.83E{-}04$, $P = 1.27E{-}04$. Six biological replicates were prepared. Values are shown as mean ± SE and were compared using Student's t test. NS, no significant difference. ***$P < 0.001$.

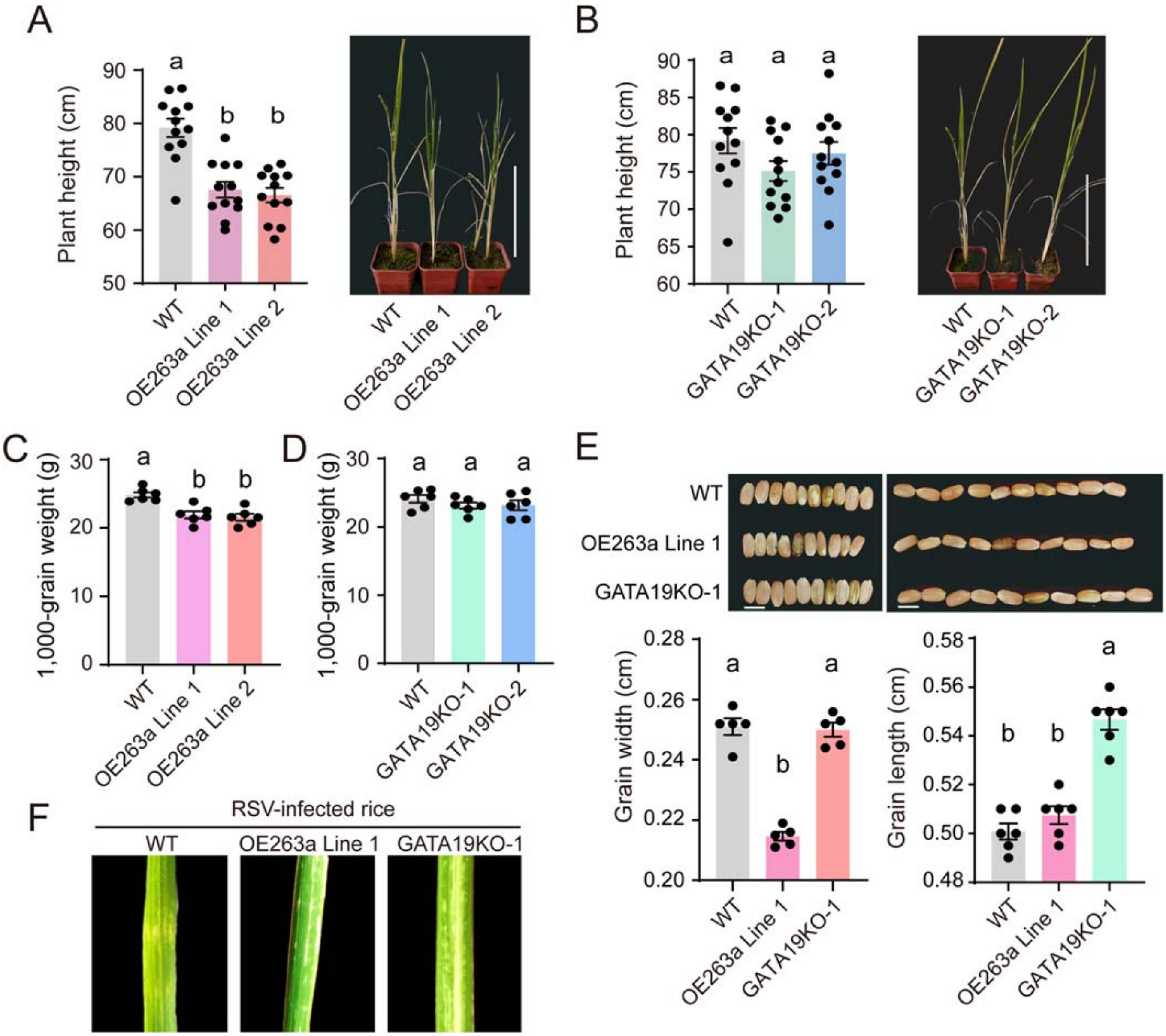

Figure EV3. Gross morphology of rice plants.

(**A**) Comparative plant height between WT and OE263a lines, with a scale bar representing 50 cm. Twelve biological replicates were prepared, with each replicate including one rice plant. (**B**) Plant height comparison between WT and *GATA19*KO lines, utilizing the same scale bar for reference. Twelve biological replicates were prepared, with each replicate including one rice plant. (**C**) The 1000-grain weight of WT and OE263a lines. Six biological replicates were prepared. (**D**) The 1000-grain weight of WT and *GATA19*KO lines. Six biological replicates were prepared. (**E**) Husked grain width and length of WT, OE263a, and *GATA19*KO lines. Scale bars: 5 mm. Five to six biological replicates were prepared, with each replicate including 10 grains. (**F**) Disease symptom of WT, OE263a, and *GATA19*KO lines after inoculation with RSV. For (**A**) to (**E**), the values are reported as mean ± SE. Data comparisons among multiple groups were performed using one-way analysis of variance (ANOVA) followed by Tukey's test. Different letters indicate significant differences.

 © The Author(s)

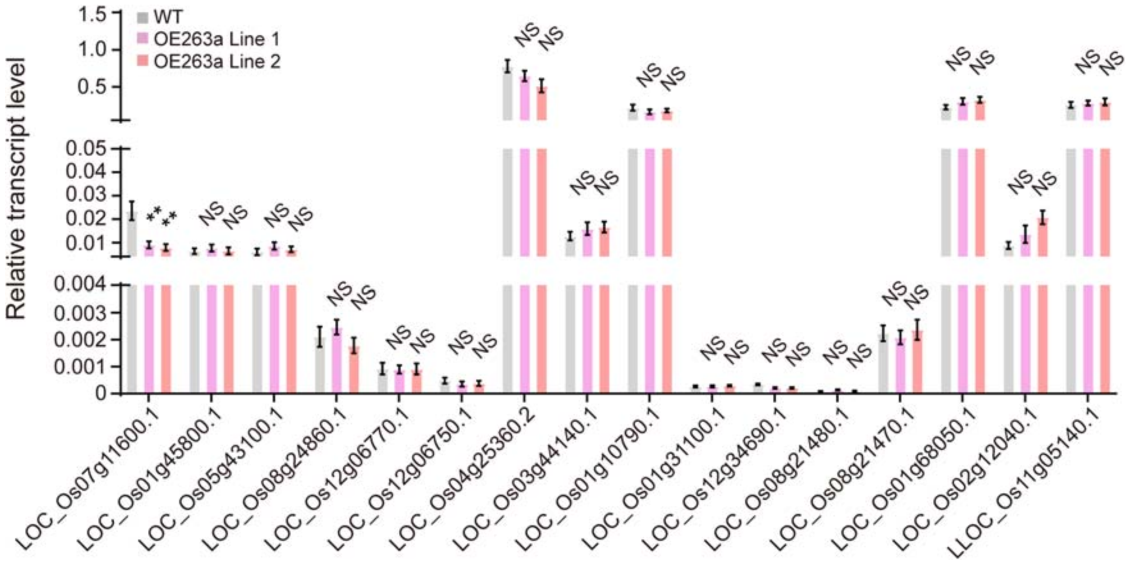

Figure EV4. Relative transcript levels of 16 candidate target genes of miR-263a in WT and OE263a rice lines.

Eight biological replicates were prepared, with each replicate including two leaves. *P* values from left to right, $P = 0.0041$, $P = 0.0062$. Values are reported as mean ± SE and were compared by Student's t test. NS, no significant difference. $^{**}P < 0.01$.

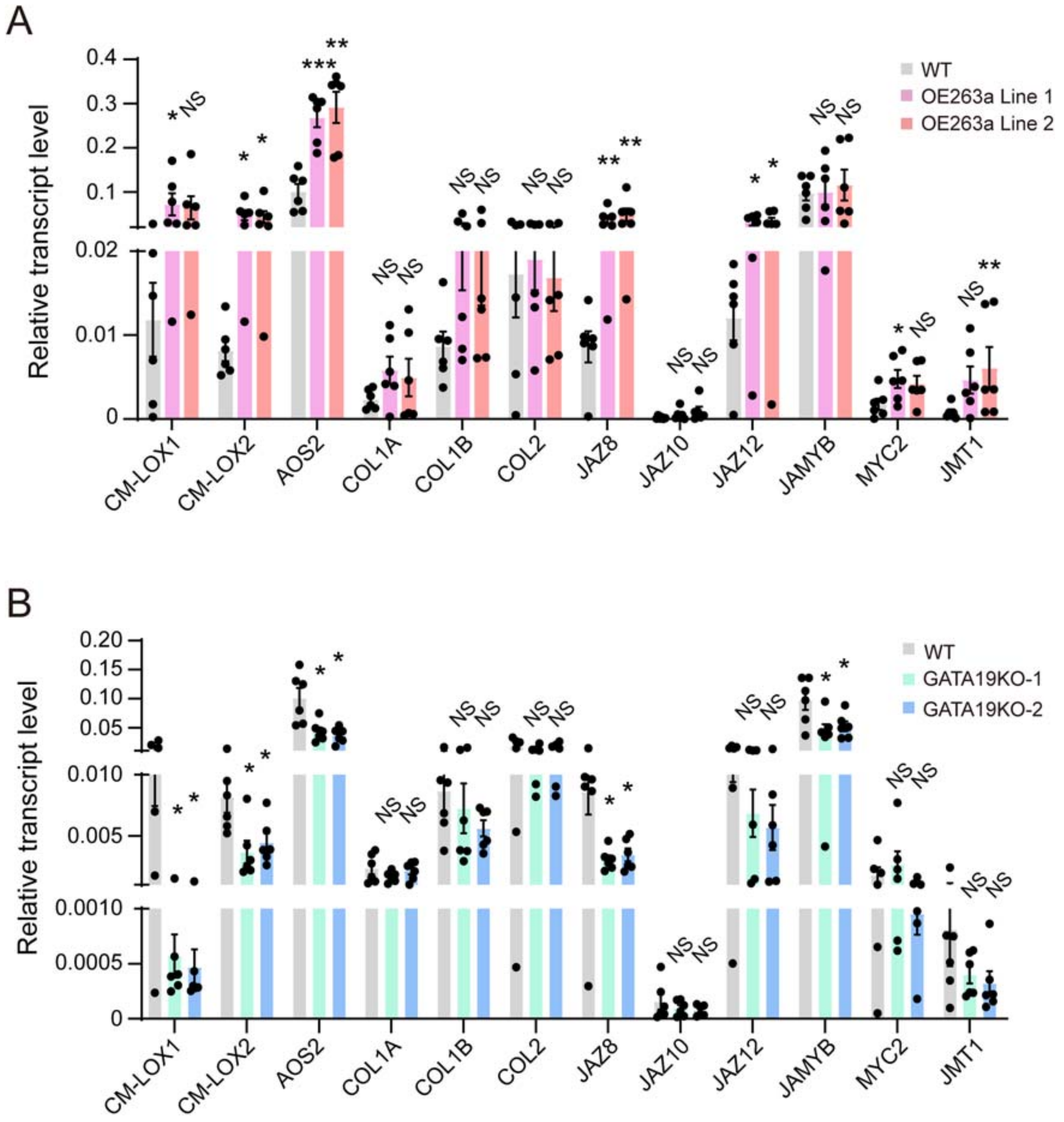

Figure EV5. Relative transcript levels of 12 genes relative to jasmonate signaling pathway in WT and OE263a lines (A), or in WT and *GATA19*KO lines (B).

Six biological replicates were prepared, with each replicate including two leaves. For (**A**), *P* values from left to right, *P* = 0.0395 (*CM-LOX1*), *P* = 0.0171 (*CM-LOX2*), *P* = 0.0476 (*CM-LOX2*), *P* = 1.45E−04 (*AOS2*), *P* = 0.0022 (*AOS2*), *P* = 0.0043 (*JAZ8*), *P* = 0.0022 (*JAZ8*), *P* = 0.0299 (*JAZ12*), *P* = 0.0398 (*JAZ12*), *P* = 0.0400 (*MYC2*), *P* = 0.0087 (*JMT1*). For (**B**), *P* values from left to right, *P* = 0.0260 (*CM-LOX1*), *P* = 0.0260 (*CM-LOX1*), *P* = 0.0152 (*CM-LOX2*), *P* = 0.0296 (*CM-LOX2*), *P* = 0.0129 (*AOS2*), *P* = 0.0134 (*AOS2*), *P* = 0.0260 (*JAZ8*), *P* = 0.0260 (*JAZ8*), *P* = 0.0252 (*JAMYB*), *P* = 0.0354 (*JAMYB*). Values are presented as mean ± SE and were compared by Student's t test. NS, no significant difference. *$P < 0.05$. **$P < 0.01$. ***$P < 0.001$

 © The Author(s)

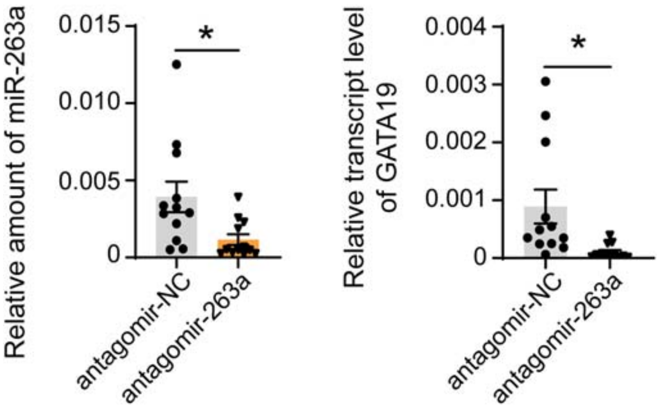

Figure EV6. Relative amount of miR-263a and relative transcript level of *GATA19* in wild-type rice fed upon by nonviruliferous SBPHs with injection of antagomir-263a or NC.

OsU6 snRNA and *UBQ10* serve as internal references for miRNA and gene, respectively. Twelve biological replicates were prepared, with each replicate including two leaves. *P* values from left to right, $P = 0.0202$, $P = 0.0211$. Values are presented as mean ± SE and were compared by Student's t test. $^{*}P < 0.05$.

© The Author(s)
